# Comparative proteomic analysis of four biotechnological strains *Lactococcus lactis* through label‐free quantitative proteomics

**DOI:** 10.1111/1751-7915.13305

**Published:** 2018-10-19

**Authors:** Wanderson M. Silva, Cassiana S. Sousa, Leticia C. Oliveira, Siomar C. Soares, Gustavo F.M.H. Souza, Guilherme C. Tavares, Cristiana P. Resende, Edson L. Folador, Felipe L. Pereira, Henrique Figueiredo, Vasco Azevedo

**Affiliations:** ^1^ Departamento de Biologia Geral Instituto de Ciências Biológicas Universidade Federal de Minas Gerais Belo Horizonte Minas Gerais Brasil; ^2^ Departamento de Microbiologia, Imunologia e Parasitologia Instituto de Ciências Naturais e Biológicas Universidade Federal do Triangulo Mineiro Uberaba Minas Gerais Brasil; ^3^ MS Applications Laboratory Waters Corporation Waters Technologies Brazil Alphaville São Paulo Brasil; ^4^ AQUACEN Escola de Veterinária Universidade Federal de Minas Gerais Belo Horizonte Minas Gerais Brasil; ^5^ Centro de Biotecnologia Universidade Federal da Paraíba João Pessoa Paraíba Brasil

## Abstract

*Lactococcus lactis* is a bacteria with high biotechnological potential, where is frequently used in the amino acid production and production of fermented dairy products, as well as drug delivery systems and mucosal vaccine vector. The knowledge of a functional core proteome is important extremely for both fundamental understanding of cell functions and for synthetic biology applications. In this study, we characterized the *L. lacits* proteome from proteomic analysis of four biotechnological strains *L. lactis*:* L. lactis* subsp. *lactis* NCDO2118, *L. lactis* subsp. *lactis* IL1403, *L. lactis* subsp. *cremoris* NZ9000 and *L. lactis* subsp. *cremoris* MG1363. Our label‐free quantitative proteomic analysis of the whole bacterial lysates from each strains resulted in the characterization of the *L. lactis* core proteome that was composed by 586 proteins, which might contribute to resistance of this bacterium to different stress conditions as well as involved in the probiotic characteristic of *L. lactis*. Kegg enrichment analysis shows that ribosome, metabolic pathways, pyruvate metabolism and microbial metabolism in diverse environments were the most enriched. According to our quantitative proteomic analysis, proteins related to translation process were the more abundant in the core proteome, which represent an important step in the synthetic biology. In addition, we identified a subset of conserved proteins that are exclusive of the *L. lactis* subsp. *cremoris* or *L. lactis* subsp. *lactis*, which some are related to metabolic pathway exclusive. Regarding specific proteome of NCDO2118, we detected ‘strain‐specific proteins’. Finally, proteogenomics analysis allows the identification of proteins, which were not previously annotated in IL1403 and MG1363. The results obtained in this study allowed to increase our knowledge about the biology of *L. lactis*, which contributes to the implementation of strategies that make it possible to increase the biotechnological potential of this bacterium.

## Introduction

The lactic acid bacteria (LAB) comprise a group of Gram positive microorganisms, facultative anaerobics, able to produce lactic acid from the fermentation of carbohydrates and widely used in the industrial food fermentation. *Lactococcus lactis* is the most studied species of this group, and can be classified into different subspecies. Among these subspecies, *L. lactis* subsp*. lactis* and *L. lactis* subsp*. cremoris* can be highlighted. Both of them are frequently used in the amino acid production, as well as in the production of fermented dairy products, such as cheese, buttermilk and sour cream (Song *et al*., 2017). In addition, *L. lactis* presents a biomedical potential great mainly as drug delivery systems and mucosal vaccine vector (reviewed in Wyszyńska *et al*., 2015). Many of the attributions of this bacterium were achieved, thanks to the development of the NIsin‐Controlled gene Expression system (the NICE system) (de Ruyter *et al*., 1996). This efficient system based on the autoregulation mechanism of nisin biosynthesis in *L. lactis* has been allowing the overproduction of a variety of proteins with biotechnological interest (Kuipers *et al*., 1995; de Ruyter *et al*., 1996; Mierau and Kleerebezem, 2005). Thus, the identification of factors that are required for physiology this bacterium is critical for understanding the biology of its biotechnological capacity.

The availability of whole genome sequences of different strains of *L. lactis* has enabled the knowledge about molecular bases of the genetic and biochemistry this bacteria (Oliveira *et al*., 2014; Tschoeke *et al*., 2014; Zhao *et al*., 2015). In addition, these genomic studies contributed also to the development of metabolic model for explore the biotechnological potential of *L. lactis*, through of biology system approach (Flahaut *et al*., 2013; Dolatshahi *et al*., 2016). In complementation to structural studies of the *L. lactis* genome, several proteomic studies have been performed to evaluate its functional genome. During the industrial process, *L. lactis* is exposed to different environments; thus, proteomic analysis regarding osmotic stress (Zhang *et al*., 2010), acid stress (Budin‐Verneuil *et al*., 2005, 2007), oxidative stress (Rochat *et al*., 2012) and cooper stress (Barré *et al*., 2007) contributed to improve the knowledge about factors involved in distinct physiologic pathway related to growth and stability of *L. lactis* in starter cultures. In addition, others proteomic studies also have been conducted to evaluate the metabolic profile of this bacterium (Guillot *et al*., 2003). For example, the characterization of *L. lactis* proteome under industrial environment mimetic conditions, such as milk, revealed different pathways that contribute to *L. lactis* adaptation and growth in the milk (Gitton *et al*., 2005).

Despite all proteomic studies, currently, no work was conducted to characterize the *L. lactis* core proteome. Studies show that the knowledge of a core functional proteome is important extremely for both developing fundamental understanding of cell functions and for synthetic biology applications (Zhang *et al*., 2014; Yang *et al*., 2015). So to better insights about factors related to the physiologic process of *L. lactis* and to complement previous studies related to both structural and functional genome of this bacterium. In this study, we applied *high‐throughput proteomics* using label‐free quantitative approach to identify and quantitate the proteome of four biotechnological strains *L. lactis* that are commonly used in physiologic studies this bacterium. *L. lactis* subsp. *lactis* IL1403 a prototypical strain isolated from a cheese starter culture (Bolotin *et al*., 2001), *L. lactis* subsp. *lactis* NCDO2118 isolated from frozen peas with probiotic characteristic (Luerce *et al*., 2014; Oliveira *et al*., 2014), *L. lactis* subsp. *cremoris* MG1363 plasmid‐free and prophage‐cured derivative of NCDO712, extremely used for physiological studies (Gasson, 1983) and *L. lactis* subsp. *cremoris* NZ9000 used in the production of heterologous proteins trough of the NICE expression system (Linares *et al*., 2010).

## Results and discussion

### Characterization of the *L. lactis* proteome

**Figure 1:**
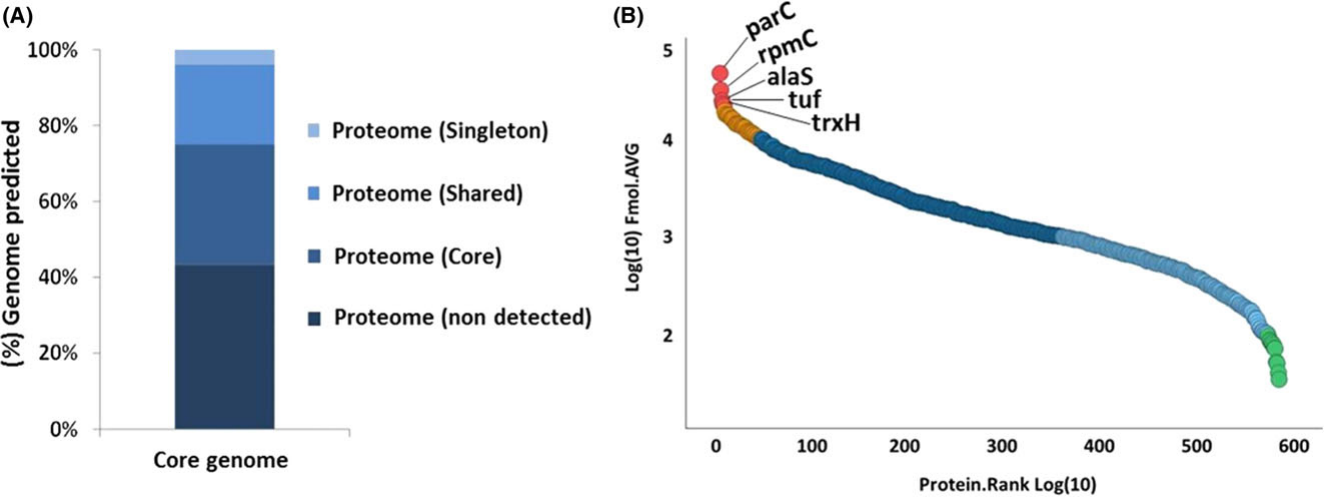
Characterization of the proteome of *L. lactis* and correlation with *in silico* data. A. Correlation of the proteomic results with *in silico* data of the *L. lactis* core genome. B. Dynamic range of the method based on protein abundance estimates, data points derived from LC‐UDMS ^E^ analysis, abundance distribution of all quantified proteins of the *L. lactis* core proteome. Red and orange represent the group of the more abundant proteins and green represent the less abundant proteins.

First, we performed a comparative genomic analysis among the genome of the strains NCDO2118, IL1403, MG1363 and NZ9000 to determine the *L. lactis* core genome. According to this analysis, the core genome is composed by 1,673 genes (Table S1). To determine the *L. lactis* proteome, the four bacterial strains were grown in M‐17 medium, posteriorly the proteins were extracted, digested in solution, and then the tryptic peptides were submitted to LC/MS^E^ analysis. Ion mobility enhanced MS^E^ spectra were derived from three biological runs of each strain subdivided into five fractions and were analysed by Progenesis QI for Proteomics (QIP) v.2.0 (Waters, Milford, CA, USA) for protein identification and label‐free quantification. From this proteomic analysis, we identified 1,108 different proteins of *L. lactis*. These proteins were detected in at least two of the three biological replicates for the four strains analysed, with an average of seven peptides/protein; in addition, these proteins were present in at least two of three biological replicates for the strains utilized. When the repertoire of proteins identified by MS was overlapped against the core genome, we characterized approximately 56% (946 proteins) of the *L. lactis* core genome (Fig. 1A, Table S1). The information about sequence coverage and a number of identified peptides for each protein identified, as well as the information about the native peptide are available at Appendices S1 and S2.

### Functional classification and quantification of *Lactococcus lactis* core proteome

**Figure 2:**
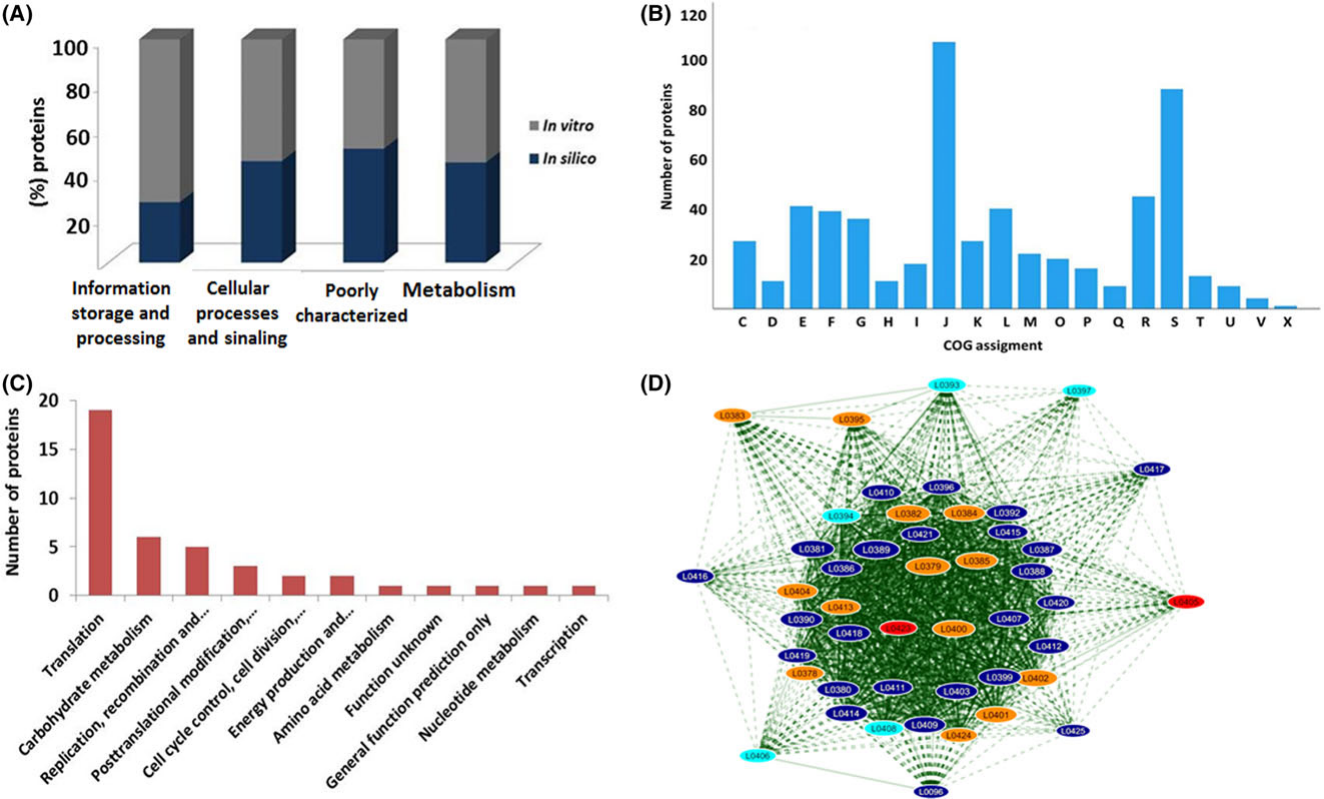
Functional analysis of the *L. lactis* core proteome. A. Comparative analysis between the predicted core genome and all proteins identified by LC‐MS^E^. B. Biological processes that comprise the *L. lactis* core proteome. The COG abbreviations are as follows: [C] energy production and conversion; [D] cell cycle control, cell division and chromosome partitioning; [E] amino acid transport and metabolism; [F] nucleotide transport and metabolism; [G] carbohydrate transport and metabolism; [H] coenzyme transport and metabolism; [I] lipid transport and metabolism; [J] translation, ribosomal structure and biogenesis; [K] transcription; [L] replication, recombination and repair; [M] cell wall/membrane/envelope biogenesis; [O] post‐translational modification, protein turnover and chaperones; [P] inorganic ion transport and metabolism; [Q] secondary metabolite biosynthesis, transport and catabolism; [R] general function prediction only; [S] function unknown; [T] signal transduction mechanisms; [U] intracellular trafficking, secretion and vesicular transport. C. Biological process of the group of more abundant proteins. D. Protein‐protein network of the proteins related to translational process. The network nodes represent proteins, and the edges represent protein‐protein associations. The node size (protein) is proportional to the amount of protein interacting (degree of interaction). The colour of each protein is defined according to the abundance level present in the dynamic range of the Fig. 1B.

From this proteomic analysis, the *L. lactis* core proteome was composed by 585 proteins (Table S1). The functional characteristic of the *L. lactis* core genome and proteome were determined by gene ontology (GO) analysis (Fig. 2A). When evaluated the proteins that comprise only the core proteome in biological process, the proteins were assigned to 20 processes (Fig. 2B). Once proteins are interconnected in groups via protein network to exercise different biological function, the knowledge of these networks is of great importance to understating of the bacterial biology (Typas and Sourjik, 2015). To determine how the proteins of the *L. lactis* core proteome could interact, we used Cytoscape software to generate an interaction network, which was composed by 568 proteins and 8402 interactions (Figure S1).

To increase our knowledge about the biology of *L. lactis* at level biological system, we estimated the concentrations of all proteins identified in the *L. lactis* core genome, using the label‐free Hi3 method, where protein is quantified according to the three most intense peptides. The dynamic range of the proteins identified was determined by sample normalization using ‘scouting runs’ through of the stoichiometry between the intensity and molarity proportion prior to injection. Based in this analysis, the *L. lactis* core proteome spanned a dynamic range of four orders of magnitude between the most and least abundant proteins. We generate a graph of protein amounts of the identified proteins from all samples against protein ranks (Fig. 1B). All of the identified proteins on the protein abundance scale are listed in Table S2. The five proteins that were found to be most abundant include DNA topoisomerase 4 subunit A (*parC*), essential bacterial enzyme responsible for introducing negative supercoils into DNA and for the decatenation of DNA; 50S ribosomal protein L29 (*rpmC*), structural constituent of ribosome involved in the ribosomal large subunit assembly; Alanine–tRNA ligase (*alaS*), proteins involved in the alanyl‐tRNA aminoacylation, translation and tRNA modification; Elongation factor Tu (*tuf*), protein that act both control of translation accuracy as well as protein folding (Caldas *et al*., 1998; Wohlgemuth *et al*., 2011); and Thioredoxin H‐type (*trxH*) that is related to cell redox homeostasis and oxidation‐reduction process.

Conversely, to evaluate the relative difference between the NCDO2118, IL1403, NZ9000 and MG1363 proteome, label‐free quantification was used. Only proteins that presented cut‐off values of twofold change and ANOVA *P*‐value (*P *<* *0.05) were considered as differentially expressed. According to this analysis, using the NCDO2118 strain as reference, we observed that some proteins were either more‐ or less‐induced when compared with others strains (Table S3). Interestingly, among the differentially expressed proteins, 16 proteins were commonly modulated among the three strains when compared with NCDO2118, being eleven more induced and five less induced. These proteins are related to Amino acid transport and metabolism, Inorganic ion transport and metabolism, Coenzyme transport and metabolism, Transcription and General function prediction only. The comparative analysis between IL1403 and NCDO21118 difference were observed in proteins related to inorganic ion transport and metabolism. In turn, when compared the two *L. lactis* subsp. *cremoris* strains MG1363 and NZ9000, we observed that ten proteins were commonly modulated in comparison with NCDO21118, being some proteins related to carbohydrate metabolism, such as the glyceraldehyde‐3‐phosphate dehydrogenase (GPDH) an enzyme extremely important to glycolytic pathway.

### The major functional classes that composed the *L. lactis* core proteome

#### Information storage and processing

The label‐free quantification strategy applied us enabled estimate the more abundant proteins of the *L. lactis* core proteome and promote insights into content protein this bacteria that contribute to it physiological adaptation. The majority of the more abundant proteins that comprise the core proteome are related both ribosomal complexes (structural elements) and translational machinery (related to initiation, elongation and termination steps), which are of great importance to the regulation of the translation process (Gingold and Pilpel, 2011). Through PPI network, we showed the interaction of the proteins related to translation process, which was composed by 46 proteins and 1.184 interactions, these proteins might have an important role in protein synthesis of *L. lactis* (Fig. 2D). Some studies have been performed to enlighten our understanding of translational regulation in *L. lactis*. Van de Guchte *et al*. (1991) showed through of *in silico* analysis that the mRNA secondary structure might contribute to improve the initial efficiency of translation. The characterization of the *L. lactis* translatome allowed determinate the proportion of mRNA involved in translation, as well as its ribosome occupancy and density (Picard *et al*., 2012). Despite these studies, more efforts are needed to implement strategies that make exploring the knowledge of the translational control of this bacterium, which is an important step in the process optimization of synthesis protein as well as in the synthetic biology.

#### Metabolism

To identify which metabolic pathways were overrepresented, Kegg enrichment analysis was performed, and our results revealed that a total of 22 pathways presented significant value (*P *<* *0.05) (Table S4). According to enrichment analysis on KEGG pathways, the *L. lactis* core proteome was strongly enriched in metabolic pathways and microbial metabolism in diverse environments. In case of *L. lactis* that is a bacteria with great industrial relevance the knowledge about factors related to cellular metabolism is a major study focus, because from different metabolic process are produced several products such as proteins, amino acids, organic acids, antibiotics and metabolites for the food industry (Voit *et al*., 2006; Murabito *et al*., 2014).

*Lactococcus lactis* is exposed to different stress conditions, such as acid stress, osmotic stress, thermal stress, oxidative stress and nutritional starvation, these perturbation have a direct effect in its physiologic state (Papadimitriou *et al*., 2016). Thus, to confront the environmental changes, *L. lactis* need adjust its metabolism to adapt and survive the different stress conditions that are commonly found by this bacterium both in industrial fermentation and in the human digestive tract. In the *L. lactis* core proteome, we identified proteins that have a play role in the Lactococcus metabolism under stress condition, such as LDH, lactate dehydrogenase; ACK, acetate kinase; PdhABCD, pyruvate dehydrogenase complex, alpha‐ASL, alpha‐acetolactate synthase; alpha‐ALD, alpha‐acetolactate decarboxylase, AdhE, alcohol dehydrogenase and NOX, NADH oxidase (Papadimitriou *et al*., 2016). Acid stress is commonly found by *L. lactis* both in industrial fermentation and in the human digestive tract. A study conducted by Carvalho *et al*. (2013) shown that acid stress has effect in glycolytic pathway of *L. lactis*, where the glucose consumption by this bacterium is reduced when exposed to low pH. On the other hand, different metabolic processes have an important play role in the adaptation to acid stress, as purine metabolism, that was enriched in our Kegg analysis (Rallu *et al*., 2000). Purine metabolism also contributes to *L. lactis* adaptation to others type of stress such as heat‐shock and oxidative stress (Rallu *et al*., 2000).

The proteins identified in our proteomic analysis also were identified in the Monte Carlo model (Murabito *et al*., 2014) that provide a stoichiometric representation of the central carbon metabolism of enzyme involved in fermentative metabolism of *L. lactis*. Pyruvate metabolism that was strongly enrichment according to KEGG analysis is highly studied in *L. lactis*, where some metabolic engineering strategies have been developed from this pathway for example the efficient production of the amino acid alanine (Hols *et al*., 1999) or diacetyl compound that is utilized in dairy products, such as butter, buttermilk and cheeses (Hugenholtz *et al*., 2000). We identified two important protein related to pyruvate metabolism pyruvate kinase (PYK) and lactate dehydrogenase (LDH). PYK was detected among the more abundant proteins of the *L. lactis* core proteome; this enzyme is an allosterically regulated that links glycolysis, the primary energy metabolism, to cellular metabolism (Murabito *et al*., 2014). In addition, PYK has an important role in the carbon flux, however, only when the glucose level is limited (Ramos *et al*., 2004). LDH is extremely important to lactic acid fermentation, this enzyme reduces pyruvate to lactate although oxidation of NADH. Studies have been showed that LDH contributes both to growth of *L. lactis* and in the ability of this bacterium to utilize different substrates as carbon source (Griffin *et al*., 1992; Fiedler *et al*., 2011).

#### Cellular processes and signalling

The fresh fermentation products or dried bacterial supplements are forms commonly used in the formulation of probiotics. However, before the drying process, probiotic bacteria are grown under temperature elevated during spray‐drying and temperature low during the freeze‐drying and storage. In addition, during the drying process, these bacteria also exposed to osmotic stress, dehydration and oxidative stress. In the *L. lactis* core proteome, we have identified a subset of proteins related to post‐translational modification and protein turnover, which contribute to cellular integrity and physiologic process bacterial during this abrupt temperature variation, osmotic stress and oxidative stress. Our proteomic analysis identified the two main conserved chaperone complexes, the DnaK‐DnaJ‐GrpE and GroEL‐GroES. Proteomic analysis of MG1363 after exposition to heat stress and salt stress showed that heat‐shock proteins DnaK, GroEL and GroES were highly induced (Kilstrup *et al*., 1997). Already in *Lactobacillus paracasei* NFBC 338 has been demonstrated that *groESL* operon contribute to viability this strain during the drying process (Corcoran *et al*., 2006).

One of the hallmark characteristics of probiotic bacteria is its ability to colonize and persist in the intestinal epithelial, this process is mediated by adhesive proprieties these bacteria. The adhesion cellular process enhances the probiotic effect this interaction between the microbe‐host stimulates the immunomodulation process, as well as protects the intestinal barrier against pathogens (Bron *et al*., 2011). We have identified proteins as basic membrane protein A, Chitinase, Exodeoxyribonuclease, Pyruvate carboxylase fibronectin‐binding protein, which contribute to adhesion cellular of *L. lactis* (Radziwill‐Bienkowska *et al*., 2016; Oliveira *et al*., 2017). In addition, the *L. lactis* core proteome is composed by components important of the cell wall this bacterium related to peptidoglycan, capsular polysaccharide (CPS), lipoproteins and teichoic acids, which contain specific structures that are recognized by Toll‐like receptor present in the host intestinal mucosa (Bron *et al*., 2011).

### Composition of the shared genome/proteome

According to the analysis performed in our study, the ‘core genome’ was established by a gene detected in the genome of the four strains (Table S1). On the other hand the ‘shared genome’ was composed by genes that are in two or three strains (Table S5). Thus in our proteomic analysis were detected 117 proteins that comprise the shared genome (Table S5). Interestingly from this analysis, we detected 53 proteins that were shared only between NZ9000 and MG1363 that are *L. lactis* subsp. *cremoris* strains and 23 proteins were shared only between the *L. lactis* subsp. *lactis* strains NCDO2118 and IL1403. The majority of the proteins detected both *L. lactis* subsp. *cremoris* and *L. lactis* subsp. *lactis* are classified as poorly characterized or of unknown function. When the proteins of known or predicted function were analysed by KEGG pathway in the *L. lactis* subsp. *cremoris* we detected two proteins related to carbohydrate metabolism; beta‐glucosidase that is involved in the conversion of cellobiose to glucose and UDP‐glucose 6‐dehydrogenase that is associated to polysaccharide biosynthesis, this enzyme converts UDP‐glucose to UDP‐glucuronate. In addition to these proteins described above, a glutamyl tRNA synthetase was also detected *in L. lactis* subsp. cremoris strains, this protein this involved in the L‐glutamyl‐tRNA (Glu) biosynthesis. In *L. lactis* subsp. *lactis* strains, we also detected proteins related to carbohydrate metabolism as UDP‐glucose 4‐epimerase and Lacto‐*N*‐biosidase, which are involved in the galactose and lactose metabolism, respectively. In *Bifidobacterium bifidum* a study showed that Lacto‐*N*‐biosidase plays an important role in the degradation process of Human Milk Oligosaccharides (HMOs) (Wada *et al*., 2008). HMOs are considered like ‘bifidus factor’, which contribute to the growth of *Bifidobacterium* in infant intestinal tract and consequently to probiotic characteristic this bacterium mainly in breast‐fed infants (Asakuma *et al*., 2011; Aakko *et al*., 2017). This observation motivates further studies to evaluate whether *L. lactis* might growth in the presence of HMOs, beside explore its role in the physiology this bacterium.

### Composition of the exclusive proteome

Our proteomic analysis detected proteins in the exclusive proteome of the strains: IL1403 (15 proteins), NZ9000 (11 proteins) and NCDO2118 (38 proteins) related to different biological process (Table S1). When compared these proteins with *in silico* data of the genomes of each strain the *open reading frame* (*ORF*) that codify these proteins are present in the genome of all strains, in other words, these proteins belong to ‘core genome’. Conversely in NCDO2118, in addition to these 38 proteins, another 39 proteins were also detected in its exclusive proteome (Table S6). However, these proteins were detected like strain‐specific proteins, due to the *ORF* encoding these proteins not were detected in the genome of the others strains. Among these strain‐specific proteins, 16 proteins are assigned as function unknown. The other proteins are mainly related to transcription process such as XRE family transcriptional regulator, LysR family transcriptional regulator, GntR family transcriptional regulator and Cro/Cl family transcriptional regulator that are involved in metabolism, quorum sensing, motility and stress response. We also detected important proteins related to the sucrose metabolism such as Sucrose‐6‐phosphate hydrolase (S6PH) and PTS sucrose transporter subunit IIABC. The PTS system enables that NCDO2118 import extracellular sucrose producing Sucrose‐6‐phosphate, posteriorly this product is hydrolyzed by S6PH to glucose 6‐phosphate and fructose. While glucose 6‐phosphate can directly be used, the fructose need be phosphorylated by an ATP‐dependent fructokinase before be conducted to glycolytic pathway (Rauch and de Vos, 1992; Luesink *et al*., 1999).

### Proteogenomic analysis

For detected proteins that were not previously annotated in the *L. lactis* subsp. *lactis* strains, we created a concatenated database composed by genome annotation of NCDO2118 (Accession Number: CP009054) and IL1403 (Accession Number: AE005176) and contrasted against the measured MS/MS spectra from proteomic dataset of NCDO2118 and IL1403. From this analysis, we detected 19 proteins in IL1403 that were not previously annotated. Regard to *L. lactis* subsp. *cremoris* strains a concatenated database was created from genome annotation of MG1363 (Accession Number: AM406671) and NZ9000 (Accession Number: CP002094). When contrasted against the measured MS/MS spectra from proteomic dataset of MG1363 and NZ9000 were detected three proteins in MG1363, which were not previously annotated. All parameters, as well as, the peptides sequence which were used for identification of these proteins are shown in Table S1.

## Concluding remarks

Systems biological approaches are based in the broad range of large‐scale data collection, these dataset obtained from ‘omics’ approaches associated with computational tool has promote insights in the dynamic behaviour of a system (Voit *et al*., 2006; Yang *et al*., 2015). In this present study, we combined genomic dataset, *high‐throughput proteomic* and PPI network analysis for characterized the *L. lactis* proteome, which we enabled create a link between genotype and phenotype this bacteria. We have identified a proteins subset conserved involved in different biological function that contribute to the basic life cycle of *L. lactis* as well as to adaptation in environmental distinct. In addition, we demonstrate through our proteomic analysis that the majority of the more abundant proteins of *L. lactis* core proteome are related in the translation process, step important to synthetic biology. Finally, these results might contribute to rational design and optimization of industrial bioprocesses, mainly in the starter culture and fermentation, as well as in the development of strategies for the overexpression of heterologous genes so that *L. lactis* becomes a probiotic bacterium.

## Experimental procedures

### Bacterial strain, growth conditions and preparation of *L. lactis* protein extract

The strains IL1403, NCDO2118, MG1363 and NZ9000 (Table 1) were grown in M17 synthetic media, the main medium culture to the growth of *L. lactis* (Terzaghi and Sandine, 1975) and incubated at 30°C in aerobiose, without agitation for 16 h (stationary phase). The preculture were then inoculated (1:100) in M17 fresh medium (Difco, Franklin Lakes, NJ, USA) supplemented with 0.5% (w/v) glucose (M17Glc) at 30°C until reach OD_600_ = 0.8 (three independent experiments). Posteriorly, NCDO2118, IL1403, MG1363 and NZ9000 cultures were centrifuged at 4000 ***g*** for 10 min at 4°C. The cell pellets were washed in phosphate buffer saline and then resuspended in 1 ml of lysis buffer (7 M urea, 2 M thiourea, 4% (w/v) CHAPS, and 1 M dithiothreitol (DTT)). The cells were sonicated using five 1 min cycles on ice and bacterial lysates were centrifuged at 14 000 g for 40 min at 4°C. The protein extract from three biological replicates of each strain was concentrated using a spin column with a 10 kDa threshold (Millipore, Billerica, MA, USA). After this process, the proteins were then denatured with the addition of 0.1% *Rapi*GEST SF (Waters), reduced with 10 mM DTT and alkylated by the addition of 10 mM iodoacetamide. Finally, the proteins were enzymatically digested with trypsin (50 ng μl^−1^) (Promega) at 37°C overnight and the digestion process was stopped by adding 10 μl of trifluoroacetic acid (TFA) 5% v/v (Fluka, Buchs, Germany). The resulting peptide extracts were centrifuged at 21 900 ***g*** for 30 min at 6°C. The supernatants were collected, transferred to Waters Total Recovery vials (Waters), added 5 μl of 1 N ammonium hydroxide (Sigma Alldrich, St. Louis, Missouri, USA) and stored at −70°C until use.

**Table 1:** Strains used in this study

Strains	Description	
MG1363	*L. lactis* subsp. *cremoris*, plasmid‐free and prophage‐cured derivative of NCDO712	Gasson (1983)
		Linares *et al*. (2010)
NCDO2118	*L. lactis* subsp. *lactis* isolated from frozen peas	Oliveira *et al*. (2014)
NZ9000	*L. lactis* subsp. *cremoris* used in the production of heterologous proteins trough of the NICE expression system	Linares *et al*. (2010)
IL1403	*L. lactis* subsp. *lactis* a prototypical strain isolated from a cheese starter culture	Bolotin *et al*. (2001)

## 2D nanoUPLC‐UDMS^E^ data acquisition

Qualitative and quantitative bidimensional analysis was performed using a two‐dimensional reversed phase (2D RPxRP) nanoUPLC‐MS (Nano Ultra Performance Liquid Chromatography Mass Spectrometry) approach with multiplexed ultra‐definition mass spectrometry (UDMS^E^) label‐free quantitation. Prior to proteomic analysis, a stoichiometric measurement based on scouting runs of the integrative total ion account (TIC) were performed with all samples to ensure standardized molar values across all conditions. The experiments were conducted using a nanoACQUITY UPLC 2D RPxRP system, with 1‐h reverse‐phase gradient from 7% to 40% (v/v) acetonitrile (0.1% v/v formic acid) at 500 nl min^−1^ (Gilar *et al*., 2005). A nanoACQUITY UPLC HSS T3 1.8 μm, 75 μm × 15 cm column (pH 3) was used with a RP XBridge BEH130 C18 5 μm 300 μm x 50 mm nanoflow column (pH 10). Typical on‐column sample loads were 250 ng of the total protein digests for each of the five fractions (250 ng fraction^−1^ load^−1^). All analyses were performed using nano‐electrospray ionization in the positive ion mode nanoESI (+) and a NanoLockSpray (Waters, Manchester, UK) ionization source. The mass spectrometer was calibrated using a MS/MS spectrum of [Glu^1^]‐Fibrinopeptide B human (Glu‐Fib) solution (100 fmol μl^−1^) delivered through the NanoLockSpray source reference sprayer, multiplexed data‐independent (DIA) scanning with additional specificity and selectivity for non‐linear ‘T‐wave’ ion mobility (UDMS^E^) experiments were performed using a Synapt G2‐Si HDMS mass spectrometer (Waters).

### Processing of mass spectral data

For the protein identification and the quantitative analyzes were utilized dedicated algorithms (Geromanos *et al*., 2009) and a database using the default parameters to account for ions (Li *et al*., 2009). Databases reverse ‘on‐the fly’ were utilized during the database queries and appended to the original database to assess the false‐positive identification rate. For proper spectra processing and database searching condition, the Progenesis QI for Proteomics (QIP) v.2.0 (Waters) was used. The raw data obtained were visualized and analysed without missing values. The 2D ion intensity maps showed retention time, m/z, intensity, as well as mass spectra and chromatogram views, which provided quality of the automatic alignment, peak picking and compound deconvolution on every run. For peptide identification, data were searched against *L. lactis in‐house* database of whole genomes previously sequenced and annotated of strains used in this study. *In‐house* database was obtained from ‘.faa files’ containing amino acid sequences derived from all the coding sequences (CDS) present in each genome of *L. lactis*. Sequences were exported from ‘.gbk files’ using software Artemis v.16.0.0 (Sanger Institute, Hinxton, Cambridgeshire, UK) (Rutherford *et al*., 2000). The protein sequences of all CDSs from each genome of *L. lactis* were pooled and clustered by CD‐HIT software (Li and Godzik, 2006), using a parameter of 100% similarity to create ‘.fasta file’, with a subset of non‐redundant sequences, retrieving a pan‐proteome database (Broadbent *et al*., 2016).

The search conditions were: maximum allowed missed cleavages by trypsin be up to 1; maximum protein mass = 600 kDA, modifications by carbamidomethyl of cysteine (C) (fixed), acetyl *N*‐terminal (variable), and oxidation of methionine (variable); peptide tolerance of 10 ppm, fragment mass error tolerance of 20 ppm and a default maximum false discovery rate (FDR) value of 4%. The proteins obtained were organized by software algorithm and into a statistically significant list corresponding to increased and decreased regulation ratios. The protein‐level quantitation was performed with Relative Quantitation using Hi‐N algorithm, which is incorporated in Progenesis QIP, using the recommended default parameters. Protein abundance was calculated using the Hi3 methodology (Li *et al*., 2009). The quantitation values were averaged over all samples and the standard deviations of ANOVA *P *≤* *0.05 was utilized.

### Bioinformatics analysis

The genome sequences of *L. lactis* NCDO 2118, IL1403, MG1363 and NZ900 were compared aiming to identify homologous proteins among them using orthoMCL software (Li *et al*., 2003). This software clusters the coding sequences (CDs) according to the degree of homology between genes in the genomes. After clustering, we used in‐house scripts to retrieve the subsets: CDSs shared by all *L. lactis*, which were identified as core genome; the accessory ones that are shared by two or more species, but not by all of them; and, the singletons, which represented the strain‐specific CDSs. The identified proteins after proteomic analysis were subjected to the bioinformatics analysis using prediction tools. Protein functions were assigned by the COG database (Tatusov *et al*., 2001). The protein‐protein interaction network was generated using Cytoscape version 2.8.3 with a spring‐embedded layout (Shannon *et al*., 2003). Kyoto Encyclopedia of Genes and Genomes (KEGG) database was used to identify enriched pathways (Kanehisa and Goto, 2000).

## Conflict of interest

None declared.

## Supporting information


**Fig. S1.** Protein‐protein network of the *L. lactis* core‐proteome.

**Table S1.** Total list of gene that comprise the core genome and proteins identified by LC/MS^E^.

**Table S2.** Total list of identified proteins on the protein abundance scale.

**Table S3.** Total list of differentially expressed proteins.

**Table S4.** KEGG pathway enrichment analysis.

**Table S5.** Total list of proteins detected in the Shared genome/proteome.

**Table S6.** Proteins unique identified in NCDO2118.

**Table S7.** Proteins identified in IL1403 by Proteogenomic analysis.
**Table S8.** Proteins identified in MG1363 by Proteogenomic analysis.

**Appendix S1.** Total list of peptide and proteins identified in IL1403 and NCDO2118.

**Appendix S2.** Total list of peptide and proteins identified in MG1363 and NZ9000.
