# Supplementary figures and images for: Comparative proteomic analysis of four biotechnological strains Lactococcus lactis through label‐free quantitative proteomics
Source: Microb Biotechnol. 2018 Oct 19;12(2):265–74. doi: 10.1111/1751-7915.13305 (PMC6389847; doi:10.1111/1751-7915.13305)

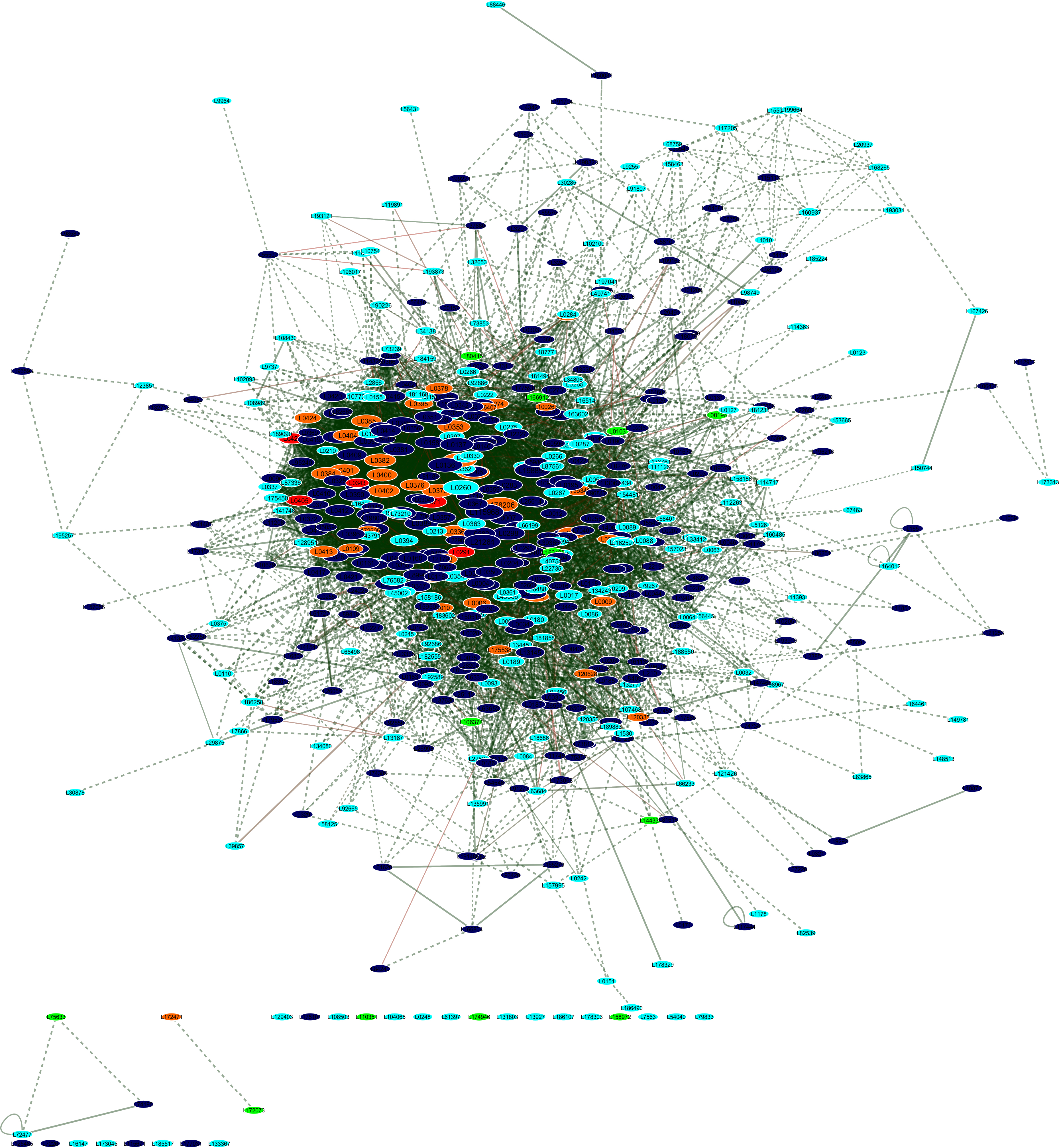

Supplement: Supplementary file 1 — Fig. S1. Protein‐protein network of the L. lactis core‐proteome. [file MBT2-12-265-s001.jpg]
