# Supplementary material for: Comparative proteomic analysis of four biotechnological strains Lactococcus lactis through label‐free quantitative proteomics
Source: Microb Biotechnol. 2018 Oct 19;12(2):265–74. doi: 10.1111/1751-7915.13305 (PMC6389847; doi:10.1111/1751-7915.13305)
Supplement: Supplementary file 2 — Table S1. Total list of gene that comprise the core genome and proteins identified by LC/MSE. [file MBT2-12-265-s002.pdf]

Supplementary Table 1: Total list of gene that comprise the core genome and proteins identified by LC/MS^E.

Genome				Ortology	Description	Expression	COG assignment	Proteins identified by LC/MSE			
MG1363	NZ9000	NCDO2118	IL1403					MG1363	NZ9000	NCDO2118	IL1403
llmg_1118	LLNZ_05780	NCDO2118_RS07190	L0002	core	ATP-dependent 6-phosphofructokinase	Core	G	x	x	x	x
llmg_1119	LLNZ_05785	NCDO2118_RS07185	L0003	core	Pyruvate kinase	Core	C	x	x	x	x
llmg_0530	LLNZ_02740	NCDO2118_RS02820	L0004	core	glyceraldehyde-3-phosphate dehydrogenase	Core	G	x	x	x	x
llmg_2539	LLNZ_13115	NCDO2118_RS12385	L0005	core	Glyceraldehyde-3-phosphate dehydrogenase	Core	G	x	x	x	x
llmg_1424	LLNZ_07340	NCDO2118_RS05900	L0006	core	Triosephosphate isomerase	Core	G	x	x	x	x
llmg_0617	LLNZ_03175	NCDO2118_RS03300	L0007	core	Enolase	Core	G	x	x	x	x
llmg_2167	LLNZ_11185	NCDO2118_RS10530	L0009	core	Fructose-bisphosphate aldolase	Core	G	x	x	x	x
llmg_0253	LLNZ_01310	NCDO2118_RS01235	L0010	core	Phosphoglycerate kinase	Core	G	x	x	x	x
llmg_0355	LLNZ_01860	NCDO2118_RS01995	L0011	core	2,3-bisphosphoglycerate-dependent phosphoglycerate mutase	Core	G	x	x	x	x
llmg_2448	LLNZ_12660	NCDO2118_RS11865	L0012	core	Glucose-6-phosphate isomerase	Core	G	x	x	x	x
llmg_1339	LLNZ_06925	NCDO2118_RS05995	L00157	core	GTP-binding protein	Core	J	x	x	x	x
llmg_1114	LLNZ_05760	NCDO2118_RS07215	L0016	core	Glycerol-3-phosphate dehydrogenase	Core	C	x	x	x	x
llmg_1120	LLNZ_05790	NCDO2118_RS07180	L0017	core	Lactate dehydrogenase	Core	G	x	x	x	x
llmg_0776	LLNZ_04030	NCDO2118_RS09045	L00196	core	Ferredoxin--NADP reductase	Core	O	x	x	x	x
llmg_2233	LLNZ_11510	NCDO2118_RS10880	L0024	core	UDP-glucose 4-epimerase	Core	G	x	x	x	x
llmg_2235	LLNZ_11520	NCDO2118_RS10900	L0028	core	Galactokinase	Core	G	x	x	x	x
llmg_1569	LLNZ_08080	NCDO2118_RS05335	L0032	core	Phosphofructokinase	Core	G	x	x	x	x
llmg_0074	LLNZ_00365	NCDO2118_RS00230	L0033	core	Pyruvate dehydrogenase E1 subunit alpha	Core	C	x	x	x	x
llmg_0073	LLNZ_00360	NCDO2118_RS00225	L0034	core	2-oxoisovalerate dehydrogenase subunit beta	Core	C	x	x	x	x
llmg_0072	LLNZ_00355	NCDO2118_RS00220	L0035	core	dihydrolipoamide acetyltransferase	Core	C	x	x	x	x
llmg_0071	LLNZ_00350	NCDO2118_RS00215	L0036	core	Dihydrolipoamide dehydrogenase	Core	C	x	x	x	x
llmg_0868	LLNZ_04465	NCDO2118_RS08915	L0043	core	Transketolase	Core	G	x	x	x	x
llmg_2499	LLNZ_12910	NCDO2118_RS12265	L0044	core	Glucose-6-phosphate 1-dehydrogenase	Core	G	x	x	x	x
llmg_2511	LLNZ_12970	NCDO2118_RS12320	L0045	core	Ribose 5-phosphate isomerase	Core	G	x	x	x	x
llmg_0586	LLNZ_03005	NCDO2118_RS03135	L0046	core	6-phosphogluconate dehydrogenase	Core	G	x	x	x	x
llmg_1926	LLNZ_09915	NCDO2118_RS09595	L0057	core	3-phosphoshikimate 1-carboxyvinyltransferase	Core	E	x	x	x	x
llmg_1934	LLNZ_09965	NCDO2118_RS09635	L0059	core	Chorismate synthase	Core	E	x	x	x	x
llmg_1938	LLNZ_09985	NCDO2118_RS09655	L0060	core	3-dehydroquinate synthase	Core	E	x	x	x	x
llmg_0125	LLNZ_00635	NCDO2118_RS00450	L0063	core	phospho-2-dehydro-3-deoxyheptonate aldolase	core	E	x	x	x	x
llmg_1227	LLNZ_06320	NCDO2118_RS06665	L0064	core	Phospho-2-dehydro-3-deoxyheptonate aldolase	Core	E	x	x	x	x
llmg_0563	LLNZ_02890	NCDO2118_RS03030	L0082	core	Serine hydroxymethyltransferase	Core	E	x	x	x	x
llmg_0566	LLNZ_02905	NCDO2118_RS03045	L0084	core	3-phosphoglycerate dehydrogenase	Core	H	x	x	x	x
llmg_1181	LLNZ_06080	NCDO2118_RS06845	L0086	core	Branched-chain amino acid aminotransferase	Core	E	x	x	x	x
llmg_0508	LLNZ_02625	NCDO2118_RS02635	L0088	core	Cysteine synthase	Core	E	x	x	x	x
llmg_1775	LLNZ_09160	NCDO2118_RS04115	L0089	core	Cysteine synthase	Core	E	x	x	x	x
llmg_2387	LLNZ_12330	NCDO2118_RS11535	L0092	core	Threonine synthase	Core	E	x	x	x	x
llmg_0874	LLNZ_04500	NCDO2118_RS08890	L0093	core	4-hydroxy-tetrahydrodipicolinate synthase	Core	E	x	x	x	x
llmg_0940	LLNZ_04835	NCDO2118_RS08175	L0094	core	Dihydrodipicolinate reductase	Core	E	x	x	x	x
llmg_2506	LLNZ_12945	NCDO2118_RS12295	L0095	core	Asparagine synthase	Core	E	x	x	x	x
llmg_0098	LLNZ_00490	NCDO2118_RS00335	L0096	core	50S ribosomal protein L33	Core	J	x	x	x	x
llmg_1704	LLNZ_08785	NCDO2118_RS04390	L0103	core	Alanine racemase	Core	M	x	x	x	x
llmg_2312	LLNZ_11940	NCDO2118_RS11170	L0109	core	Ornithine carbamoyltransferase	Core	E	x	x	x	x
llmg_2315	LLNZ_11955	NCDO2118_RS11190	L0110	core	Arginine repressor	Core	K	x	x	x	x
llmg_0886	LLNZ_04560	NCDO2118_RS08830	L0116	core	Gamma-glutamyl phosphate reductase	Core	E	x	x	x	x
llmg_2484	LLNZ_12835	NCDO2118_RS12170	L0118	core	Glutamine synthetase	Core	E	x	x	x	x
llmg_1187	LLNZ_06110	NCDO2118_RS06815	L0120	core	Glutamate racemase	Core	M	x	x	x	x
llmg_1068	LLNZ_05520	NCDO2118_RS07470	L0123	core	Two-component system regulator	Core	T	x	x	x	x
llmg_0414	LLNZ_02160	NCDO2118_RS02280	L0125	core	Two-component system regulator	Core	T	x	x	x	x
llmg_1648	LLNZ_08480	NCDO2118_RS04685	L0127	core	Two-component system regulator	Core	T	x	x	x	x
llmg_0908	LLNZ_04665	NCDO2118_RS08445	L0131	core	Two-component system regulator	Core	T	x	x	x	x
llmg_0747	LLNZ_03890	NCDO2118_RS09195	L0135	core	Two-component system regulator	Core	L	x	x	x	x
llmg_2354	LLNZ_12170	NCDO2118_RS11380	L0136	core	DNA-directed RNA polymerase subunit alpha	Core	L	x	x	x	x
llmg_1982	LLNZ_10235	NCDO2118_RS09880	L0137	core	DNA-directed RNA polymerase subunit beta	Core	L	x	x	x	x
llmg_1981	LLNZ_10230	NCDO2118_RS09875	L0138	core	DNA-directed RNA polymerase subunit beta'	Core	L	x	x	x	x
llmg_0521	LLNZ_02695	NCDO2118_RS02790	L0139	core	RNA polymerase sigma factor SigA	Core	K	x	x	x	x
llmg_0610	LLNZ_03130	NCDO2118_RS03280	L0140	core	Transcription elongation factor GreA	Core	J	x	x	x	x
llmg_0608	LLNZ_03120	NCDO2118_RS03270	L0141	core	DNA-directed RNA polymerase subunit delta	Core	L	x	x	x	x
llmg_0775	LLNZ_04025	NCDO2118_RS09050	L0143	core	Catabolite control protein A	Core	K	x	x	x	x
llmg_1065	LLNZ_05505	NCDO2118_RS07485	L0151	core	GntR family transcriptional regulator	Core	K	x	x	x	x
llmg_0371	LLNZ_01940	NCDO2118_RS02070	L0155	core	GTPase Era	Core	F	x	x	x	x
llmg_1805	LLNZ_09295	NCDO2118_RS03980	L0156	core	GTPase Der	Core	J	x	x	x	x
llmg_2521	LLNZ_13015	NCDO2118_RS12365	L0157	core	tRNA modification GTPase	Core	O	x	x	x	x
llmg_0878	LLNZ_04520	NCDO2118_RS08870	L0158	core	signal recognition particle protein	Core	R	x	x	x	x
llmg_1463	LLNZ_07540	NCDO2118_RS05785	L0159	core	Elongation factor 4	Core	J	x	x	x	x
llmg_0915	LLNZ_04700	NCDO2118_RS08410	L0160	core	GTPase Obg	Core	R	x	x	x	x
llmg_0007	LLNZ_00035	NCDO2118_RS00035	L0161	core	GTP-binding protein	Core	J	x	x	x	x
llmg_0233	LLNZ_01205	NCDO2118_RS01115	L0162	core	GTP-binding protein	Core	J	x	x	x	x
llmg_1831	LLNZ_09420	NCDO2118_RS03845	L0171	core	1,4-dihydroxy-2-naphthoyl-CoA synthase	Core	R	x	x	x	x
llmg_1334	LLNZ_06900	NCDO2118_RS06020	L0177	core	Tetrahydrofolate synthase	Core	Q	x	x	x	x
llmg_1778	LLNZ_09175	NCDO2118_RS04100	L0180	core	Acetyl-CoA carboxylase carboxyltransferase subunit beta	Core	I	x	x	x	x
llmg_1776	LLNZ_09165	NCDO2118_RS04110	L0181	core	Cystathionine beta-lyase	Core	E	x	x	x	x
llmg_1786	LLNZ_09210	NCDO2118_RS04065	L0183	core	Acyl carrier protein	Core	R	x	x	x	x
llmg_1785	LLNZ_09205	NCDO2118_RS04070	L0184	core	ACP S-malonyltransferase	Core	R	x	x	x	x
llmg_1784	LLNZ_09200	NCDO2118_RS04075	L0185	core	3-ketoacyl-ACP reductase	Core	I	x	x	x	x
llmg_1783	LLNZ_09195	NCDO2118_RS04080	L0186	core	3-oxoacyl-ACP synthase	Core	I	x	x	x	x
llmg_1782	LLNZ_09190	NCDO2118_RS04085	L0187	core	Acetyl-CoA carboxylase biotin carboxyl carrier protein	Core	I	x	x	x	x
llmg_1781	LLNZ_09185	NCDO2118_RS04090	L0188	core	3-hydroxyacyl-ACP dehydratase	Core	I	x	x	x	x
llmg_1779	LLNZ_09180	NCDO2118_RS04095	L0189	core	Acetyl-CoA carboxylase biotin carboxylase	Core	I	x	x	x	x
llmg_1777	LLNZ_09170	NCDO2118_RS04105	L0190	core	Acetyl-CoA carboxylase subunit alpha	Core	I	x	x	x	x
llmg_0779	LLNZ_04045	NCDO2118_RS09030	L0195	core	Thioredoxin	Core	O	x	x	x	x
llmg_1588	LLNZ_08175	NCDO2118_RS05245	L0196	core	Thioredoxin reductase	Core	O	x	x	x	x
llmg_1470	LLNZ_07570	NCDO2118_RS05755	L0203	core	NAD synthetase	Core	H	x	x	x	x
llmg_0021	LLNZ_00100	NCDO2118_RS00110	L0204	core	ATP-dependent zinc metalloprotease	Core	D	x	x	x	x
llmg_1546	LLNZ_07945	NCDO2118_RS05405	L0205	core	Cell division protein FtsE	Core	D	x	x	x	x
llmg_1744	LLNZ_09010	NCDO2118_RS04260	L0206	core	Cell division protein FtsY	Core	R	x	x	x	x
llmg_2061	LLNZ_10610	NCDO2118_RS10335	L0207	core	Cell division protein FtsA	Core	D	x	x	x	x
llmg_2060	LLNZ_10605	NCDO2118_RS10330	L0208	core	Cell division protein FtsZ	Core	D	x	x	x	x
llmg_0914	LLNZ_04695	NCDO2118_RS08415	L0209	core	Celldivision protein DivIB	Core	D	x	x	x	x
llmg_0102	LLNZ_00510	NCDO2118_RS00350	L0210	core	Chromosome partitioning protein ParB	Core	D	x	x	x	x
llmg_0113	LLNZ_00565	NCDO2118_RS00400	L0213	core	Sensor protein kinase	Core	O	x	x	x	x
llmg_0528	LLNZ_02730	NCDO2118_RS02810	L0221	core	ATP-dependent Clp protease ATP-binding subunit ClpE	Core	O	x	x	x	x
llmg_0615	LLNZ_03165	NCDO2118_RS03290	L0222	core	ATP-dependent Clp protease ATP-binding protein	Core	O	x	x	x	x
llmg_2288	LLNZ_11825	NCDO2118_RS11055	L0224	core	Acetate kinase	Core	C	x	x	x	x
llmg_0890	LLNZ_04580	NCDO2118_RS08810	L0227	core	Pyrimidine operon regulatory protein	Core	F	x	x	x	x
llmg_0912	LLNZ_04685	NCDO2118_RS08425	L0237	core	UDP-N-acetylmuramoylalanine--D-glutamate ligase	Core	M	x	x	x	x
llmg_0913	LLNZ_04690	NCDO2118_RS08420	L0238	core	UDP-N-acetylglucosamine--N-acetylmuramyl- (pentapeptide)	Core	M	x	x	x	x
llmg_2241	LLNZ_11555	NCDO2118_RS10925	L0242	core	Transcriptional regulator	Core	H	x	x	x	x
llmg_0172	LLNZ_00910	NCDO2118_RS00800	L0243	core	Transcriptional regulator	Core	K	x	x	x	x
llmg_2512	LLNZ_12975	NCDO2118_RS12325	L0245	core	Crp/Fnr family transcriptional regulator	Core	K	x	x	x	x
llmg_1818	LLNZ_09360	NCDO2118_RS03910	L0248	core	MarR family transcriptional regulator	Core	K	x	x	x	x
llmg_1691	LLNZ_08725	NCDO2118_RS04475	L0255	core	exodeoxyribonuclease 7 small subunit	Core	L	x	x	x	x
llmg_2008	LLNZ_10350	NCDO2118_RS10080	L0256	core	excinuclease ABC subunit A	Core	L	x	x	x	x
llmg_0534	LLNZ_02755	NCDO2118_RS02835	L0257	core	Excinuclease ABC subunit B	Core	L	x	x	x	x
llmg_1718	LLNZ_08860	NCDO2118_RS04370	L0258	core	Excinuclease ABC subunit C	Core	L	x	x	x	x
llmg_0374	LLNZ_01955	NCDO2118_RS02085	L0260	core	RecA protein	Core	L	x	x	x	x

llmg_0359	LLNZ_01880	NCDO2118_RS02015	L0265	core	RecR protein	Core	L	x	x	x	x
llmg_2488	LLNZ_12855	NCDO2118_RS12210	L0266	core	Holliday junction DNA helicase RuvA	Core	L	x	x	x	x
llmg_2487	LLNZ_12850	NCDO2118_RS12205	L0267	core	Holliday junction DNA helicase RuvB	Core	L	x	x	x	x
llmg_2425	LLNZ_12535	NCDO2118_RS11730	L0270	core	DNA polymerase I	Core	L	x	x	x	x
llmg_2502	LLNZ_12920	NCDO2118_RS12275	L0272	core	Molecular chaperone DnaJ	Core	O	x	x	x	x
llmg_1575	LLNZ_08110	NCDO2118_RS05310	L0273	core	Protein GrpE	Core	F	x	x	x	x
llmg_0001	LLNZ_00005	NCDO2118_RS00005	L0274	core	Chromosomal replication initiator protein DnaA	Core	L	x	x	x	x
llmg_0002	LLNZ_00010	NCDO2118_RS00010	L0275	core	DNA polymerase III subunit beta	Core	L	x	x	x	x
llmg_2489	LLNZ_12860	NCDO2118_RS12215	L0276	core	DNA mismatch repair protein MutL	Core	L	x	x	x	x
llmg_2478	LLNZ_12805	NCDO2118_RS12010	L0279	core	DNA polymerase III subunit gamma/tau	Core	L	x	x	x	x
llmg_1451	LLNZ_07480	NCDO2118_RS05800	L0282	core	DNA gyrase subunit A	Core	L	x	x	x	x
llmg_1631	LLNZ_08395	NCDO2118_RS04755	L0283	core	DNA gyrase subunit B	Core	L	x	x	x	x
llmg_2021	LLNZ_10415	NCDO2118_RS10145	L0284	core	3'-5' exonuclease	Core	L	x	x	x	x
llmg_1814	LLNZ_09340	NCDO2118_RS03930	L0286	core	Replicative DNA helicase	Core	L	x	x	x	x
llmg_1445	LLNZ_07450	NCDO2118_RS05835	L0287	core	ATP-dependent DNA helicase	Core	L	x	x	x	x
llmg_1534	LLNZ_07890	NCDO2118_RS05460	L0291	core	DNA topoisomerase 4 subunit A	Core	L	x	x	x	x
llmg_0778	LLNZ_04040	NCDO2118_RS09035	L0293	core	DNA mismatch repair protein MutS	Core	L	x	x	x	x
llmg_1543	LLNZ_07930	NCDO2118_RS05420	L0294	core	Ribonucleotide-diphosphate reductase subunit alpha	Core	F	x	x	x	x
llmg_1544	LLNZ_07935	NCDO2118_RS05415	L0295	core	Ribonucleoside-diphosphate reductase	Core	F	x	x	x	x
llmg_1272	LLNZ_06565	NCDO2118_RS06460	L0298	core	DNA topoisomerase I	Core	L	x	x	x	x
llmg_2343	LLNZ_12110	NCDO2118_RS11315	L0303	core	DNA repair protein radA	Core	L	x	x	x	x
llmg_0444	LLNZ_02300	NCDO2118_RS02395	L0304	core	DNA ligase	Core	L	x	x	x	x
llmg_1176	LLNZ_06055	NCDO2118_RS06870	L0320	core	Ribonuclease HII	Core	L	x	x	x	x
llmg_1275	LLNZ_06580	NCDO2118_RS06445	L0321	core	Alpha-acetolactate decarboxylase	Core	Q	x	x	x	x
llmg_2044	LLNZ_10530	NCDO2118_RS10260	L0325	core	Polynucleotide phosphorylase	Core	J	x	x	x	x
llmg_1431	LLNZ_07375	NCDO2118_RS05865	L0328	core	tRNA pseudouridine synthase B	Core	F	x	x	x	x
llmg_2313	LLNZ_11945	NCDO2118_RS11175	L0329	core	Arginine deiminase	Core	E	x	x	x	x
llmg_2063	LLNZ_10620	NCDO2118_RS10345	L0330	core	rRNA methyltransferase	Core	R	x	x	x	x
llmg_0124	LLNZ_00630	NCDO2118_RS00445	L0334	core	Preprotein translocase subunit SecA	Core	U	x	x	x	x
llmg_0519	LLNZ_02680	NCDO2118_RS02690	L0336	core	tig trigger factor	Core	D	x	x	x	x
llmg_2550	LLNZ_13175	NCDO2118_RS12490	L0337	core	Signal peptidase	Core	U	x	x	x	x
llmg_0369	LLNZ_01935	NCDO2118_RS02065	L0339	core	RNA helicase	Core	L	x	x	x	x
llmg_0433	LLNZ_02255	NCDO2118_RS02345	L0340	core	DEAD/DEAH box helicase	Core	L	x	x	x	x
llmg_2217	LLNZ_11435	NCDO2118_RS10805	L0342	core	Histidine--tRNA ligase	Core	J	x	x	x	x
llmg_1906	LLNZ_09815	NCDO2118_RS09495	L0343	core	Alanine--tRNA ligase	Core	J	x	x	x	x
llmg_2314	LLNZ_11950	NCDO2118_RS11185	L0344	core	Arginine--tRNA ligase	Core	J	x	x	x	x
llmg_2017	LLNZ_10395	NCDO2118_RS10125	L0345	core	Asparaginyl-tRNA synthase	Core	J	x	x	x	x
llmg_2215	LLNZ_11425	NCDO2118_RS10795	L0346	core	Aspartyl-tRNA synthetase	Core	J	x	x	x	x
llmg_0389	LLNZ_02030	NCDO2118_RS02160	L0347	core	Lysyl-tRNA synthetase	Core	J	x	x	x	x
llmg_2040	LLNZ_10510	NCDO2118_RS10240	L0348	core	Cysteinyl-tRNA synthetase	Core	J	x	x	x	x
llmg_2053	LLNZ_10575	NCDO2118_RS10300	L0350	core	Isoleucyl-tRNA synthetase	Core	J	x	x	x	x
llmg_2455	LLNZ_12695	NCDO2118_RS11890	L0351	core	Valyl-tRNA synthetase	Core	J	x	x	x	x
llmg_1741	LLNZ_08995	NCDO2118_RS04275	L0352	core	Leucine--tRNA ligase	Core	J	x	x	x	x
llmg_1764	LLNZ_09110	NCDO2118_RS04165	L0353	core	Methionyl-tRNA synthetase	Core	J	x	x	x	x
llmg_2196	LLNZ_11335	NCDO2118_RS10695	L0354	core	Phenylalanine--tRNA ligase subunit alpha	Core	J	x	x	x	x
llmg_2195	LLNZ_11330	NCDO2118_RS10690	L0355	core	Phenylalanine--tRNA ligase subunit beta	Core	J	x	x	x	x
llmg_2412	LLNZ_12470	NCDO2118_RS11665	L0356	core	Prolyl-tRNA synthetase	Core	J	x	x	x	x
llmg_2169	LLNZ_11200	NCDO2118_RS10595	L0357	core	Threonine--tRNA ligase	Core	J	x	x	x	x
llmg_0079	LLNZ_00390	NCDO2118_RS00250	L0358	core	Tryptophan--tRNA ligase	Core	J	x	x	x	x
llmg_0401	LLNZ_02095	NCDO2118_RS02220	L0359	core	Tyrosyl-tRNA synthetase	Core	J	x	x	x	x
llmg_1477	LLNZ_07605	NCDO2118_RS05720	L0360	core	Glycine--tRNA ligase subunit beta	Core	J	x	x	x	x
llmg_0164	LLNZ_00860	NCDO2118_RS00755	L0361	core	Tgt queuine tRNA-ribosyltransferase	Core	G	x	x	x	x
llmg_2147	LLNZ_11075	NCDO2118_RS10450	L0362	core	Methionyl-tRNA formyltransferase	Core	J	x	x	x	x
llmg_1882	LLNZ_09695	NCDO2118_RS03655	L0363	core	Ribosomal RNA small subunit methyltransferase A	Core	J	x	x	x	x
llmg_2358	LLNZ_12190	NCDO2118_RS11400	L0366	core	Translation initiation factor IF-1	Core	J	x	x	x	x
llmg_2284	LLNZ_11810	NCDO2118_RS11040	L0367	core	Ribosome-recycling factor	Core	J	x	x	x	x
llmg_2556	LLNZ_13210	NCDO2118_RS12500	L0368	core	Elongation factor G	Core	J	x	x	x	x
llmg_0368	LLNZ_01930	NCDO2118_RS02060	L0369	core	peptide chain release factor 3	Core	S	x	x	x	x
llmg_2292	LLNZ_11845	NCDO2118_RS11075	L0370	core	GTP-binding protein	Core	F	x	x	x	x
llmg_2050	LLNZ_10560	NCDO2118_RS10290	L0371	core	Elongation factor Tu	Core	J	x	x	x	x
llmg_1792	LLNZ_09235	NCDO2118_RS04040	L0372	core	Translation initiation factor IF-2	Core	J	x	x	x	x
llmg_0557	LLNZ_02860	NCDO2118_RS03000	L0373	core	peptide chain release factor 1	Core	S	x	x	x	x
llmg_1547	LLNZ_07950	NCDO2118_RS05400	L0374	core	Peptide chain release factor 2	Core	J	x	x	x	x
llmg_1791	LLNZ_09230	NCDO2118_RS04045	L0375	core	Ribosome-binding factor A	Core	J	x	x	x	x
llmg_2429	LLNZ_12555	NCDO2118_RS11775	L0376	core	Elongation factor Ts	Core	J	x	x	x	x
llmg_1724	LLNZ_08905	NCDO2118_RS04355	L0378	core	30S ribosomal protein S1	Core	J	x	x	x	x
llmg_2430	LLNZ_12560	NCDO2118_RS11780	L0379	core	30S ribosomal protein S2	Core	J	x	x	x	x
llmg_2377	LLNZ_12280	NCDO2118_RS11485	L0380	core	30S ribosomal protein S3	Core	J	x	x	x	x
llmg_0296	LLNZ_01555	NCDO2118_RS01710	L0381	core	30S ribosomal protein S4	Core	R	x	x	x	x
llmg_2364	LLNZ_12215	NCDO2118_RS11425	L0382	core	30S ribosomal protein S5	Core	J	x	x	x	x
llmg_2475	LLNZ_12790	NCDO2118_RS11995	L0383	core	30S ribosomal protein S6	Core	J	x	x	x	x
llmg_2557	LLNZ_13215	NCDO2118_RS12505	L0384	core	30S ribosomal protein S7	Core	J	x	x	x	x
llmg_2367	LLNZ_12230	NCDO2118_RS11440	L0385	core	30S ribosomal protein S8	Core	J	x	x	x	x
llmg_2545	LLNZ_13150	NCDO2118_RS12465	L0386	core	30S ribosomal protein S9	Core	J	x	x	x	x
llmg_2384	LLNZ_12315	NCDO2118_RS11520	L0387	core	30S ribosomal protein S10	Core	J	x	x	x	x
llmg_2355	LLNZ_12175	NCDO2118_RS11385	L0388	core	30S ribosomal protein S11	Core	J	x	x	x	x
llmg_2558	LLNZ_13220	NCDO2118_RS12510	L0389	core	30S ribosomal protein S12	Core	J	x	x	x	x
llmg_2356	LLNZ_12180	NCDO2118_RS11390	L0390	core	30S ribosomal protein S13	Core	J	x	x	x	x
llmg_2078	LLNZ_10690	NCDO2118_RS10410	L0392	core	30S ribosomal protein S15	Core	J	x	x	x	x
llmg_0932	LLNZ_04795	NCDO2118_RS08215	L0393	core	30S ribosomal protein S16	Core	J	x	x	x	x
llmg_2374	LLNZ_12265	NCDO2118_RS11470	L0394	core	30S ribosomal protein S17	Core	J	x	x	x	x
llmg_2473	LLNZ_12780	NCDO2118_RS11985	L0395	core	30S ribosomal protein S18	Core	J	x	x	x	x
llmg_2379	LLNZ_12290	NCDO2118_RS11495	L0396	core	30S ribosomal protein S19	Core	J	x	x	x	x
llmg_1921	LLNZ_09890	NCDO2118_RS09570	L0397	core	30S ribosomal protein S20	Core	J	x	x	x	x
llmg_2276	LLNZ_11770	NCDO2118_RS11000	L0399	core	50S ribosomal protein L1	Core	J	x	x	x	x
llmg_2380	LLNZ_12295	NCDO2118_RS11500	L0400	core	50S ribosomal protein L2	Core	J	x	x	x	x
llmg_2383	LLNZ_12310	NCDO2118_RS11515	L0401	core	50S ribosomal protein L3	Core	J	x	x	x	x
llmg_2382	LLNZ_12305	NCDO2118_RS11510	L0402	core	50S ribosomal protein L4	Core	J	x	x	x	x
llmg_2371	LLNZ_12250	NCDO2118_RS11455	L0403	core	50S ribosomal protein L5	Core	J	x	x	x	x
llmg_2366	LLNZ_12225	NCDO2118_RS11435	L0404	core	50S ribosomal protein L6	Core	J	x	x	x	x
llmg_1815	LLNZ_09345	NCDO2118_RS03925	L0406	core	50S ribosomal protein L9	Core	J	x	x	x	x
llmg_1207	LLNZ_06215	NCDO2118_RS06750	L0407	core	50S ribosomal protein L10	Core	J	x	x	x	x
llmg_2277	LLNZ_11775	NCDO2118_RS11005	L0408	core	50S ribosomal protein L11	Core	J	x	x	x	x
llmg_2546	LLNZ_13155	NCDO2118_RS12470	L0409	core	50S ribosomal protein L13	Core	J	x	x	x	x
llmg_2373	LLNZ_12260	NCDO2118_RS11465	L0410	core	50S ribosomal protein L14	Core	J	x	x	x	x
llmg_2362	LLNZ_12205	NCDO2118_RS11415	L0411	core	50S ribosomal protein L15	Core	J	x	x	x	x
llmg_2376	LLNZ_12275	NCDO2118_RS11480	L0412	core	50S ribosomal protein L16	Core	J	x	x	x	x
llmg_2353	LLNZ_12165	NCDO2118_RS11375	L0413	core	50S ribosomal protein L17	Core	J	x	x	x	x
llmg_2365	LLNZ_12220	NCDO2118_RS11430	L0414	core	50S ribosomal protein L18	Core	J	x	x	x	x
llmg_1671	LLNZ_08605	NCDO2118_RS04570	L0415	core	50S ribosomal protein L19	Core	J	x	x	x	x
llmg_2029	LLNZ_10460	NCDO2118_RS10195	L0416	core	50S ribosomal protein L20	Core	J	x	x	x	x
llmg_1493	LLNZ_07685	NCDO2118_RS05640	L0417	core	50S ribosomal protein L21	Core	J	x	x	x	x
llmg_2378	LLNZ_12285	NCDO2118_RS11490	L0418	core	50S ribosomal protein L22	Core	J	x	x	x	x
llmg_2381	LLNZ_12300	NCDO2118_RS11505	L0419	core	50S ribosomal protein L23	Core	J	x	x	x	x
llmg_2372	LLNZ_12255	NCDO2118_RS11460	L0420	core	50S ribosomal protein L24	Core	J	x	x	x	x
llmg_1491	LLNZ_07675	NCDO2118_RS05650	L0421	core	50S ribosomal protein L27	Core	J	x	x	x	x
llmg_2375	LLNZ_12270	NCDO2118_RS11475	L0423	core	50S ribosomal protein L29	Core	J	x	x	x	x
llmg_2363	LLNZ_12210	NCDO2118_RS11420	L0424	core	50S ribosomal protein L30	Core	J	x	x	x	x

llmg_0906	LLNZ_04655	NCDO2118_RS08455	L0425	core	50S ribosomal protein L31	Core	J	x	x	x	x
llmg_0175	LLNZ_00920	NCDO2118_RS00810	L0473	core	Glutamyl-tRNA amidotransferase	Core	L	x	x	x	x
llmg_0176	LLNZ_00925	NCDO2118_RS00820	L0474	core	Aspartyl/glutamyl-tRNA(Asn/Gln) amidotransferase subunit B	Core	J	x	x	x	x
llmg_0174	LLNZ_00915	NCDO2118_RS00805	L0475	core	Glutamyl-tRNA(Gln) amidotransferase subunit C	Core	J	x	x	x	x
llmg_0771	LLNZ_04005	NCDO2118_RS09065	L100263	core	RNA methyltransferase	Core	J	x	x	x	x
llmg_0084	LLNZ_00415	NCDO2118_RS00275	L1009	core	Hydrolase	Core	R	x	x	x	x
llmg_0093	LLNZ_00465	NCDO2118_RS00310	L1010	core	Universal stress protein	Core	R	x	x	x	x
llmg_0105	LLNZ_00525	NCDO2118_RS00365	L101209	core	Hypothetical protein	Core	S	x	x	x	x
llmg_1478	LLNZ_07610	NCDO2118_RS05715	L101560	core	glyQ glycine--tRNA ligase subunit alpha	Core	D	x	x	x	x
llmg_2316	LLNZ_11960	NCDO2118_RS11195	L101678	core	UDP-N-acetylmuramate--alanine ligase	Core	J	x	x	x	x
llmg_0318	LLNZ_01660	NCDO2118_RS01805	L101699	core	Thiol peroxidase	Core	S	x	x	x	x
llmg_0106	LLNZ_00530	NCDO2118_RS00370	L101706	core	Heme ABC transporter ATP-binding protein	Core	O	x	x	x	x
llmg_0769	LLNZ_04000	NCDO2118_RS09075	L102051	core	Cell division protein	Core	R	x	x	x	x
llmg_1662	LLNZ_08555	NCDO2118_RS04595	L102093	core	Universal stress protein	Core	C	x	x	x	x
llmg_1204	LLNZ_06195	NCDO2118_RS06760	L102100	core	Transcriptional regulator	Core	T	x	x	x	x
llmg_0496	LLNZ_02565	NCDO2118_RS02580	L102317	core	DNA-binding protein HU	Core	K	x	x	x	x
llmg_0319	LLNZ_01665	NCDO2118_RS01810	L102360	core	Aminopeptidase N	Core	L	x	x	x	x
llmg_2318	LLNZ_11965	NCDO2118_RS11200	L103212	core	Hypothetical protein	Core	T	x	x	x	x
llmg_2319	LLNZ_11970	NCDO2118_RS11205	L104021	core	Helicase	Core	S	x	x	x	x
llmg_1025	LLNZ_05290	NCDO2118_RS07660	L104065	core	Hypothetical protein	Core	L	x	x	x	x
llmg_0110	LLNZ_00550	NCDO2118_RS00385	L104221	core	50S ribosomal protein L11	Core	S	x	x	x	x
llmg_1475	LLNZ_07600	NCDO2118_RS05725	L104681	core	Hypothetical protein	Core	E	x	x	x	x
llmg_1658	LLNZ_08535	NCDO2118_RS04615	L106356	core	Aromatic ring hydroxylating protein	Core	G	x	x	x	x
llmg_1473	LLNZ_07590	NCDO2118_RS05735	L106374	core	Nicotinic acid mononucleotide adenylyltransferase	Core	S	x	x	x	x
llmg_0765	LLNZ_03980	NCDO2118_RS09095	L106425	core	Hypothetical protein	Core	H	x	x	x	x
llmg_1949	LLNZ_10035	NCDO2118_RS09710	L10679	core	F0F1 ATP synthase subunit delta	Core	Q	x	x	x	x
llmg_1472	LLNZ_07580	NCDO2118_RS05745	L107468	core	Nicotinate phosphoribosyltransferase	Core	S	x	x	x	x
llmg_1080	LLNZ_05590	NCDO2118_RS07380	L10754	core	Hypothetical protein	Core	H	x	x	x	x
llmg_1194	LLNZ_06145	NCDO2118_RS06780	L107724	core	Pseudouridine synthase	Core	T	x	x	x	x
llmg_0763	LLNZ_03970	NCDO2118_RS09105	L107797	core	Phosphotransacetylase	Core	C	x	x	x	x
llmg_2045	LLNZ_10535	NCDO2118_RS10265	L107870	core	Hypothetical protein	Core	C	x	x	x	x
llmg_1193	LLNZ_06140	NCDO2118_RS06785	L108430	core	Complex subunit ScpB	Core	R	x	x	x	x
llmg_0323	LLNZ_01700	NCDO2118_RS01830	L108503	core	PadR family transcriptional regulator	Core	L	x	x	x	x
llmg_1471	LLNZ_07575	NCDO2118_RS05750	L108967	core	GCN5 family acetyltransferase	Core	K	x	x	x	x
llmg_1192	LLNZ_06135	NCDO2118_RS06790	L108989	core	Segregation and condensation protein A	Core	Q	x	x	x	x
llmg_0762	LLNZ_03965	NCDO2118_RS09110	L108994	core	Uridine kinase	Core	L	x	x	x	x
llmg_0324	LLNZ_01705	NCDO2118_RS01845	L109162	core	Multidrug ABC transporter ATP-binding protein	Core	F	x	x	x	x
llmg_2046	LLNZ_10540	NCDO2118_RS10270	L109335	core	ribose-phosphate pyrophosphokinase	Core	C	x	x	x	x
llmg_0761	LLNZ_03960	NCDO2118_RS09115	L109882	core	dehydrogenase	Core	L	x	x	x	x
llmg_2520	LLNZ_13010	NCDO2118_RS12360	L110351	core	Hypothetical protein	Core	R	x	x	x	x
llmg_2047	LLNZ_10545	NCDO2118_RS10275	L110467	core	Hypothetical protein	Core	S	x	x	x	x
llmg_0325	LLNZ_01710	NCDO2118_RS01850	L110899	core	Multidrug ABC transporter ATP-binding protein	Core	Q	x	x	x	x
llmg_2048	LLNZ_10550	NCDO2118_RS10280	L111126	core	Cysteine desulfurase	Core	S	x	x	x	x
llmg_1188	LLNZ_06115	NCDO2118_RS06810	L111484	core	NTP phosphatase	Core	U	x	x	x	x
llmg_1950	LLNZ_10040	NCDO2118_RS09715	L11208	core	ATP synthase subunit b	Core	R	x	x	x	x
llmg_2049	LLNZ_10555	NCDO2118_RS10285	L112263	core	Cysteine desulfurase	Core	C	x	x	x	x
llmg_1647	LLNZ_08475	NCDO2118_RS04690	L112952	core	Hydrolase	Core	M	x	x	x	x
llmg_1186	LLNZ_06105	NCDO2118_RS06820	L113060	core	Hypothetical protein	Core	R	x	x	x	x
llmg_0326	LLNZ_01715	NCDO2118_RS01855	L113067	core	UDP-N-acetylglucosamine 1-carboxyvinyltransferase 2	Core	S	x	x	x	x
llmg_1466	LLNZ_07555	NCDO2118_RS05770	L113931	core	Glutamine amidotransferase	Core	O	x	x	x	x
llmg_0119	LLNZ_00600	NCDO2118_RS00430	L114325	core	Acyl-phosphate glycerol 3-phosphate acyltransferase	Core	S	x	x	x	x
llmg_0327	LLNZ_01720	NCDO2118_RS01860	L114363	core	Seryl-tRNA synthetase	Core	I	x	x	x	x
llmg_1465	LLNZ_07550	NCDO2118_RS05775	L114717	core	UDP-N-acetylmuramyl peptide synthase	Core	S	x	x	x	x
llmg_2323	LLNZ_11995	NCDO2118_RS11230	L115113	core	Hypothetical protein	Core	S	x	x	x	x
llmg_0333	LLNZ_01750	NCDO2118_RS01890	L115671	core	Prolyl-tRNA synthetase	Core	C	x	x	x	x
llmg_2540	LLNZ_13120	NCDO2118_RS12390	L117205	core	Hypothetical protein	Core	ER	x	x	x	x
llmg_0335	LLNZ_01760	NCDO2118_RS01900	L117444	core	Lipoprotein	Core	C	x	x	x	x
llmg_0930	LLNZ_04785	NCDO2118_RS08225	L11745	core	3-ketoacyl-CoA thiolase	Core	R	x	x	x	x
llmg_2055	LLNZ_10580	NCDO2118_RS10305	L117685	core	Cell division protein DivIVA	Core	I	x	x	x	x
llmg_1763	LLNZ_09100	NCDO2118_RS04170	L1178	core	Hypothetical protein	Core	KG	x	x	x	x
llmg_2328	LLNZ_12025	NCDO2118_RS11245	L118079	core	Xaa-Pro dipeptidyl-peptidase	Core	P	x	x	x	x
llmg_2431	LLNZ_12565	NCDO2118_RS11785	L11851	core	Hypothetical protein	Core	L	x	x	x	x
llmg_1853	LLNZ_09535	NCDO2118_RS03765	L119891	core	ABC transporter ATP-binding protein	Core	S	x	x	x	x
llmg_0126	LLNZ_00640	NCDO2118_RS00455	L120335	core	Phosphocarrier protein HPr	Core	P	x	x	x	x
llmg_2059	LLNZ_10600	NCDO2118_RS10325	L120355	core	Cell division protein	Core	G	x	x	x	x
llmg_0127	LLNZ_00645	NCDO2118_RS00460	L120628	core	Phosphoenolpyruvate-protein phosphotransferase	Core	K	x	x	x	x
llmg_2330	LLNZ_12035	NCDO2118_RS11255	L121253	core	ABC transporter substrate-binding protein	Core	KT	x	x	x	x
llmg_0341	LLNZ_01785	NCDO2118_RS01920	L121289	core	Methionine import ATP-binding protein	Core	E	x	x	x	x
llmg_0751	LLNZ_03910	NCDO2118_RS09175	L121426	core	Phospho-beta-glucosidase	Core	E	x	x	x	x
llmg_2201	LLNZ_11360	NCDO2118_RS10720	L12179	core	Adenylosuccinate synthetase	Core	S	x	x	x	x
llmg_2331	LLNZ_12040	NCDO2118_RS11260	L122198	core	Dihydrolipoamide dehydrogenase	Core	S	x	x	x	x
llmg_0429	LLNZ_02235	NCDO2118_RS02335	L12227	core	Superoxide dismutase [Mn]	Core	E	x	x	x	x
llmg_0128	LLNZ_00655	NCDO2118_RS00470	L122849	core	nucleoid-associated protein	Core	M	x	x	x	x
llmg_0749	LLNZ_03900	NCDO2118_RS09185	L123365	core	1-deoxy-D-xylulose-5-phosphate synthase	Core	K	x	x	x	x
llmg_2062	LLNZ_10615	NCDO2118_RS10340	L123851	core	DegV domain-containing protein YteA	Core	P	x	x	x	x
llmg_1845	LLNZ_09495	NCDO2118_RS03790	L125485	core	Metal-dependent hydrolase	Core	M	x	x	x	x
llmg_2336	LLNZ_12075	NCDO2118_RS11280	L126998	core	GNAT family acetyltransferase	Core	R	x	x	x	x
llmg_1175	LLNZ_06050	NCDO2118_RS06875	L128951	core	rbgA GTPase	Core	S	x	x	x	x
llmg_0349	LLNZ_01830	NCDO2118_RS01965	L129403	core	Ferrichrome ABC transporter substrate binding protein	Core	V	x	x	x	x
llmg_2069	LLNZ_10650	NCDO2118_RS10375	L130687	core	peptidase PepC protein	Core	QR	x	x	x	x
llmg_2432	LLNZ_12570	NCDO2118_RS11790	L13145	core	Alcohol dehydrogenase	Core	J	x	x	x	x
llmg_1627	LLNZ_08375	NCDO2118_RS04775	L131803	core	Transcriptional regulator	Core	U	x	x	x	x
llmg_0929	LLNZ_04780	NCDO2118_RS08230	L13187	core	hydroxymethylglutaryl-CoA synthase	Core	S	x	x	x	x
llmg_0144	LLNZ_00760	NCDO2118_RS00605	L132712	core	RNA-binding protein	Core	S	x	x	x	x
llmg_2073	LLNZ_10665	NCDO2118_RS10385	L132777	core	5'-methylthioadenosine/S-adenosylhomocysteine nucleosidase	Core	S	x	x	x	x
llmg_0516	LLNZ_02665	NCDO2118_RS02675	L133367	core	inositol monophosphatase	Core	S	x	x	x	x
llmg_2075	LLNZ_10675	NCDO2118_RS10395	L133761	core	ADP-ribose pyrophosphatase	Core	K	x	x	x	x
llmg_0692	LLNZ_03600	NCDO2118_RS11320	L134080	core	Metal transporter	Core	E	x	x	x	x
llmg_0517	LLNZ_02670	NCDO2118_RS02680	L134243	core	UDP-N-acetylglucosamine 1-carboxyvinyltransferase	Core	S	x	x	x	x
llmg_2076	LLNZ_10680	NCDO2118_RS10400	L134450	core	Bifunctional protein GlmU	Core	M	x	x	x	x
llmg_2551	LLNZ_13185	NCDO2118_RS12495	L134453	core	pur operon repressor	Core	M	x	x	x	x
llmg_0356	LLNZ_01865	NCDO2118_RS02000	L135351	core	Alkyl hydroperoxide reductase	Core	K	x	x	x	x
llmg_2077	LLNZ_10685	NCDO2118_RS10405	L135991	core	Pyrroline-5-carboxylate reductase	Core	V	x	x	x	x
llmg_1441	LLNZ_07425	NCDO2118_RS05855	L137630	core	Fumarate reductase	Core	G	x	x	x	x
llmg_1751	LLNZ_09045	NCDO2118_RS04225	L13927	core	Alkaline phosphatase	Core	M	x	x	x	x
llmg_0360	LLNZ_01885	NCDO2118_RS02020	L140690	core	D-alanine--D-alanine ligase	Core	G	x	x	x	x
llmg_2359	LLNZ_12195	NCDO2118_RS11405	L140714	core	Adenylate kinase	Core	M	x	x	x	x
llmg_2080	LLNZ_10700	NCDO2118_RS10420	L140754	core	Protein phosphatase	Core	F	x	x	x	x
llmg_2081	LLNZ_10705	NCDO2118_RS10425	L141260	core	16S rRNA methyltransferase	Core	M	x	x	x	x
llmg_0150	LLNZ_00790	NCDO2118_RS00620	L141485	core	Hypothetical protein	Core	J	x	x	x	x
llmg_1825	LLNZ_09390	NCDO2118_RS03875	L141547	core	Hypothetical protein	Core	S	x	x	x	x
llmg_0151	LLNZ_00795	NCDO2118_RS00625	L141748	core	Holliday junction DNA helicase	Core	E	x	x	x	x
llmg_0361	LLNZ_01890	NCDO2118_RS02025	L141766	core	UDP-N-acetylmuramoyl-tripeptide--D-alanyl-D- alanine ligase	Core	L	x	x	x	x
llmg_0732	LLNZ_03810	NCDO2118_RS09355	L143791	core	Cytidylate kinase	Core	S	x	x	x	x
llmg_1427	LLNZ_07355	NCDO2118_RS05885	L144334	core	N-acetylmuramic acid 6-phosphate etherase	Core	S	x	x	x	x
llmg_0727	LLNZ_03785	NCDO2118_RS09380	L147466	core	Mannose-specific PTS system component IID	Core	K	x	x	x	x
llmg_0726	LLNZ_03780	NCDO2118_RS09385	L148513	core	Regulator	Core	G	x	x	x	x
llmg_0724	LLNZ_03770	NCDO2118_RS09395	L149781	core	Regulator	Core	S	x	x	x	x

llmg_0367	LLNZ_01925	NCDO2118_RS02055	L150031	core	Peptide ABC transporter substrate-binding protein	Core	S	x	x	x	x
llmg_0722	LLNZ_03765	NCDO2118_RS09400	L150515	core	Serine--tRNA ligase	Core	G	x	x	x	x
llmg_2156	LLNZ_11120	NCDO2118_RS10485	L150593	core	Ribonuclease Y	Core	MI	x	x	x	x
llmg_1136	LLNZ_05865	NCDO2118_RS07110	L150744	core	Manganese ABC transporter ATP-binding protein	Core	J	x	x	x	x
llmg_1762	LLNZ_09095	NCDO2118_RS04175	L1530	core	3-ketoacyl-ACP reductase	Core	G	x	x	x	x
llmg_2160	LLNZ_11140	NCDO2118_RS10495	L153408	core	S-adenosylmethionine synthetase	Core	F	x	x	x	x
llmg_1895	LLNZ_09760	NCDO2118_RS09450	L153665	core	PhoU family transcriptional regulator	Core	H	x	x	x	x
llmg_1896	LLNZ_09765	NCDO2118_RS09455	L154481	core	Phosphate import ATP-binding protein PstB 1	Core	S	x	x	x	x
llmg_0532	LLNZ_02745	NCDO2118_RS02825	L154885	core	peptide deformylase	Core	K	x	x	x	x
llmg_1601	LLNZ_08235	NCDO2118_RS04880	L154925	core	phosphopentomutase	Core	K	x	x	x	x
llmg_1813	LLNZ_09335	NCDO2118_RS03935	L155040	core	Hypothetical protein	Core	G	x	x	x	x
llmg_1897	LLNZ_09770	NCDO2118_RS09460	L155408	core	Phosphate import ATP-binding protein PstB 2	Core	R	x	x	x	x
llmg_1415	LLNZ_07295	NCDO2118_RS05940	L155662	core	Acyl-ACP thioesterase	Core	LR	x	x	x	x
llmg_1811	LLNZ_09325	NCDO2118_RS03945	L156302	core	tRNA (guanine-N(7)-)-methyltransferase	Core	P	x	x	x	x
llmg_1414	LLNZ_07290	NCDO2118_RS05945	L156445	core	haloacid dehalogenase	Core	R	x	x	x	x
llmg_1599	LLNZ_08225	NCDO2118_RS04890	L156559	core	Purine nucleoside phosphorylase	Core	M	x	x	x	x
llmg_1810	LLNZ_09320	NCDO2118_RS03950	L157023	core	NrdR family transcriptional regulator	Core	S	x	x	x	x
llmg_1131	LLNZ_05840	NCDO2118_RS07135	L157055	core	2-dehydropantoate 2-reductase	Core	K	x	x	x	x
llmg_2388	LLNZ_12335	NCDO2118_RS11540	L157316	core	Transcription termination/antitermination protein NusG	Core	S	x	x	x	x
llmg_2164	LLNZ_11165	NCDO2118_RS10520	L157995	core	Hypothetical protein	Core	L	x	x	x	x
llmg_1412	LLNZ_07280	NCDO2118_RS05955	L158186	core	GMP reductase	Core	U	x	x	x	x
llmg_1900	LLNZ_09785	NCDO2118_RS09475	L158188	core	Phosphate ABC transporter substrate-binding protein	Core	J	x	x	x	x
llmg_1597	LLNZ_08215	NCDO2118_RS05205	L158463	core	Hypothetical protein	Core	E	x	x	x	x
llmg_1596	LLNZ_08210	NCDO2118_RS05210	L158972	core	Hypothetical protein	Core	S	x	x	x	x
llmg_1595	LLNZ_08205	NCDO2118_RS05215	L159505	core	formate--tetrahydrofolate ligase	Core	S	x	x	x	x
llmg_0538	LLNZ_02770	NCDO2118_RS02850	L160425	core	3-hydroxyacyl-ACP dehydratase	Core	E	x	x	x	x
llmg_2392	LLNZ_12355	NCDO2118_RS11560	L160485	core	penicillin-binding protein 2A	Core	F	x	x	x	x
llmg_0170	LLNZ_00900	NCDO2118_RS00790	L160937	core	Glycosyltransferase	Core	S	x	x	x	x
llmg_0539	LLNZ_02775	NCDO2118_RS02855	L161132	core	enoyl-ACP reductase	Core	MG	x	x	x	x
llmg_1750	LLNZ_09040	NCDO2118_RS04230	L16147	core	Hypothetical protein	Core	S	x	x	x	x
llmg_1125	LLNZ_05815	NCDO2118_RS07155	L162597	core	fibronectin-binding protein	Core	Q	x	x	x	x
llmg_0171	LLNZ_00905	NCDO2118_RS00795	L162604	core	Aminotransferase AlaT	Core	R	x	x	x	x
llmg_1340	LLNZ_06930	NCDO2118_RS05990	L163602	core	ATP-dependent Clp protease ATP-binding subunit ClpX	Core	S	x	x	x	x
llmg_1590	LLNZ_08185	NCDO2118_RS05235	L164012	core	ABC-type amino acid transport system, ATP-binding protein	Core	S	x	x	x	x
llmg_0540	LLNZ_02780	NCDO2118_RS02875	L164312	core	Protein translocase component YidC	Core	R	x	x	x	x
llmg_0380	LLNZ_01985	NCDO2118_RS02115	L164461	core	Adenylate cyclase	Core	S	x	x	x	x
llmg_1907	LLNZ_09820	NCDO2118_RS09500	L164604	core	Foldase	Core	S	x	x	x	x
llmg_1942	LLNZ_10000	NCDO2118_RS09670	L165	core	ABC-type amino acid transport system permease	Core	J	x	x	x	x
llmg_0926	LLNZ_04765	NCDO2118_RS08355	L16514	core	S-adenosylmethionine tRNA ribosyltransferase	Core	E	x	x	x	x
llmg_1123	LLNZ_05805	NCDO2118_RS07165	L165279	core	ABC transporter ATP binding protein	Core	F	x	x	x	x
llmg_0986	LLNZ_05075	NCDO2118_RS07985	L166407	core	Chaperone protein ClpB	Core	E	x	x	x	x
llmg_1122	LLNZ_05800	NCDO2118_RS07170	L166804	core	Branched-chain amino acid ABC transporter permease	Core	P	x	x	x	x
llmg_0543	LLNZ_02795	NCDO2118_RS02890	L166912	core	phosphopantothenoylcysteine decarboxylase	Core	G	x	x	x	x
llmg_2400	LLNZ_12405	NCDO2118_RS11595	L167426	core	Zinc ABC transporter substrate-binding protein	Core	S	x	x	x	x
llmg_0593	LLNZ_03045	NCDO2118_RS03230	L16806	core	Protease	Core	LR	x	x	x	x
llmg_2401	LLNZ_12410	NCDO2118_RS11600	L168265	core	MarR family transcriptional regulator	Core	R	x	x	x	x
llmg_1582	LLNZ_08145	NCDO2118_RS05275	L172073	core	Hypothetical protein	Core	H	x	x	x	x
llmg_1581	LLNZ_08140	NCDO2118_RS05280	L172471	core	Hypothetical protein	Core	S	x	x	x	x
llmg_0180	LLNZ_00940	NCDO2118_RS00835	L172505	core	Cold-shock protein	Core	S	x	x	x	x
llmg_2170	LLNZ_11205	NCDO2118_RS10600	L173045	core	Ribonucleotide reductase	Core	H	x	x	x	x
llmg_1117	LLNZ_05775	NCDO2118_RS07200	L173068	core	N-acetylglucosamine-6-phosphate deacetylase	Core	S	x	x	x	x
llmg_1797	LLNZ_09255	NCDO2118_RS04020	L173151	core	Ribosome maturation protein	Core	M	x	x	x	x
llmg_1914	LLNZ_09855	NCDO2118_RS09535	L173313	core	haloacid dehalogenase	Core	S	x	x	x	x
llmg_1796	LLNZ_09250	NCDO2118_RS04025	L173813	core	Transcription termination factor NusA	Core	G	x	x	x	x
llmg_1116	LLNZ_05770	NCDO2118_RS07205	L174389	core	Tellurite resistance protein	Core	C	x	x	x	x
llmg_2172	LLNZ_11215	NCDO2118_RS10610	L174788	core	Hypothetical protein	Core	S	x	x	x	x
llmg_0185	LLNZ_00965	NCDO2118_RS00855	L174946	core	Acetyltransferase	Core	C	x	x	x	x
llmg_1916	LLNZ_09865	NCDO2118_RS09545	L175101	core	Amino acid dehydrogenase	Core	Q	x	x	x	x
llmg_1328	LLNZ_06870	NCDO2118_RS06050	L175357	core	Spermidine/putrescine import ATP-binding protein PotA	Core	M	x	x	x	x
llmg_1793	LLNZ_09240	NCDO2118_RS04035	L175450	core	Hypothetical protein	Core	E	x	x	x	x
llmg_2176	LLNZ_11240	NCDO2118_RS10615	L175538	core	Uracil phosphoribosyltransferase	Core	S	x	x	x	x
llmg_0974	LLNZ_05005	NCDO2118_RS08020	L177031	core	Phosphoribosylformylglycinamidine synthase subunit PurS	Core	E	x	x	x	x
llmg_1113	LLNZ_05755	NCDO2118_RS07220	L177590	core	UTP--glucose-1-phosphate uridylyltransferase	Core	G	x	x	x	x
llmg_1574	LLNZ_08105	NCDO2118_RS05315	L178206	core	Molecular chaperone DnaK	Core	S	x	x	x	x
llmg_0553	LLNZ_02840	NCDO2118_RS02955	L178303	core	Hypothetical protein	Core	O	x	x	x	x
llmg_1325	LLNZ_06855	NCDO2118_RS06065	L178329	core	ABC transporter spermidine/putrescine substrate-binding protein	Core	S	x	x	x	x
llmg_1789	LLNZ_09225	NCDO2118_RS04050	L179409	core	Mannose-6-phosphate isomerase	Core	S	x	x	x	x
llmg_1110	LLNZ_05740	NCDO2118_RS07235	L179531	core	heptaprenyl diphosphate synthase subunit II	Core	G	x	x	x	x
llmg_1922	LLNZ_09895	NCDO2118_RS09575	L180415	core	Exodeoxyribonuclease V subunit alpha	Core	S	x	x	x	x
llmg_0191	LLNZ_00995	NCDO2118_RS00885	L181168	core	deoxyuridine 5'-triphosphate nucleotidohydrolase	Core	K	x	x	x	x
llmg_2183	LLNZ_11270	NCDO2118_RS10640	L181238	core	Hypothetical protein	Core	F	x	x	x	x
llmg_2413	LLNZ_12475	NCDO2118_RS11670	L181494	core	Metalloprotease	Core	S	x	x	x	x
llmg_0192	LLNZ_01000	NCDO2118_RS00900	L181858	core	5'-nucleotidase	Core	S	x	x	x	x
llmg_1106	LLNZ_05720	NCDO2118_RS07255	L182555	core	Dihydroorotase dehydrogenase	Core	R	x	x	x	x
llmg_0964	LLNZ_04950	NCDO2118_RS08065	L182559	core	Thymidylate synthase	Core	F	x	x	x	x
llmg_2185	LLNZ_11280	NCDO2118_RS10650	L183012	core	Aldose 1-epimerase	Core	G	x	x	x	x
llmg_0395	LLNZ_02060	NCDO2118_RS02190	L183112	core	Thiamine biosynthesis protein ThiI	Core	G	x	x	x	x
llmg_2415	LLNZ_12485	NCDO2118_RS11680	L183602	core	UDP pyrophosphate synthase	Core	F	x	x	x	x
llmg_0194	LLNZ_01010	NCDO2118_RS00910	L184159	core	23S rRNA (adenine(2503)-C(2))-methyltransferase RlmN	Core	S	x	x	x	x
llmg_2187	LLNZ_11290	NCDO2118_RS10660	L185224	core	RmuC family endonuclease	Core	L	x	x	x	x
llmg_0195	LLNZ_01015	NCDO2118_RS00915	L185517	core	Nitroreductase	Core	S	x	x	x	x
llmg_0397	LLNZ_02075	NCDO2118_RS02200	L186107	core	FMN-dependent NADH-azoreductase 2	Core	I	x	x	x	x
llmg_0196	LLNZ_01020	NCDO2118_RS00920	L186258	core	Prenyl transferase	Core	E	x	x	x	x
llmg_1103	LLNZ_05705	NCDO2118_RS07310	L186490	core	Virion core protein	Core	H	x	x	x	x
llmg_0604	LLNZ_03100	NCDO2118_RS03250	L18686	core	Ribonuclease Z	Core	S	x	x	x	x
llmg_2419	LLNZ_12505	NCDO2118_RS11700	L187771	core	Serine protease	Core	C	x	x	x	x
llmg_1557	LLNZ_08000	NCDO2118_RS05350	L188550	core	ATPase P	Core	J	x	x	x	x
llmg_0437	LLNZ_02270	NCDO2118_RS02360	L18872	core	PTS sugar transporter	Core	S	x	x	x	x
llmg_2420	LLNZ_12510	NCDO2118_RS11705	L189090	core	Gycosyltransferase	Core	S	x	x	x	x
llmg_1556	LLNZ_07995	NCDO2118_RS05355	L189428	core	Hypothetical protein	Core	KR	x	x	x	x
llmg_0562	LLNZ_02885	NCDO2118_RS03025	L189883	core	Hypothetical protein	Core	J	x	x	x	x
llmg_2421	LLNZ_12515	NCDO2118_RS11710	L190226	core	1,2-diacylglycerol 3-glucosyltransferase	Core	P	x	x	x	x
llmg_1317	LLNZ_06805	NCDO2118_RS06115	L191486	core	N-acetylmannosamine-6-phosphate 2-epimerase	Core	S	x	x	x	x
llmg_2422	LLNZ_12520	NCDO2118_RS11715	L191704	core	Hypothetical protein	Core	G	x	x	x	x
llmg_0952	LLNZ_04890	NCDO2118_RS08120	L192589	core	Dihydroorotase dehydrogenase	Core	M	x	x	x	x
llmg_0438	LLNZ_02275	NCDO2118_RS02365	L19292	core	PTS cellobiose transporter subunit IIA	Core	J	x	x	x	x
llmg_1773	LLNZ_09155	NCDO2118_RS04120	L193031	core	Hypothetical protein	Core	U	x	x	x	x
llmg_2424	LLNZ_12530	NCDO2118_RS11725	L193121	core	Hypothetical protein	Core	S	x	x	x	x
llmg_0202	LLNZ_01055	NCDO2118_RS00955	L193593	core	dihydroxyacetone kinase	Core	S	x	x	x	x
llmg_1315	LLNZ_06795	NCDO2118_RS06125	L193873	core	RNA methyltransferase	Core	Q	x	x	x	x
llmg_0403	LLNZ_02105	NCDO2118_RS02230	L193909	core	Glutamyl aminopeptidase	Core	R	x	x	x	x
llmg_0203	LLNZ_01060	NCDO2118_RS00960	L195257	core	Hypothetical protein	Core	R	x	x	x	x
llmg_0406	LLNZ_02115	NCDO2118_RS02240	L195348	core	Thioredoxin H-type	Core	P	x	x	x	x
llmg_0948	LLNZ_04870	NCDO2118_RS08140	L195415	core	Antibiotic ABC transporter ATP-binding protein	Core	S	x	x	x	x
llmg_0407	LLNZ_02120	NCDO2118_RS02245	L195751	core	tRNA-binding protein	Core	S	x	x	x	x
llmg_1768	LLNZ_09130	NCDO2118_RS04145	L196017	core	Arsenate reductase	Core	J	x	x	x	x
llmg_1550	LLNZ_07965	NCDO2118_RS05390	L196206	core	Hypothetical protein	Core	R	x	x	x	x
llmg_0205	LLNZ_01070	NCDO2118_RS00970	L196216	core	Hypothetical protein	Core	S	x	x	x	x

llmg_0408	LLNZ_02125	NCDO2118_RS02250	L196579	core	NADH oxidase	Core	S	x	x	x	x
llmg_0206	LLNZ_01075	NCDO2118_RS00975	L197041	core	Glucose-1-phosphate thymidylyltransferase	Core	S	x	x	x	x
llmg_0574	LLNZ_02945	NCDO2118_RS03070	L197697	core	flavodoxin	Core	M	x	x	x	x
llmg_1089	LLNZ_05635	NCDO2118_RS07330	L198033	core	Carbamoyl phosphate synthase large subunit	Core	R	x	x	x	x
llmg_1765	LLNZ_09115	NCDO2118_RS04160	L198056	core	Exodeoxyribonuclease	Core	F	x	x	x	x
llmg_0207	LLNZ_01080	NCDO2118_RS00985	L198323	core	dTDP-4-dehydrorhamnose 3,5-epimerase	Core	L	x	x	x	x
llmg_0410	LLNZ_02135	NCDO2118_RS02260	L198515	core	Molecular chaperone GroES	Core	C	x	x	x	x
llmg_0411	LLNZ_02140	NCDO2118_RS02265	L198893	core	Molecular chaperone GroEL	Core	S	x	x	x	x
llmg_0209	LLNZ_01090	NCDO2118_RS00995	L199221	core	dTDP-glucose 4,6-dehydratase	Core	S	x	x	x	x
llmg_0944	LLNZ_04855	NCDO2118_RS08155	L199664	core	Hypothetical protein	Core	R	x	x	x	x
llmg_0229	LLNZ_01185	NCDO2118_RS01095	L20397	core	Family 25 glycosyl hydrolase	Core	S	x	x	x	x
llmg_2442	LLNZ_12625	NCDO2118_RS11830	L20937	core	peptidase S16	Core	G	x	x	x	x
llmg_1309	LLNZ_06770	NCDO2118_RS06150	L210	core	Acetolactate synthase	Core	S	x	x	x	x
llmg_0230	LLNZ_01190	NCDO2118_RS01100	L21264	core	inosine-5-monophosphate dehydrogenase	Core	E	x	x	x	x
llmg_1746	LLNZ_09020	NCDO2118_RS04250	L22687	core	Haloacid dehalogenase	Core	L	x	x	x	x
llmg_0607	LLNZ_03115	NCDO2118_RS03265	L22735	core	Adenine phosphoribosyltransferase	Core	S	x	x	x	x
llmg_0231	LLNZ_01195	NCDO2118_RS01105	L22900	core	GTPase	Core	F	x	x	x	x
llmg_1943	LLNZ_10005	NCDO2118_RS09675	L2385	core	Glutamine ABC transporter ATP-binding protein	Core	S	x	x	x	x
llmg_0609	LLNZ_03125	NCDO2118_RS03275	L24228	core	aminodeoxychorismate lyase	Core	S	x	x	x	x
llmg_0020	LLNZ_00095	NCDO2118_RS00105	L25115	core	Hypoxanthine-guanine phosphoribosyltransferase	Core	S	x	x	x	x
llmg_1743	LLNZ_09005	NCDO2118_RS04265	L25614	core	Ribose-phosphate pyrophosphokinase 1	Core	S	x	x	x	x
llmg_0235	LLNZ_01215	NCDO2118_RS01120	L26054	core	Hypothetical protein	Core	M	x	x	x	x
llmg_0237	LLNZ_01225	NCDO2118_RS01130	L26998	core	Hypothetical protein	Core	S	x	x	x	x
llmg_0446	LLNZ_02310	NCDO2118_RS02405	L27865	core	Sugar ABC transporter ATP binding protein	Core	R	x	x	x	x
llmg_0239	LLNZ_01235	NCDO2118_RS01140	L28204	core	Ribosome silencing factor	Core	G	x	x	x	x
llmg_1086	LLNZ_05620	NCDO2118_RS07345	L2866	core	Calcium-transporting ATPase	Core	S	x	x	x	x
llmg_1969	LLNZ_10165	NCDO2118_RS09820	L29491	core	Fe-S cluster assembly protein SufB	Core	R	x	x	x	x
llmg_2452	LLNZ_12680	NCDO2118_RS11875	L29875	core	Hypothetical protein	Core	I	x	x	x	x
llmg_2225	LLNZ_11470	NCDO2118_RS10840	L30285	core	XRE family transcriptional regulator	Core	K	x	x	x	x
llmg_2453	LLNZ_12685	NCDO2118_RS11880	L30878	core	Transcriptional regulator	Core	S	x	x	x	x
llmg_0242	LLNZ_01250	NCDO2118_RS01160	L31280	core	Transcriptional regulator	Core	S	x	x	x	x
llmg_0210	LLNZ_01095	NCDO2118_RS01000	L320	core	dTDP-4-dehydrorhamnose reductase	Core	S	x	x	x	x
llmg_0943	LLNZ_04850	NCDO2118_RS08160	L324	core	Dipeptidase	Core	R	x	x	x	x
llmg_0244	LLNZ_01265	NCDO2118_RS01170	L32653	core	Uracil-DNA glycosylase	Core	S	x	x	x	x
llmg_1759	LLNZ_09085	NCDO2118_RS04185	L3272	core	Hypothetical protein	Core	S	x	x	x	x
llmg_1973	LLNZ_10185	NCDO2118_RS09840	L33412	core	Fe-S cluster assembly protein SufD	Core	S	x	x	x	x
llmg_1516	LLNZ_07795	NCDO2118_RS05535	L33556	core	Glucosamine--fructose-6-phosphate aminotransferase	Core	S	x	x	x	x
llmg_2228	LLNZ_11485	NCDO2118_RS10855	L34138	core	Hypothetical protein	Core	S	x	x	x	x
llmg_1974	LLNZ_10190	NCDO2118_RS09845	L34806	core	Fe-S cluster assembly ATPase SufC	Core	R	x	x	x	x
llmg_0451	LLNZ_02335	NCDO2118_RS02425	L35068	core	Phosphoglucosamine mutase	Core	O	x	x	x	x
llmg_2459	LLNZ_12710	NCDO2118_RS11900	L35545	core	Adose 1-epimerase	Core	G	x	x	x	x
llmg_2464	LLNZ_12735	NCDO2118_RS11920	L37880	core	RNA methyltransferase	Core	X	x	x	x	x
llmg_0907	LLNZ_04660	NCDO2118_RS08450	L39365	core	Hypothetical protein	Core	X	x	x	x	x
llmg_1735	LLNZ_08960	NCDO2118_RS04305	L39857	core	NADH dehydrogenase	Core	S	x	x	x	x
llmg_1734	LLNZ_08955	NCDO2118_RS04310	L41335	core	NADH dehydrogenase	Core	S	x	x	x	x
llmg_0214	LLNZ_01115	NCDO2118_RS01020	L4342	core	ABC transporter ATP-binding protein	Core	F	x	x	x	x
llmg_0894	LLNZ_04595	NCDO2118_RS08795	L43866	core	Carbamoyl phosphate synthase small subunit	Core	E	x	x	x	x
llmg_0893	LLNZ_04590	NCDO2118_RS08800	L45002	core	Aspartate carbamoyltransferase catalytic subunit	Core	R	x	x	x	x
llmg_0054	LLNZ_00270	NCDO2118_RS12040	L47979	core	Hypothetical protein	Core	S	x	x	x	x
llmg_0582	LLNZ_02985	NCDO2118_RS03115	L4822	core	HPr kinase/phosphorylase	Core	S	x	x	x	x
llmg_0702	LLNZ_03650	NCDO2118_RS09935	L49741	core	endopeptidase O	core	P	x	x	x	x
llmg_1541	LLNZ_07920	NCDO2118_RS05430	L5126	core	Glutaredoxin	Core	G	x	x	x	x
llmg_1725	LLNZ_08910	NCDO2118_RS04350	L52034	core	tRNA-specific 2-thiouridylase	Core	U	x	x	x	x
llmg_2243	LLNZ_11565	NCDO2118_RS10935	L52207	core	Protease	Core	S	x	x	x	x
llmg_1989	LLNZ_10265	NCDO2118_RS09910	L53929	core	UDP-N-acetylmuramoylalanyl-D-glutamate--2, 6-diaminopimelate	Core	G	x	x	x	x
llmg_0883	LLNZ_04545	NCDO2118_RS08845	L54040	core	Hypothetical protein	Core	G	x	x	x	x
llmg_2244	LLNZ_11575	NCDO2118_RS10950	L54059	core	Peptidase U32	Core	S	x	x	x	x
llmg_0627	LLNZ_03240	NCDO2118_RS03320	L54546	core	Long-chain acyl-CoA synthetase	Core	S	x	x	x	x
llmg_2470	LLNZ_12765	NCDO2118_RS11970	L54944	core	Transcriptional regulator	Core	F	x	x	x	x
llmg_2246	LLNZ_11585	NCDO2118_RS10960	L55507	core	Methyltransferase	Core	S	x	x	x	x
llmg_0938	LLNZ_04825	NCDO2118_RS08185	L5610	core	Transposase	Core	S	x	x	x	x
llmg_1271	LLNZ_06560	NCDO2118_RS06465	L56416	core	tRNA (uracil-5-)-methyltransferase	Core	P	x	x	x	x
llmg_2269	LLNZ_11735	NCDO2118_RS10965	L56431	core	Copper homeostasis protein	Core	R	x	x	x	x
llmg_0066	LLNZ_00330	NCDO2118_RS00205	L56488	core	aminotransferase A	Core	P	x	x	x	x
llmg_0629	LLNZ_03250	NCDO2118_RS03330	L57408	core	Formate acetyltransferase	Core	S	x	x	x	x
llmg_2271	LLNZ_11745	NCDO2118_RS10975	L58125	core	ABC transporter ATP-binding protein	Core	D	x	x	x	x
llmg_1706	LLNZ_08795	NCDO2118_RS04380	L59867	core	Dipeptidase PepV	Core	C	x	x	x	x
llmg_1994	LLNZ_10290	NCDO2118_RS09935	L60596	core	Peptidase T	Core	V	x	x	x	x
llmg_2274	LLNZ_11760	NCDO2118_RS10990	L61397	core	Hypothetical protein	Core	I	x	x	x	x
llmg_1064	LLNZ_05500	NCDO2118_RS07490	L61620	core	Membrane protein	Core	S	x	x	x	x
llmg_0876	LLNZ_04510	NCDO2118_RS08880	L61727	core	Ribonuclease J	Core	V	x	x	x	x
llmg_1996	LLNZ_10300	NCDO2118_RS09945	L62663	core	Probable manganese-dependent inorganic pyrophosphatase	Core	D	x	x	x	x
llmg_1062	LLNZ_05490	NCDO2118_RS07500	L63310	core	Deoxyribose-phosphate aldolase	Core	L	x	x	x	x
llmg_0634	LLNZ_03275	NCDO2118_RS03355	L63652	core	Pyruvate carboxylase	Core	F	x	x	x	x
llmg_0271	LLNZ_01420	NCDO2118_RS01345	L63684	core	Oxidoreductase	Core	C	x	x	x	x
llmg_2480	LLNZ_12815	NCDO2118_RS12020	L63900	core	Hypothetical protein	Core	C	x	x	x	x
llmg_1060	LLNZ_05480	NCDO2118_RS07510	L64175	core	Pyrimidine-nucleoside phosphorylase	Core	S	x	x	x	x
llmg_0075	LLNZ_00370	NCDO2118_RS00235	L64373	core	Lipoate-protein ligase A	Core	E	x	x	x	x
llmg_2482	LLNZ_12825	NCDO2118_RS12030	L64833	core	Hypothetical protein	Core	S	x	x	x	x
llmg_0273	LLNZ_01430	NCDO2118_RS01350	L65029	core	S-ribosylhomocysteine lyase	Core	S	x	x	x	x
llmg_2483	LLNZ_12830	NCDO2118_RS12165	L65440	core	Hypothetical protein	Core	T	x	x	x	x
llmg_1059	LLNZ_05475	NCDO2118_RS07515	L65498	core	16S rRNA methyltransferase	Core	S	x	x	x	x
llmg_1946	LLNZ_10020	NCDO2118_RS09695	L6563	core	ATP synthase subunit beta	Core	J	x	x	x	x
llmg_0871	LLNZ_04480	NCDO2118_RS08900	L66199	core	Aspartate-semialdehyde dehydrogenase	Core	J	x	x	x	x
llmg_0276	LLNZ_01445	NCDO2118_RS01360	L66233	core	Oxidoreductase	Core	R	x	x	x	x
llmg_0277	LLNZ_01450	NCDO2118_RS01365	L67226	core	Nicotinamidase	Core	C	x	x	x	x
llmg_2280	LLNZ_11790	NCDO2118_RS11020	L67463	core	Hypothetical protein	Core	G	x	x	x	x
llmg_2486	LLNZ_12845	NCDO2118_RS12180	L67624	core	Phosphatase	Core	S	x	x	x	x
llmg_2282	LLNZ_11800	NCDO2118_RS11030	L68401	core	S1 RNA-binding protein	Core	S	x	x	x	x
llmg_0280	LLNZ_01465	NCDO2118_RS01380	L68758	core	N-acetylmuramidase	Core	S	x	x	x	x
llmg_0080	LLNZ_00395	NCDO2118_RS00255	L68759	core	Peroxiredoxin	Core	M	x	x	x	x
llmg_0281	LLNZ_01470	NCDO2118_RS01385	L70400	core	ribonucleoside triphosphate reductase	Core	K	x	x	x	x
llmg_2285	LLNZ_11815	NCDO2118_RS11045	L70624	core	Uridylate kinase	Core	L	x	x	x	x
llmg_1514	LLNZ_07785	NCDO2118_RS05545	L71932	core	Transcriptional regulator	Core	S	x	x	x	x
llmg_0585	LLNZ_03000	NCDO2118_RS03130	L7226	core	Hypothetical protein	Core	S	x	x	x	x
llmg_0638	LLNZ_03295	NCDO2118_RS03375	L72391	core	ATP-dependent Clp protease proteolytic subunit	Core	S	x	x	x	x
llmg_1049	LLNZ_05425	NCDO2118_RS07560	L72477	core	ABC transporter glycine/betaine permease	Core	O	x	x	x	x
llmg_0085	LLNZ_00420	NCDO2118_RS00280	L73210	core	ABC transporter ATP-binding protein	Core	S	x	x	x	x
llmg_1512	LLNZ_07775	NCDO2118_RS05555	L73239	core	Multidrug ABC transporter ATP-binding protein	Core	U	x	x	x	x
llmg_2289	LLNZ_11830	NCDO2118_RS11060	L73818	core	Acetate kinase	Core	E	x	x	x	x
llmg_0640	LLNZ_03305	NCDO2118_RS03385	L73853	core	Arsenate reductase	Core	C	x	x	x	x
llmg_1048	LLNZ_05420	NCDO2118_RS07565	L74195	core	ABC transporter glycine/betaine, ATP-binding protein	Core	G	x	x	x	x
llmg_2290	LLNZ_11835	NCDO2118_RS11065	L74738	core	DNA methyltransferase	Core	S	x	x	x	x
llmg_0935	LLNZ_04810	NCDO2118_RS08200	L7563	core	Hypothetical protein	Core	S	x	x	x	x
llmg_1047	LLNZ_05415	NCDO2118_RS07570	L75633	core	GntR family transcriptional regulator	Core	I	x	x	x	x
llmg_1693	LLNZ_08735	NCDO2118_RS04465	L76582	core	bifunctional protein FolD	Core	H	x	x	x	x
llmg_2428	LLNZ_12550	NCDO2118_RS11770	L7722	core	septation ring formation regulator EzrA	Core	S	x	x	x	x
llmg_0290	LLNZ_01525	NCDO2118_RS01680	L78434	core	Thiol-disulfide isomerase	Core	S	x	x	x	x

llmg_0425	LLNZ_02215	NCDO2118_RS02315	L7866	core	Mevalonate kinase	Core	S	x	x	x	x
llmg_0291	LLNZ_01530	NCDO2118_RS01685	L79267	core	2,3,4,5-tetrahydropyridine-2,6-dicarboxylate N-acetyltransferase	Core	R	x	x	x	x
llmg_2018	LLNZ_10400	NCDO2118_RS10130	L79833	core	Hypothetical protein	Core	S	x	x	x	x
llmg_1509	LLNZ_07765	NCDO2118_RS05585	L80411	core	Orotate phosphoribosyltransferase	Core	E	x	x	x	x
llmg_1947	LLNZ_10025	NCDO2118_RS09700	L8105	core	ATP synthase subunit gamma	Core	V	x	x	x	x
llmg_1508	LLNZ_07760	NCDO2118_RS05590	L81189	core	Dihydroorotase	Core	C	x	x	x	x
llmg_2494	LLNZ_12885	NCDO2118_RS12235	L81616	core	Oxidoreductase	Core	G	x	x	x	x
llmg_1686	LLNZ_08695	NCDO2118_RS04495	L82250	core	Transcriptional regulator	Core	X	x	x	x	x
llmg_2495	LLNZ_12890	NCDO2118_RS12240	L82539	core	Hypothetical protein	Core	G	x	x	x	x
llmg_2298	LLNZ_11880	NCDO2118_RS11110	L83865	core	Hypothetical protein	Core	R	x	x	x	x
llmg_2299	LLNZ_11885	NCDO2118_RS11115	L84096	core	Glucokinase	Core	S	x	x	x	x
llmg_0787	LLNZ_04080	NCDO2118_RS08990	L84240	core	D-ribose transporter ATP-binding protein	Core	R	x	x	x	x
llmg_2023	LLNZ_10420	NCDO2118_RS10150	L84477	core	Universal stress protein UspA	Core	J	x	x	x	x
llmg_0297	LLNZ_01560	NCDO2118_RS01715	L84502	core	Hypothetical protein	Core	S	x	x	x	x
llmg_2300	LLNZ_11890	NCDO2118_RS11120	L85091	core	NADH dehydrogenase	Core	S	x	x	x	x
llmg_2302	LLNZ_11895	NCDO2118_RS11125	L85575	core	Ferritin	Core	P	x	x	x	x
llmg_1498	LLNZ_07710	NCDO2118_RS05615	L85764	core	Hypothetical protein	Core	G	x	x	x	x
llmg_1497	LLNZ_07705	NCDO2118_RS05620	L86251	core	Sugar phosphate phosphatase	Core	G	x	x	x	x
llmg_1885	LLNZ_09710	NCDO2118_RS03640	L87336	core	TatD family hydrolase	Core	Q	x	x	x	x
llmg_0299	LLNZ_01570	NCDO2118_RS01725	L87453	core	Adenosine deaminase	Core	L	x	x	x	x
llmg_1681	LLNZ_08665	NCDO2118_RS04525	L87561	core	16S rRNA methyltransferase	Core	F	x	x	x	x
llmg_0783	LLNZ_04060	NCDO2118_RS09010	L88187	core	Adenylosuccinate lyase	Core	J	x	x	x	x
llmg_0467	LLNZ_02415	NCDO2118_RS02515	L88252	core	CTP synthase	Core	F	x	x	x	x
llmg_0577	LLNZ_02965	NCDO2118_RS03085	L883	core	Methionine aminopeptidase	Core	F	x	x	x	x
llmg_2024	LLNZ_10425	NCDO2118_RS10170	L88446	core	Peptide-binding protein	Core	J	x	x	x	x
llmg_1679	LLNZ_08655	NCDO2118_RS04535	L89079	core	penicillin-binding protein 2X	Core	S	x	x	x	x
llmg_0302	LLNZ_01585	NCDO2118_RS01735	L89418	core	Ribonuclease J	Core	S	x	x	x	x
llmg_1948	LLNZ_10030	NCDO2118_RS09705	L8990	core	ATP synthase subunit alpha	Core	J	x	x	x	x
llmg_0781	LLNZ_04050	NCDO2118_RS09025	L90622	core	Hypothetical protein	Core	E	x	x	x	x
llmg_0476	LLNZ_02465	NCDO2118_RS02525	L90946	core	Hypothetical protein	Core	L	x	x	x	x
llmg_0304	LLNZ_01590	NCDO2118_RS01740	L91273	core	Hypothetical protein	Core	EP	x	x	x	x
llmg_2308	LLNZ_11920	NCDO2118_RS11150	L91456	core	Aminotransferase	Core	M	x	x	x	x
llmg_0306	LLNZ_01600	NCDO2118_RS01745	L91807	core	Peptidase M22	Core	P	x	x	x	x
llmg_2198	LLNZ_11345	NCDO2118_RS10705	L9255	core	Transcriptional regulator	Core	F	x	x	x	x
llmg_2504	LLNZ_12935	NCDO2118_RS12285	L92665	core	Aspartate racemase	Core	S	x	x	x	x
llmg_1878	LLNZ_09675	NCDO2118_RS03675	L92686	core	nitrogen utilization protein B	Core	M	x	x	x	x
llmg_2309	LLNZ_11925	NCDO2118_RS11155	L92850	core	Carbamate kinase	Core	K	x	x	x	x
llmg_1489	LLNZ_07665	NCDO2118_RS05660	L92886	core	phosphate starvation protein PhoH	Core	E	x	x	x	x
llmg_2505	LLNZ_12940	NCDO2118_RS12290	L93420	core	carboxylate--amine ligase	Core	E	x	x	x	x
llmg_2031	LLNZ_10470	NCDO2118_RS10205	L95240	core	Translation initiation factor IF-3	Core	S	x	x	x	x
llmg_0774	LLNZ_04020	NCDO2118_RS09055	L96847	core	Dipeptidase	Core	S	x	x	x	x
llmg_0933	LLNZ_04800	NCDO2118_RS08210	L9737	core	RNA-binding protein	Core	G	x	x	x	x
llmg_0772	LLNZ_04010	NCDO2118_RS09060	L98109	core	Hypothetical protein	Core	S	x	x	x	x
llmg_0492	LLNZ_02545	NCDO2118_RS02560	L98583	core	Fatty acid-binding protein	Core	M	x	x	x	x
llmg_2509	LLNZ_12960	NCDO2118_RS12310	L98749	core	rod shape-determining protein MreC	Core	S	x	x	x	x
llmg_2199	LLNZ_11350	NCDO2118_RS10710	L9964	core	Chitinase	Core	E	x	x	x	x
llmg_0204	LLNZ_01065	NCDO2118_RS00965	L0422	core	50S ribosomal protein L28	Core	R	x	x	x	x
llmg_1008	LLNZ_05195	NCDO2118_RS07765	L115968	core	GMP synthase	Core	E	x	x	x	x
llmg_0003	LLNZ_00015	NCDO2118_RS00015	L0252	core	ATP-dependent helicase/deoxyribonuclease subunit B	Shared	L	x	x		
llmg_0017	LLNZ_00080	NCDO2118_RS00090	L22059	core	Hypothetical protein	Shared	H	x	x		
llmg_0018	LLNZ_00085	NCDO2118_RS00095	L22496	core	beta-lactamase	Shared	G	x	x		
llmg_0092	LLNZ_00460	NCDO2118_RS00305	L77482	core	putative short-chain type dehydrogenase	Shared	S	x	x		
llmg_0099	LLNZ_00495	NCDO2118_RS00340	L00096	core	50S ribosomal protein L32	Shared	J	x	x		x
llmg_0111	LLNZ_00555	NCDO2118_RS00390	L105256	core	16S ribosomal RNA methyltransferase RsmE	Shared	P	x	x		
llmg_0139	LLNZ_00735	NCDO2118_RS00580	L0114	core	argininosuccinate lyase	Shared	E	x	x		
llmg_0141	LLNZ_00745	NCDO2118_RS00590	L130713	core	putative transcriptional regulator	Shared	E	x	x		
llmg_0142	LLNZ_00750	NCDO2118_RS00595	L131443	core	ribonuclease P	Shared	G	x	x		
llmg_0183	LLNZ_00955	NCDO2118_RS00850	L174251	core	Hypothetical protein	Shared	S	x	x		
llmg_0212	LLNZ_01105	NCDO2118_RS01010	L2599	core	rhamnosyltransferase RgpB	Shared	K	x	x		
llmg_0216	LLNZ_01125	NCDO2118_RS01030	L6907	core	glycosyltransferase RgpE	Shared	G	x	x		
llmg_0217	LLNZ_01130	NCDO2118_RS01035	L7894	core	alpha-L-Rha alpha-1,2-L-rhamnosyltransferase RgpF	Shared	I	x	x		
llmg_0219	LLNZ_01140	NCDO2118_RS01050	L11986	core	Hypothetical protein	Shared	E	x	x		
llmg_0263	LLNZ_01380	NCDO2118_RS01290	L52686	core	SAM-dependent methyltransferase	Shared	J	x	x	x	
llmg_0265	LLNZ_01390	NCDO2118_RS01305	L56208	core	putative flavoprotein	Shared	X	x	x		
llmg_0266	LLNZ_01395	NCDO2118_RS01310	L57608	core	putative sugar kinase	Shared	C	x	x		
llmg_0287	LLNZ_01510	NCDO2118_RS01665	L75718	core	Cobalt ABC transporter ATP-binding protein	Shared	K	x	x	x	
llmg_0311	LLNZ_01625	NCDO2118_RS01775	L95210	core	Hypothetical protein	Shared	M	x	x		
llmg_0336	LLNZ_01765	NCDO2118_RS01905	L118475	core	D-methionine-binding lipoprotein plpB precursor]	Shared	S	x	x		
llmg_0344	LLNZ_01800	NCDO2118_RS01935	L123777	core	putative cobalt ABC transporter ATP-binding protein	Shared	E	x	x		
llmg_0345	LLNZ_01805	NCDO2118_RS01940	L125503	core	putative cobalt ABC transporter permease protein	Shared	R	x	x		
llmg_0357	LLNZ_01870	NCDO2118_RS02005	L135954	core	alkyl hydroperoxide reductase subunit F	Shared	G	x	x		
llmg_0372	LLNZ_01945	NCDO2118_RS02075	L00396	core	asparagine synthetase B	Shared	E	x	x		
llmg_0373	LLNZ_01950	NCDO2118_RS02080	L0271	core	formamidopyrimidine-DNA glycosylase	Shared	L	x	x		
llmg_0377	LLNZ_01970	NCDO2118_RS02100	L162952	core	Hypothetical protein	Shared	H	x	x		
llmg_0383	LLNZ_02000	NCDO2118_RS02130	L166614	core	inorganic polyphosphate/ATP-NAD kinase	Shared	P	x	x		
llmg_0385	LLNZ_02010	NCDO2118_RS02140	L168484	core	peptidyl-prolyl cis-trans isomerase, cyclophilin-type	Shared	U	x	x		
llmg_0412	LLNZ_02145	NCDO2118_RS02270	L582	core	Ribonuclease Z	Shared	U	x	x		
llmg_0413	LLNZ_02155	NCDO2118_RS02275	L0124	core	sensor histidine kinase	Shared	T	x	x		
llmg_0416	LLNZ_02170	NCDO2118_RS02290	L0280	core	DNA polymerase III subunit delta'	Shared	L	x	x		
llmg_0417	LLNZ_02175	NCDO2118_RS02295	L5532	core	Hypothetical protein	Shared	S	x	x		
llmg_0418	LLNZ_02180	NCDO2118_RS02300	L6328	core	DNA replication initiation control protein YabA	Shared	S	x	x		
llmg_0419	LLNZ_02185	NCDO2118_RS02305	L6615	core	16S rRNA methyltransferase	Shared	L	x	x	x	
llmg_0426	LLNZ_02220	NCDO2118_RS02320	L9089	core	Diphosphomevalonate decarboxylase	Shared	J	x	x		
llmg_0428	LLNZ_02230	NCDO2118_RS02330	L11083	core	isopentenyl pyrophosphate isomerase	Shared	S	x	x		
llmg_0440	LLNZ_02285	NCDO2118_RS02375	L20847	core	cellobiose-specific PTS system IIC component	Shared	S	x	x		
llmg_0445	LLNZ_02305	NCDO2118_RS02400	L26628	core	putative lipid kinase	Shared	S	x	x		
llmg_0447	LLNZ_02315	NCDO2118_RS02410	L29321	core	NifJ protein	Shared	S	x	x		
llmg_0448	LLNZ_02320	NCDO2118_RS02415	L33187	core	Hypothetical protein	Shared	G	x	x		
llmg_0452	LLNZ_02340	NCDO2118_RS02430	L0150	core	trehalose operon transcriptional repressor	Shared	K	x	x		
llmg_0453	LLNZ_02345	NCDO2118_RS02435	L37338	core	sucrose-specific PTS enzyme IIABC	Shared	R	x	x		
llmg_0461	LLNZ_02385	NCDO2118_RS02480	L45616	core	transcription regulator	Shared	E	x	x		
llmg_0462	LLNZ_02390	NCDO2118_RS02490	L0331	core	tRNA pseudouridine synthase A	Shared	J	x	x		
llmg_0463	LLNZ_02395	NCDO2118_RS02495	L0202	core	phosphomethylpyrimidine kinase	Shared	F	x	x		
llmg_0465	LLNZ_02405	NCDO2118_RS02505	L86338	core	Hypothetical protein	Shared	P	x	x	x	
llmg_0466	LLNZ_02410	NCDO2118_RS02510	L86881	core	putative aminotransferase	Shared	S	x	x		
llmg_0478	LLNZ_02475	NCDO2118_RS02535	L92464	core	Nucleoside deoxyribosyltransferase	Shared	R	x	x	x	
llmg_0507	LLNZ_02620	NCDO2118_RS02630	L124415	core	Peptide ABC transporter substrate-binding protein	Shared	S	x	x		x
llmg_0520	LLNZ_02690	NCDO2118_RS02785	L0269	core	DNA primase	Shared	L	x	x		
llmg_0535	LLNZ_02760	NCDO2118_RS02840	L158343	core	arginine-binding periplasmic protein 1 precursor	Shared	P	x	x		
llmg_0541	LLNZ_02785	NCDO2118_RS02880	L165449	core	Hydrolase	Shared	F	x	x	x	
llmg_0556	LLNZ_02855	NCDO2118_RS02995	L185472	core	Hypothetical protein	Shared	S	x	x		x
llmg_0570	LLNZ_02920	NCDO2118_RS03060	L196779	core	rRNA methyltransferase	Shared	C	x	x		
llmg_0606	LLNZ_03110	NCDO2118_RS03260	L0259	core	single strand DNA-specific exonuclease	Shared	L	x	x		
llmg_0614	LLNZ_03160	NCDO2118_RS03285	L0223	core	transcriptional regulator CtsR	Shared	K	x	x		
llmg_0616	LLNZ_03170	NCDO2118_RS03295	L32389	core	putative sigma 54 modulation protein	Shared	E	x	x		
llmg_0630	LLNZ_03255	NCDO2118_RS03335	L59930	core	dephospho-CoA kinase	Shared	E	x	x		
llmg_0639	LLNZ_03300	NCDO2118_RS03380	L73160	core	Hypothetical protein	Shared	P	x	x		
llmg_0698	LLNZ_03630	NCDO2118_RS10185	L92192	core	oligopeptide transport ATP-binding protein oppF	Shared	J	x	x		

llmg_0729	LLNZ_03795	NCDO2118_RS09370	L1762179	core	PTS system, mannose-specific IIAB components	Shared	C	x	x		
llmg_0739	LLNZ_03850	NCDO2118_RS09235	L128695	core	Maltose ABC transporter substrate-binding protein	Shared	G	x	x	x	
llmg_0746	LLNZ_03885	NCDO2118_RS09200	L0144	core	LacI family transcription regulator	Shared	K	x	x		
llmg_0752	LLNZ_03915	NCDO2118_RS09170	L118462	core	phage infection protein	Shared	R	x	x		
llmg_0768	LLNZ_03995	NCDO2118_RS09080	L102491	core	DNA polymerase III subunit delta	Shared	G	x	x		
llmg_0782	LLNZ_04055	NCDO2118_RS09015	L0062	core	3-dehydroquinate dehydratase	Shared	E	x	x		
llmg_0857	LLNZ_04405	NCDO2118_RS08970	L0240	core	GntR family transcription regulator	Shared	K	x	x		
llmg_0885	LLNZ_04555	NCDO2118_RS08835	L0117	core	gamma-glutamyl kinase	Shared	E	x	x		
llmg_0888	LLNZ_04570	NCDO2118_RS08820	L0249	core	ArsR family transcriptional regulator	Shared	K	x	x		
llmg_0904	LLNZ_04645	NCDO2118_RS03535	L41820	core	immunogenic secreted protein precursor-like protein	Shared	S	x	x		
llmg_0931	LLNZ_04790	NCDO2118_RS08220	L10433	core	MvaA protein	Shared	S	x	x		
llmg_0936	LLNZ_04815	NCDO2118_RS08195	L6876	core	16S rRNA-processing protein RimM	Shared	S	x	x		
llmg_0939	LLNZ_04830	NCDO2118_RS08180	L4747	core	DegV family protein	Shared	L	x	x		
llmg_0942	LLNZ_04845	NCDO2118_RS08165	L0324	core	tRNA CCA-pyrophosphorylase	Shared	J	x	x		
llmg_0951	LLNZ_04885	NCDO2118_RS08125	L193644	core	MsrA protein	Shared	I	x	x		
llmg_0995	LLNZ_05125	NCDO2118_RS07945	L157841	core	hydrolase, haloacid dehalogenase-like family protein	Shared	S	x	x		
llmg_0996	LLNZ_05140	NCDO2118_RS07935	L154512	core	TetR family transcriptional regulator	Shared	P	x	x		
llmg_1017	LLNZ_05240	NCDO2118_RS07720	L110960	core	MarR family transcriptional regulator	Shared	R	x	x		
llmg_1024	LLNZ_05285	NCDO2118_RS07665	L104745	core	Hypothetical protein	Shared	S	x	x		
llmg_1028	LLNZ_05305	NCDO2118_RS07645	L102062	core	NAD(P)H nitroreductase	Shared	D	x	x	x	
llmg_1038	LLNZ_05355	NCDO2118_RS07620	L0051	core	TrpC protein	Shared	E	x	x		
llmg_1046	LLNZ_05410	NCDO2118_RS07585	L89194	core	beta-glucosidase	Shared	R	x	x		
llmg_1063	LLNZ_05495	NCDO2118_RS07495	L62931	core	cytidine deaminase	Shared	X	x	x		
llmg_1067	LLNZ_05515	NCDO2118_RS07475	L0122	core	sensor protein kinase kinB	Shared	T	x	x		
llmg_1100	LLNZ_05690	NCDO2118_RS07325	L189881	core	ribosomal large subunit pseudouridine synthase	Shared	S	x	x		
llmg_1105	LLNZ_05715	NCDO2118_RS07260	L183563	core	dihydroorotate dehydrogenase electron transfer subunit	Shared	S	x	x		
llmg_1107	LLNZ_05725	NCDO2118_RS07250	L181692	core	orotidine 5'-phosphate decarboxylase	Shared	R	x	x		
llmg_1133	LLNZ_05850	NCDO2118_RS07125	L152588	core	nuclease sbcCD subunit C	Shared	E	x	x		
llmg_1134	LLNZ_05855	NCDO2118_RS07120	L152227	core	Hypothetical protein	Shared	S	x	x		
llmg_1138	LLNZ_05875	NCDO2118_RS07100	L148957	core	Manganese ABC transporter substrate-binding protein	Shared	S	x	x	x	
llmg_1171	LLNZ_06030	NCDO2118_RS06895	L132615	core	Hypothetical protein	Shared	K	x	x		
llmg_1180	LLNZ_06075	NCDO2118_RS06850	L122632	core	small conductance mechanosensitive ion channel, MscS family	Shared	P	x	x		
llmg_1185	LLNZ_06100	NCDO2118_RS06825	L0121	core	diaminopimelate decarboxylase	Shared	E	x	x		
llmg_1191	LLNZ_06130	NCDO2118_RS06795	L109747	core	site-specific tyrosine recombinase XerD-like protein	Shared	J	x	x		
llmg_1203	LLNZ_06190	NCDO2118_RS06765	L102979	core	ABC transporter ABC binding and permease protein	Shared	S	x	x		
llmg_1219	LLNZ_06280	NCDO2118_RS06705	L91510	core	D-alanine--polyphosphoribitol)	Shared	R	x	x		
llmg_1223	LLNZ_06300	NCDO2118_RS06685	L85514	core	Magnesium-transporting ATPase	Shared	S	x	x	x	
llmg_1224	LLNZ_06305	NCDO2118_RS06680	L84767	core	transcriptional regulator	Shared	K	x	x		
llmg_1258	LLNZ_06480	NCDO2118_RS01430	L66407	core	Hypothetical protein	Shared	R	x	x		
llmg_1287	LLNZ_06650	NCDO2118_RS06385	L38177	core	Hypothetical protein	Shared	E	x	x		
llmg_1304	LLNZ_06745	NCDO2118_RS06320	L25961	core	putative exoribonuclease R	Shared	S	x	x		
llmg_1310	LLNZ_06775	NCDO2118_RS06145	L198787	core	Hypothetical protein	Shared	O	x	x		
llmg_1313	LLNZ_06785	NCDO2118_RS06135	L196904	core	Hypothetical protein	Shared	R	x	x		
llmg_1314	LLNZ_06790	NCDO2118_RS06130	L195271	core	Hypothetical protein	Shared	S	x	x		
llmg_1329	LLNZ_06875	NCDO2118_RS06045	L173881	core	UDP-N-acetylenolpyruvoylglucosamine reductase	Shared	S	x	x	x	
llmg_1331	LLNZ_06885	NCDO2118_RS06035	L0091	core	homoserine kinase	Shared	E	x	x		
llmg_1332	LLNZ_06890	NCDO2118_RS06030	L0090	core	homoserine dehydrogenase	Shared	E	x	x		
llmg_1336	LLNZ_06910	NCDO2118_RS06010	L0176	core	dihydropteroate synthase	Shared	F	x	x		
llmg_1428	LLNZ_07360	NCDO2118_RS05880	L143292	core	Hypothetical protein	Shared	K	x	x		
llmg_1429	LLNZ_07365	NCDO2118_RS05875	L142159	core	L-lactate dehydrogenase	Shared	S	x	x		
llmg_1450	LLNZ_07475	NCDO2118_RS05805	L124107	core	thiamine biosynthesis lipoprotein apbE precursor	Shared	S	x	x		
llmg_1452	LLNZ_07485	NCDO2118_RS05795	L119780	core	putative amino-acid permease	Shared	S	x	x		
llmg_1453	LLNZ_07490	NCDO2118_RS05790	L119013	core	oxidoreductase	Shared	KG	x	x		
llmg_1464	LLNZ_07545	NCDO2118_RS05780	L0322	core	AldC protein	Shared	C	x	x		
llmg_1484	LLNZ_07640	NCDO2118_RS05685	L0319	core	putative late competence protein	Shared	R	x	x		
llmg_1499	LLNZ_07715	NCDO2118_RS05610	L84937	core	Hypothetical protein	Shared	K	x	x		
llmg_1500	LLNZ_07720	NCDO2118_RS05605	L84257	core	putative methyltransferase	Shared	J	x	x		
llmg_1536	LLNZ_07895	NCDO2118_RS05455	L9876	core	ElaA protein	Shared	D	x	x		
llmg_1539	LLNZ_07910	NCDO2118_RS05440	L0290	core	DNA topoisomerase IV subunit B	Shared	L	x	x		
llmg_1542	LLNZ_07925	NCDO2118_RS05425	L4702	core	ribonucleotide reductase stimulatory protein	Shared	G	x	x		
llmg_1545	LLNZ_07940	NCDO2118_RS05410	L2	core	Cell division protein FtsX	Shared	S	x	x	x	
llmg_1572	LLNZ_08095	NCDO2118_RS05320	L180241	core	myosin-cross-reactive antigen	Shared	R	x	x		
llmg_1576	LLNZ_08115	NCDO2118_RS05305	L0212	core	HrcA family transcriptional regulator	Shared	K	x	x		x
llmg_1585	LLNZ_08160	NCDO2118_RS05260	L169230	core	Putative amidase	Shared	R	x	x		
llmg_1586	LLNZ_08165	NCDO2118_RS05255	L0323	core	putative exoribonuclease R	Shared	K	x	x		
llmg_1643	LLNZ_08455	NCDO2118_RS04710	L115706	core	rod shape-determining protein RodA	Shared	S	x	x		
llmg_1678	LLNZ_08645	NCDO2118_RS04540	L91444	core	phospho-N-acetylmuramoyl-pentapeptide-transferase	Shared	S	x	x		
llmg_1680	LLNZ_08660	NCDO2118_RS04530	L88699	core	Cell division protein FtsL	Shared	S	x	x		x
llmg_1685	LLNZ_08690	NCDO2118_RS04500	L0264	core	DNA repair protein recN	Shared	L	x	x		
llmg_1689	LLNZ_08710	NCDO2118_RS04485	L80459	core	farnesyl-diphosphate synthase	Shared	F	x	x		
llmg_1692	LLNZ_08730	NCDO2118_RS04470	L0254	core	exodeoxyribonuclease VII large subunit	Shared	L	x	x		
llmg_1702	LLNZ_08775	NCDO2118_RS04400	L0197	core	Glutathione reductase	Shared	V	x	x		x
llmg_1726	LLNZ_08915	NCDO2118_RS04345	L51063	core	galactose-1-phosphate uridylyltransferase	Shared	HE	x	x		
llmg_1729	LLNZ_08930	NCDO2118_RS04335	L45966	core	copper/potassium-transporting ATPase	Shared	K	x	x		
llmg_1756	LLNZ_09070	NCDO2118_RS04200	L0106	core	Acetylornithine aminotransferase	Shared	E	x	x	x	
llmg_1787	LLNZ_09215	NCDO2118_RS04060	L0182	core	3-oxoacyl-(acyl carrier protein) synthase III	Shared	I	x	x		
llmg_1788	LLNZ_09220	NCDO2118_RS04055	L180805	core	MarR family transcriptional regulator	Shared	R	x	x		x
llmg_1817	LLNZ_09355	NCDO2118_RS03915	L150333	core	putative beta-phosphoglucomutase	Shared	J	x	x		
llmg_1856	LLNZ_09555	NCDO2118_RS03750	L116532	core	multidrug resistance ABC transporter ATP-binding and permease protei	Shared	S	x	x		
llmg_1899	LLNZ_09780	NCDO2118_RS09470	L157144	core	phosphate ABC transporter, permease protein PstC	Shared	H	x	x		
llmg_1902	LLNZ_09795	NCDO2118_RS09485	L160560	core	SsrA-binding protein	Shared	R	x	x		
llmg_1909	LLNZ_09830	NCDO2118_RS09510	L166370	core	oligoendopeptidase F	Shared	S	x	x		
llmg_1913	LLNZ_09850	NCDO2118_RS09530	L171588	core	Guanine permease	Shared	S	x	x	x	
llmg_1927	LLNZ_09920	NCDO2118_RS09600	L0058	core	prephenate dehydrogenase	Shared	E	x	x		
llmg_1936	LLNZ_09975	NCDO2118_RS09645	L195366	core	Hypothetical protein	Shared	O	x	x		
llmg_1944	LLNZ_10010	NCDO2118_RS09685	L5196	core	Hypothetical protein	Shared	S	x	x		
llmg_1953	LLNZ_10055	NCDO2118_RS09730	L12887	core	Esterase	Shared	S	x	x	x	
llmg_1961	LLNZ_10110	NCDO2118_RS09770	L0228	core	transcriptional activator TenA	Shared	K	x	x		
llmg_1977	LLNZ_10210	NCDO2118_RS09855	L37085	core	Adapter protein MecA	Shared	S	x	x	x	
llmg_1985	LLNZ_10250	NCDO2118_RS09895	L49741	core	neutral endopeptidase O2	Shared	G	x	x		
llmg_1986	LLNZ_10255	NCDO2118_RS09900	L51690	core	Methylated-DNA--protein-cysteine methyltransferase	Shared	G	x	x	x	
llmg_1988	LLNZ_10260	NCDO2118_RS09905	L52064	core	Hypothetical protein	Shared	O	x	x		
llmg_1999	LLNZ_10310	NCDO2118_RS09955	L64811	core	Hypothetical protein	Shared	E	x	x		
llmg_2004	LLNZ_10330	NCDO2118_RS10070	L67527	core	Hypothetical protein	Shared	S	x	x		
llmg_2012	LLNZ_10370	NCDO2118_RS10105	L75267	core	Oxidoreductase	Shared	J	x	x	x	
llmg_2035	LLNZ_10490	NCDO2118_RS10220	L97777	core	tRNA uridine 5-carboxymethylaminomethyl modification enzyme GidA	Shared	P	x	x		
llmg_2036	LLNZ_10495	NCDO2118_RS10225	L100027	core	Hypothetical protein	Shared	J	x	x		
llmg_2042	LLNZ_10520	NCDO2118_RS10250	L0087	core	serine acetyltransferase	Shared	E	x	x		
llmg_2043	LLNZ_10525	NCDO2118_RS10255	L104285	core	Hypothetical protein	Shared	J	x	x		
llmg_2056	LLNZ_10585	NCDO2118_RS10310	L118668	core	cell division protein	Shared	S	x	x		
llmg_2058	LLNZ_10595	NCDO2118_RS10320	L119731	core	Cell division protein SepF	Shared	U	x	x	x	
llmg_2079	LLNZ_10695	NCDO2118_RS10415	L138452	core	serine/threonine protein kinase	Shared	M	x	x		
llmg_2086	LLNZ_10730	NCDO2118_RS08525	L77514	core	serine/threonine-rich protein precursor	Shared	R	x	x		
llmg_2089	LLNZ_10745	NCDO2118_RS05150	L25762	core	phage tail component	Shared	F	x	x		
llmg_2096	LLNZ_10780	NCDO2118_RS08570	L33661	core	putative major head protein precursor	Shared	C	x	x		
llmg_2157	LLNZ_11125	NCDO2118_RS10490	L152419	core	6-phosphogluconolactonase	Shared	S	x	x	x	
llmg_2204	LLNZ_11375	NCDO2118_RS10760	L17654	core	Heat-shock protein Hsp33	Shared	G	x	x	x	
llmg_2209	LLNZ_11400	NCDO2118_RS10770	L19272	core	tRNA-dihydrouridine synthase B	Shared	F	x	x		

llmg_2227	LLNZ_11480	NCDO2118_RS10850	L32666	core	Hypothetical protein	Shared	F	x	x		
llmg_2229	LLNZ_11490	NCDO2118_RS10860	L0261	core	Recombination protein F	Shared	L	x	x		
llmg_2230	LLNZ_11495	NCDO2118_RS10865	L35675	core	gamma-carboxymuconolactone decarboxylase	Shared	X	x	x		
llmg_2272	LLNZ_11750	NCDO2118_RS10980	L58914	core	Hypothetical protein	Shared	E	x	x		
llmg_2273	LLNZ_11755	NCDO2118_RS10985	L60959	core	Histidine triad protein	Shared	S	x	x		x
llmg_2306	LLNZ_11910	NCDO2118_RS11140	L89201	core	Hypothetical protein	Shared	G	x	x		
llmg_2320	LLNZ_11975	NCDO2118_RS11210	L107379	core	Hypothetical protein	Shared	S	x	x		
llmg_2327	LLNZ_12020	NCDO2118_RS11240	L117074	core	putative glycerol uptake facilitator protein	Shared	K	x	x		
llmg_2329	LLNZ_12030	NCDO2118_RS11250	L120396	core	Glutamine amidotransferase	Shared	S	x	x	x	
llmg_2332	LLNZ_12045	NCDO2118_RS11265	L0349	core	Glutamate--tRNA ligase	Shared	J	x		x	x
llmg_2341	LLNZ_12100	NCDO2118_RS11305	L129431	core	Hypothetical protein	Shared	P	x	x		
llmg_2396	LLNZ_12375	NCDO2118_RS11580	L164789	core	ribosomal large subunit pseudouridine synthase	Shared	F	x	x		
llmg_2409	LLNZ_12450	NCDO2118_RS11650	L0278	core	DNA polymerase III PolC	Shared	L	x	x		
llmg_2449	LLNZ_12665	NCDO2118_RS11870	L29314	core	Hypothetical protein	Shared	M	x	x		
llmg_2463	LLNZ_12730	NCDO2118_RS11915	L36841	core	Hypothetical protein	Shared	K	x	x		
llmg_2468	LLNZ_12755	NCDO2118_RS11960	L52019	core	gluconate kinase	Shared	S	x	x		
llmg_2469	LLNZ_12760	NCDO2118_RS11965	L53699	core	6-phosphogluconate dehydrogenase	Shared	X	x	x		x
llmg_2474	LLNZ_12785	NCDO2118_RS11990	L0302	core	Single-strand binding protein	Shared	L	x	x		
llmg_2479	LLNZ_12810	NCDO2118_RS12015	L63227	core	Hypothetical protein	Shared	S	x	x		
llmg_2518	LLNZ_13005	NCDO2118_RS12355	L109527	core	ribosomal small subunit pseudouridine synthase A	Shared	M	x	x		
llmg_2543	LLNZ_13135	NCDO2118_RS12405	L121175	core	Hypothetical protein	Shared	R	x	x		
llmg_1099	LLNZ_05685	NCDO2118_RS06630	L0014	core	Glycerol kinase	Shared	C			x	x
llmg_0859	LLNZ_04415	NCDO2118_RS08960	L0020	core	Mannonate dehydratase	Shared	G			x	x
llmg_2234	LLNZ_11515	NCDO2118_RS10895	L0027	core	Peptidase S24	Shared	E			x	x
llmg_2236	LLNZ_11525	NCDO2118_RS10905	L0029	core	Aldose 1-epimerase	Shared	G			x	x
llmg_2190	LLNZ_11305	NCDO2118_RS10665	L0047	core	ribulose-phosphate 3-epimerase	Shared	G		x	x	x
llmg_1291	LLNZ_06670	NCDO2118_RS06365	L0070	core	1-(5-phosphoribosyl)-5-[(5- phosphoribosylamino)methylideneamino]	Shared	E			x	x
llmg_pseudo	LLNZ_05385	NCDO2118_RS07595	L0154	core	Transcription antiterminator BglG	Shared	K		x	x	x
llmg_1829	LLNZ_09410	NCDO2118_RS03855	L0169	core	2-succinyl-5-enolpyruvyl-6-hydroxy-3- cyclohexene-1-carboxylate synth	Shared	H			x	x
llmg_2321	LLNZ_11985	NCDO2118_RS11220	L0199	core	Pyruvate oxidase	Shared	C			x	x
llmg_0350	LLNZ_01835	NCDO2118_RS01970	L0219	core	LysR family transcriptional regulator	Shared	K			x	x
llmg_0911	LLNZ_04680	NCDO2118_RS08430	L0235	core	Nitrogen regulatory protein P-II	Shared	E			x	x
llmg_1808	LLNZ_09310	NCDO2118_RS03965	L0281	core	primosomal protein DnaI	Shared	L			x	x
llmg_1880	LLNZ_09685	NCDO2118_RS03665	L0377	core	Elongation factor P	Shared	J		x	x	x
llmg_0494	LLNZ_02555	NCDO2118_RS02570	L100350	core	Beta-N-acetylhexosaminidase	Shared	L			x	x
llmg_0065	LLNZ_00325	NCDO2118_RS00195	L1005	core	Acetyltransferase	Shared	P			x	x
llmg_0076	LLNZ_00375	NCDO2118_RS00240	L1007	core	glycerophosphodiester phosphodiesterase	Shared	L		x	x	
llmg_2037	LLNZ_10500	NCDO2118_RS10230	L100735	core	Hypothetical protein	Shared	I			x	x
llmg_1752	LLNZ_09050	NCDO2118_RS04220	L10155	core	Chromosome segregation protein SMC	Shared	J		x	x	
llmg_2514	LLNZ_12985	NCDO2118_RS12335	L103195	core	Universal stress protein UspA	Shared	K			x	x
llmg_2515	LLNZ_12990	NCDO2118_RS12340	L103652	core	Hypothetical protein	Shared	S			x	x
llmg_2516	LLNZ_12995	NCDO2118_RS12345	L104115	core	2-hydroxyglutaryl-CoA dehydratase	Shared	S		x	x	x
llmg_0112	LLNZ_00560	NCDO2118_RS00395	L106031	core	polysaccharide deacetylase	Shared	G			x	x
llmg_1189	LLNZ_06120	NCDO2118_RS06805	L110933	core	Metallophosphoesterase	Shared	V			x	x
llmg_1646	LLNZ_08470	NCDO2118_RS04695	L113864	core	peptidyl-prolyl cis-trans isomerase	Shared	P			x	x
llmg_2051	LLNZ_10565	NCDO2118_RS10295	L114099	core	Hypothetical protein	Shared	S			x	x
llmg_1860	LLNZ_09575	NCDO2118_RS03735	L114370	core	ArsR family transcriptional regulator	Shared	R			x	x
llmg_2524	LLNZ_13030	NCDO2118_RS12380	L115500	core	alkylphosphonate transporter	Shared	CH			x	x
llmg_0123	LLNZ_00620	NCDO2118_RS00440	L115551	core	FMN-dependent NADH-azoreductase 1	Shared	R			x	x
llmg_1857	LLNZ_09560	NCDO2118_RS03745	L115789	core	Lysophospholipase	Shared	D			x	x
llmg_1641	LLNZ_08445	NCDO2118_RS04720	L118271	core	Acetoin reductase	Shared	R			x	x
llmg_1179	LLNZ_06070	NCDO2118_RS06855	L123581	core	Glutamate decarboxylase	Shared	S			x	x
llmg_1849	LLNZ_09515	NCDO2118_RS03785	L124252	core	Hypothetical protein	Shared	H			x	x
llmg_1843	LLNZ_09485	NCDO2118_RS03800	L127182	core	Deaminase	Shared	Q			x	x
llmg_0511	LLNZ_02640	NCDO2118_RS02650	L129183	core	penicillin-binding protein 1A 23	Shared	J		x	x	x
llmg_0430	LLNZ_02240	NCDO2118_RS02340	L13150	core	carbon starvation protein CstA	Shared	C			x	x
llmg_0515	LLNZ_02660	NCDO2118_RS02670	L133111	core	Hypothetical protein	Shared	L			x	x
llmg_0353	LLNZ_01850	NCDO2118_RS01985	L133547	core	MarR family transcriptional regulator	Shared	S			x	x
llmg_1442	LLNZ_07430	NCDO2118_RS05850	L136968	core	Hypothetical protein	Shared	E			x	x
llmg_1823	LLNZ_09385	NCDO2118_RS03880	L142722	core	Asparaginase	Shared	S		x	x	x
llmg_2143	LLNZ_11055	NCDO2118_RS10430	L142733	core	Transcriptional regulator	Shared	E			x	x
llmg_0928	LLNZ_04775	NCDO2118_RS08235	L14408	core	Glucosamine-6-phosphate deaminase	Shared	S		x	x	x
llmg_1426	LLNZ_07350	NCDO2118_RS05890	L145238	core	Sucrose-specific PTS system IIBC component	Shared	S			x	x
llmg_0157	LLNZ_00825	NCDO2118_RS00655	L145301	core	TetR family transcriptional regulator	Shared	G			x	x
llmg_2154	LLNZ_11110	NCDO2118_RS10475	L149295	core	DNA-directed RNA polymerase subunit omega	Shared	M			x	x
llmg_0999	LLNZ_05155	NCDO2118_RS07925	L152487	core	Phosphoribosyl carboxyaminoimidazole mutase	Shared	G			x	x
llmg_0997	LLNZ_05145	NCDO2118_RS07930	L153005	core	Phosphoribosylamine--glycine ligase	Shared	I			x	x
llmg_1416	LLNZ_07300	NCDO2118_RS05935	L155044	core	dCMP deaminase	Shared	S			x	x
llmg_1812	LLNZ_09330	NCDO2118_RS03940	L155396	core	Phosphotransferase	Shared	F			x	x
llmg_2165	LLNZ_11170	NCDO2118_RS10525	L159324	core	N-acetylmuramoyl-L-alanine amidase	Shared	S			x	x
llmg_0592	LLNZ_03040	NCDO2118_RS03225	L15964	core	Hypothetical protein	Shared	Q			x	x
llmg_0993	LLNZ_05115	NCDO2118_RS07955	L160442	core	Hypoxanthine phosphoribosyltransferase	Shared	I			x	x
llmg_1593	LLNZ_08195	NCDO2118_RS05225	L162009	core	ABC-type amino acid transport system, substrate-binding protein	Shared	J			x	x
llmg_0989	LLNZ_05090	NCDO2118_RS07970	L162870	core	ABC transporter ATP-binding protein	Shared	G			x	x
llmg_2393	LLNZ_12360	NCDO2118_RS11565	L163025	core	Membrane protein	Shared	S			x	x
llmg_1338	LLNZ_06920	NCDO2118_RS06000	L165490	core	Dihydroneopterin aldolase	Shared	G			x	x
llmg_0382	LLNZ_01995	NCDO2118_RS02125	L166082	core	GTP pyrophosphokinase	Shared	S			x	x
llmg_0544	LLNZ_02800	NCDO2118_RS02895	L167675	core	Phosphopantothenate--cysteine ligase	Shared	J			x	x
llmg_0546	LLNZ_02805	NCDO2118_RS02900	L168282	core	Tautomerase	Shared	K			x	x
llmg_1915	LLNZ_09860	NCDO2118_RS09540	L174321	core	Fe-S oxidoreductase	Shared	S			x	x
llmg_0552	LLNZ_02835	NCDO2118_RS02950	L177700	core	Glyoxalase	Shared	S			x	x
llmg_0972	LLNZ_04995	NCDO2118_RS08030	L178204	core	Hypothetical protein	Shared	IR			x	x
llmg_1109	LLNZ_05735	NCDO2118_RS07240	L180469	core	Ribosomal RNA small subunit methyltransferase G	Shared	L			x	x
llmg_1923	LLNZ_09900	NCDO2118_RS09580	L182916	core	Phosphoglycerate mutase	Shared	I			x	x
llmg_2416	LLNZ_12490	NCDO2118_RS11685	L184708	core	Preprotein translocase subunit YajC	Shared	J		x		x
llmg_1196	LLNZ_06155	NCDO2118_RS01085	L18600	core	Glycerophosphoryl diester phosphodiesterase	Shared	J			x	x
llmg_0954	LLNZ_04900	NCDO2118_RS08110	L191436	core	Hypothetical protein	Shared	M			x	x
llmg_2423	LLNZ_12525	NCDO2118_RS11720	L192043	core	Hypothetical protein	Shared	S		x	x	x
llmg_1932	LLNZ_09950	NCDO2118_RS09625	L192122	core	ABC transporter ATP-binding protein	Shared	S			x	x
llmg_1772	LLNZ_09150	NCDO2118_RS04125	L193291	core	Hypothetical protein	Shared	S			x	x
llmg_0568	LLNZ_02915	NCDO2118_RS03055	L196178	core	Acylphosphatase	Shared	S			x	x
llmg_0016	LLNZ_00075	NCDO2118_RS00085	L21634	core	Hypothetical protein	Shared	R			x	x
llmg_1455	LLNZ_07500	NCDO2118_RS02380	L22116	core	6-phospho-beta-glucosidase	Shared	S			x	x
llmg_0916	LLNZ_04705	NCDO2118_RS08405	L29171	core	UDP-N-acetylglucosamine 1-carboxyvinyltransferase	Shared	M			x	x
llmg_0245	LLNZ_01270	NCDO2118_RS01175	L33417	core	Hypothetical protein	Shared	G			x	x
llmg_1978	LLNZ_10215	NCDO2118_RS09860	L37916	core	Glutamate ABC transporter ATP-binding protein	Shared	G			x	x
llmg_0415	LLNZ_02165	NCDO2118_RS02285	L3846	core	Thymidylate kinase	Shared	S			x	x
llmg_0455	LLNZ_02355	NCDO2118_RS02445	L39593	core	Trehalose 6-phosphate phosphorylase	Shared	IR			x	x
llmg_0035	LLNZ_00170	NCDO2118_RS12105	L41144	core	Hypothetical protein	Shared	S			x	x
llmg_0252	LLNZ_01305	NCDO2118_RS01230	L41779	core	Hydrolase	Shared	X		x	x	
llmg_0044	LLNZ_00215	NCDO2118_RS12055	L45035	core	Hypothetical protein	Shared	F			x	x
llmg_1731	LLNZ_08940	NCDO2118_RS04325	L45109	core	Transcriptional regulator	Shared	K			x	x
llmg_1730	LLNZ_08935	NCDO2118_RS04330	L45583	core	Mercuric reductase	Shared	S			x	x
llmg_0797	LLNZ_04130	NCDO2118_RS04935	L52331	core	Antirepressor	Shared	S			x	x
llmg_0267	LLNZ_01400	NCDO2118_RS01315	L58584	core	Hypothetical protein	Shared	J			x	x
llmg_1993	LLNZ_10285	NCDO2118_RS09930	L58790	core	Amino acid permease	Shared	G			x	x
llmg_1945	LLNZ_10015	NCDO2118_RS09690	L5953	core	ATP synthase epsilon chain	Shared	S		x	x	x
llmg_0269	LLNZ_01410	NCDO2118_RS01335	L61680	core	Multidrug ABC transporter ATP-binding protein	Shared	M			x	x

llmg_1997	LLNZ_10305	NCDO2118_RS09950	L63811	core	Pyruvate formate lyase-activating protein	Shared	R		x	x
llmg_2481	LLNZ_12820	NCDO2118_RS12025	L64445	core	Hypothetical protein	Shared	H		x	x
llmg_1058	LLNZ_05470	NCDO2118_RS07520	L66222	core	Pantothenate kinase	Shared	S		x	x
llmg_2485	LLNZ_12840	NCDO2118_RS12175	L67158	core	Glutamine synthetase	Shared	S		x	x
llmg_2009	LLNZ_10355	NCDO2118_RS10085	L71879	core	Hypothetical protein	Shared	G		x	x
llmg_2490	LLNZ_12865	NCDO2118_RS12220	L75803	core	Hypothetical protein	Shared	P	x	x	x
llmg_0420	LLNZ_02190	NCDO2118_RS02310	L7612	core	Hypothetical protein	Shared	P		x	x
llmg_0288	LLNZ_01515	NCDO2118_RS01670	L76755	core	Cobalt ABC transporter ATP-binding protein	Shared	H		x	x
llmg_2014	LLNZ_10380	NCDO2118_RS10115	L76848	core	Hypothetical protein	Shared	P		x	x
llmg_1687	LLNZ_08700	NCDO2118_RS04490	L81441	core	Cell division protein FtsJ	Shared	S		x	x
llmg_0293	LLNZ_01540	NCDO2118_RS01695	L81453	core	1,4-beta-xylanase	Shared	D		x	x
llmg_2498	LLNZ_12905	NCDO2118_RS12260	L84494	core	Hypothetical protein	Shared	T	x	x	x
llmg_1495	LLNZ_07695	NCDO2118_RS05630	L88637	core	Hypothetical protein	Shared	J		x	x
llmg_1221	LLNZ_06290	NCDO2118_RS06695	L90005	core	D-alanine--poly(phosphoribitol) ligase subunit 2	Shared	C		x	x
llmg_1492	LLNZ_07680	NCDO2118_RS05645	L90693	core	Ribosomal protein	Shared	G	x	x	
llmg_1879	LLNZ_09680	NCDO2118_RS03670	L92295	core	Hypothetical protein	Shared	E		x	x
llmg_1487	LLNZ_07655	NCDO2118_RS05670	L94534	core	Endoribonuclease YbeY	Shared	G	x	x	x
llmg_0310	LLNZ_01620	NCDO2118_RS01765	L94552	core	Hypothetical protein	Shared	J		x	x
llmg_2311	LLNZ_11935	NCDO2118_RS11165	L94890	core	Arginine/ornitine antiporter	Shared	H		x	x
llmg_2510	LLNZ_12965	NCDO2118_RS12315	L99869	core	NUDIX hydrolase	Shared	F		x	x
llmg_1871	LLNZ_09635	NCDO2118_RS03700	L99884	core	Glycogen phosphorylase	Shared	R		x	x
llmg_0697	LLNZ_03625	NCDO2118_RS10190	L93148	core	oligopeptide transport ATP-binding protein oppD	Exclusive	E	x		
llmg_1279	LLNZ_06600	NCDO2118_RS06425	L0078	core	acetolactate synthase catalytic subunit	Exclusive	E	x		
llmg_0745	LLNZ_03880	NCDO2118_RS09205	L128690	core	maltose phosphorylase	Exclusive	G	x		
llmg_0766	LLNZ_03985	NCDO2118_RS09090	L0211	core	DNA translocase ftsK	Exclusive	J	x		
llmg_0322	LLNZ_01695	NCDO2118_RS01825	L106965	core	cation transporter	Exclusive	P	x		
llmg_1737	LLNZ_08970	NCDO2118_RS04295	L34494	core	non-heme chloride peroxidase	Exclusive	P	x		
llmg_0880	LLNZ_04530	NCDO2118_RS08860	L55584	core	putative oxidoreductase	Exclusive	R	x		
llmg_1618	LLNZ_08325	NCDO2118_RS04820	L127902	core	Hypothetical protein	Exclusive	S	x		
llmg_2163	LLNZ_11155	NCDO2118_RS10510	L157237	core	Hypothetical protein	Exclusive	S	x		
llmg_2465	LLNZ_12740	NCDO2118_RS11925	L39650	core	Hypothetical protein	Exclusive	S	x		
llmg_2500	LLNZ_12915	NCDO2118_RS12270	L86471	core	Hypothetical protein	Exclusive	S	x		
llmg_1807	LLNZ_09305	NCDO2118_RS03970	L159954	core	Nitroreductase	Exclusive	C		x	
llmg_1991	LLNZ_10275	NCDO2118_RS09920	L55758	core	Alcohol dehydrogenase	Exclusive	C		x	
llmg_1733	LLNZ_08950	NCDO2118_RS04315	L43452	core	Serine dehydratase subunit beta	Exclusive	E		x	
llmg_1928	LLNZ_09925	NCDO2118_RS09605	L0132	core	Histidine kinase	Exclusive	E		x	
llmg_2310	LLNZ_11930	NCDO2118_RS11160	L93826	core	Carbamate kinase	Exclusive	E		x	
llmg_2562	LLNZ_13245	NCDO2118_RS12525	L141634	core	6-phosphogluconate dehydrogenase	Exclusive	G		x	
llmg_0994	LLNZ_05120	NCDO2118_RS07950	L158710	core	Purine biosynthesis protein	Exclusive	F		x	
llmg_1023	LLNZ_05280	NCDO2118_RS07670	L105533	core	Sugar ABC transporter substrate-binding protein	Exclusive	G		x	
llmg_1320	LLNZ_06820	NCDO2118_RS07890	L0234	core	Beta-xylosidase	Exclusive	G		x	
llmg_1430	LLNZ_07370	NCDO2118_RS05870	L0167	core	Riboflavin biosynthesis protein RibF	Exclusive	H		x	
llmg_0012	LLNZ_00060	NCDO2118_RS00065	L0365	core	Peptidyl-tRNA hydrolase	Exclusive	J		x	
llmg_1753	LLNZ_09055	NCDO2118_RS04215	L0326	core	Ribonuclease 3	Exclusive	J		x	
llmg_0023	LLNZ_00110	NCDO2118_RS00160	L30901	core	Transcriptional antiterminator	Exclusive	K		x	
llmg_1256	LLNZ_06470	NCDO2118_RS01420	L117090	core	Cold-shock protein	Exclusive	K		x	
llmg_0514	LLNZ_02655	NCDO2118_RS02665	L132585	core	Regulatory protein Spx 1	Exclusive	K		x	
llmg_1156	LLNZ_05965	NCDO2118_RS07035	L145375	core	Arsenical resistance operon trans-acting repressor	Exclusive	K		x	
llmg_0754	LLNZ_03925	NCDO2118_RS09160	L116756	core	XRE family transcriptional regulator	Exclusive	K		x	
llmg_0201	LLNZ_01050	NCDO2118_RS00950	L192996	core	Methionine sulfoxide reductase B	Exclusive	L		x	
llmg_1502	LLNZ_07730	NCDO2118_RS05595	L0285	core	DNA replication protein DnaD	Exclusive	L		x	
llmg_0955	LLNZ_04905	NCDO2118_RS08105	L190278	core	Molecular chaperone GroES	Exclusive	O		x	
llmg_pseudo	LLNZ_03020	NCDO2118_RS03210	L9458	core	Potassium transporter kup	Exclusive	P		x	
llmg_1115	LLNZ_05765	NCDO2118_RS07210	L175712	core	5-bromo-4-chloroindolyl phosphate hydrolase	Exclusive	Q		x	
llmg_2325	LLNZ_12010	NCDO2118_RS10745	L16252	core	Pyridoxamine 5'-phosphate oxidase	Exclusive	Q		x	
llmg_0083	LLNZ_00410	NCDO2118_RS00270	L71174	core	Phosphate acyltransferase	Exclusive	R		x	
llmg_0091	LLNZ_00455	NCDO2118_RS00300	L75975	core	O-acetylhomoserine aminocarboxypropyltransferase	Exclusive	R		x	
llmg_0594	LLNZ_03050	NCDO2118_RS01645	L47248	core	Integrase	Exclusive	R		x	
llmg_0459	LLNZ_02375	NCDO2118_RS02470	L44542	core	Hydrolase	Exclusive	R		x	
llmg_1639	LLNZ_08435	NCDO2118_RS04725	L119564	core	ABC-F type ribosomal protection protein	Exclusive	R		x	
llmg_0970	LLNZ_04985	NCDO2118_RS08040	L178933	core	4-carboxymuconolactone decarboxylase	Exclusive	R		x	
llmg_0950	LLNZ_04880	NCDO2118_RS08130	L194226	core	Oxidoreductase	Exclusive	R		x	
llmg_2134	LLNZ_11005	NCDO2118_RS08745	L51420	core	antirepressor	Exclusive	R		x	
llmg_2395	LLNZ_12370	NCDO2118_RS11575	L164222	core	FMN-binding protein	Exclusive	R		x	
llmg_2436	LLNZ_12590	NCDO2118_RS11805	L16911	core	Radical SAM protein	Exclusive	R		x	
llmg_2458	LLNZ_12705	NCDO2118_RS11895	L34612	core	fibronectin-binding protein	Exclusive	R		x	
llmg_0159	LLNZ_00835	NCDO2118_RS00665	L148007	core	Hypothetical protein	Exclusive	S		x	
llmg_0186	LLNZ_00970	NCDO2118_RS00860	L176316	core	Hypothetical protein	Exclusive	S		x	
llmg_1816	LLNZ_09350	NCDO2118_RS03920	L151062	core	Hypothetical protein	Exclusive	S		x	
llmg_2216	LLNZ_11430	NCDO2118_RS10800	L25433	core	Hypothetical protein	Exclusive	S		x	
llmg_2019	LLNZ_10405	NCDO2118_RS10135	L0098	core	Aspartate aminotransferase	Exclusive	E			x
llmg_0987	LLNZ_05080	NCDO2118_RS07980	L165202	core	Phosphoribosylaminoimidazole synthetase	Exclusive	F			x
llmg_1333	LLNZ_06895	NCDO2118_RS06025	L169971	core	Deoxynucleoside kinase	Exclusive	F			x
llmg_1623	LLNZ_08350	NCDO2118_RS04795	L137446	core	Sugar ABC transporter ATP-binding protein	Exclusive	G			x
llmg_2205	LLNZ_11380	NCDO2118_RS10765	L18647	core	Dihydrofolate reductase	Exclusive	H			x
llmg_0632	LLNZ_03265	NCDO2118_RS03345	L0427	core	50S ribosomal protein L33	Exclusive	J			x
llmg_2549	LLNZ_13170	NCDO2118_RS12485	L132875	core	Ribonuclease HIII	Exclusive	L			x
llmg_0873	LLNZ_04490	NCDO2118_RS08895	L65733	core	DNA mismatch repair protein MutT	Exclusive	L			x
llmg_0312	LLNZ_01630	NCDO2118_RS01780	L96217	core	Phosphonate ABC transporter substrate-binding protein	Exclusive	P			x
llmg_1832	LLNZ_09425	NCDO2118_RS03840	L0172	core	2-succinylbenzoate--CoA ligase	Exclusive	Q			x
llmg_0117	LLNZ_00585	NCDO2118_RS00420	L111950	core	Hydrolase	Exclusive	R			x
llmg_0238	LLNZ_01230	NCDO2118_RS01135	L27646	core	GNAT family acetyltransferase	Exclusive	R			x
llmg_0767	LLNZ_03990	NCDO2118_RS09085	L103502	core	Hypothetical protein	Exclusive	S			x
llmg_2340	LLNZ_12095	NCDO2118_RS11300	L128737	core	Hypothetical protein	Exclusive	S			x
llmg_2091	LLNZ_10755	NCDO2118_RS08545	L31473	core	Phage-related Tail protein	Exclusive	X			x
llmg_0456	LLNZ_02360	NCDO2118_RS02450	L0001	core	Beta-phosphoglucomutase	None	G			
llmg_1098	LLNZ_05680	NCDO2118_RS06625	L0013	core	GlpD protein	None	C			
llmg_1097	LLNZ_05675	NCDO2118_RS06620	L0015	core	Glycerol uptake facilitator	None	G			
llmg_0392	LLNZ_02045	NCDO2118_RS02175	L0018	core	L-lactate dehydrogenase 2 (L-LDH 2)	None	C			
llmg_0862	LLNZ_04430	NCDO2118_RS08945	L0019	core	Uronate isomerase	None	G			
llmg_0864	LLNZ_04445	NCDO2118_RS08935	L0022	core	2-dehydro-3-deoxyphosphogluconate aldolase / 4-hydroxy-2-oxoglutara	None	G			
llmg_0860	LLNZ_04420	NCDO2118_RS08955	L0023	core	Na-galactoside symporter	None	G			
llmg_1570	LLNZ_08085	NCDO2118_RS05330	L0031	core	Transcriptional regulator of the fructose operon	None	KG			
llmg_1042	LLNZ_05375	NCDO2118_RS07600	L0048	core	Tryptophan synthase alpha chain	None	E			
llmg_1041	LLNZ_05370	NCDO2118_RS07605	L0049	core	Tryptophan synthase beta chain	None	E			
llmg_1039	LLNZ_05360	NCDO2118_RS07615	L0050	core	N-(5'-phosphoribosyl)anthranilate isomerase (PRAI)	None	E			
llmg_1032	LLNZ_05325	NCDO2118_RS07625	L0052	core	Anthranilate phosphoribosyltransferase	None	E			
llmg_1031	LLNZ_05320	NCDO2118_RS07630	L0053	core	Anthranilate synthase component II	None	EH			
llmg_1030	LLNZ_05315	NCDO2118_RS07635	L0054	core	Anthranilate synthase component I	None	EH			
llmg_1924	LLNZ_09905	NCDO2118_RS09585	L0055	core	Prephenate dehydratase (PDT)	None	E			
llmg_1925	LLNZ_09910	NCDO2118_RS09590	L0056	core	Shikimate kinase (SK)	None	E			
llmg_1939	LLNZ_09990	NCDO2118_RS09660	L0061	core	Shikimate dehydrogenase (NADP(+)) (SDH)	None	E			
llmg_1298	LLNZ_06710	NCDO2118_RS06325	L0065	core	Histidinol-phosphate aminotransferase	None	E			
llmg_1296	LLNZ_06695	NCDO2118_RS06335	L0066	core	ATP phosphoribosyltransferase	None	E			
llmg_1295	LLNZ_06690	NCDO2118_RS06340	L0067	core	Histidinol dehydrogenase	None	E			
llmg_1294	LLNZ_06685	NCDO2118_RS06350	L0068	core	Imidazoleglycerol-phosphate dehydratase	None	E			
llmg_1292	LLNZ_06675	NCDO2118_RS06360	L0069	core	Imidazole glycerol phosphate synthase subunit HisH	None	E			
llmg_1290	LLNZ_06665	NCDO2118_RS06370	L0071	core	Imidazole glycerol phosphate synthase subunit HisF	None	E			

llmg_1289	LLNZ_06660	NCDO2118_RS06375	L0072	core	Histidine biosynthesis bifunctional protein HisIE	None	E
llmg_1284	LLNZ_06625	NCDO2118_RS06405	L0075	core	3-isopropylmalate dehydratase large subunit	None	E
llmg_1282	LLNZ_06615	NCDO2118_RS06410	L0076	core	3-isopropylmalate dehydratase small subunit	None	E
llmg_1280	LLNZ_06605	NCDO2118_RS06420	L0077	core	Dihydroxy-acid dehydratase	None	EG
llmg_1278	LLNZ_06595	NCDO2118_RS06430	L0079	core	Acetolactate synthase small subunit	None	E
llmg_1277	LLNZ_06590	NCDO2118_RS06435	L0080	core	Ketol-acid reductoisomerase	None	EH
llmg_1276	LLNZ_06585	NCDO2118_RS06440	L0081	core	L-threonine dehydratase	None	E
llmg_0565	LLNZ_02900	NCDO2118_RS03040	L0083	core	Phosphoserine aminotransferase	None	HE
llmg_0567	LLNZ_02910	NCDO2118_RS03050	L0085	core	SerB protein	None	E
llmg_1226	LLNZ_06315	NCDO2118_RS06670	L0099	core	Methylenetetrahydrofolate reductase	None	E
llmg_1225	LLNZ_06310	NCDO2118_RS06675	L0100	core	5-methyltetrahydropteroyltriglutamate--homocysteine methyltransfera	None	E
llmg_2182	LLNZ_11265	NCDO2118_RS10635	L0101	core	Homoserine O-succinyltransferase	None	E
llmg_2181	LLNZ_11260	NCDO2118_RS10630	L0102	core	Cystathionine gamma-synthase	None	E
llmg_1758	LLNZ_09080	NCDO2118_RS04190	L0104	core	N-acetyl-gamma-glutamyl-phosphate reductase	None	E
llmg_1757	LLNZ_09075	NCDO2118_RS04195	L0105	core	Arginine biosynthesis bifunctional protein	None	E
llmg_1755	LLNZ_09065	NCDO2118_RS04205	L0107	core	Acetylglutamate kinase	None	E
llmg_1754	LLNZ_09060	NCDO2118_RS04210	L0108	core	Ornithine carbamoyltransferase, catabolic	None	E
llmg_2307	LLNZ_11915	NCDO2118_RS11145	L0111	core	Arginine/ornithine antiporter	None	E
llmg_0536	LLNZ_02765	NCDO2118_RS02845	L0115	core	Acetylornithine deacetylase	None	E
llmg_1183	LLNZ_06090	NCDO2118_RS06835	L0119	core	Glutamate synthase, large subunit	None	E
llmg_1649	LLNZ_08485	NCDO2118_RS04680	L0126	core	Sensor histidine kinase	None	T
llmg_1518	LLNZ_07805	NCDO2118_RS05525	L0128	core	Sensor kinase protein	None	T
llmg_1520	LLNZ_07815	NCDO2118_RS05515	L0129	core	Two-component system regulator	None	TK
llmg_0909	LLNZ_04670	NCDO2118_RS08440	L0130	core	Sensor protein kinase kinA	None	T
llmg_1929	LLNZ_09930	NCDO2118_RS09610	L0133	core	Two-component system regulator	None	TK
llmg_0748	LLNZ_03895	NCDO2118_RS09190	L0134	core	Sensor protein kinase kinF	None	T
llmg_pseudo	LLNZ_12650	NCDO2118_RS11855	L0142	core	RNA polymerase ECF sigma factor	None	K
llmg_0784	LLNZ_04065	NCDO2118_RS09005	L0145	core	Ribose operon repressor	None	K
llmg_1674	LLNZ_08625	NCDO2118_RS04560	L0148	core	Malolactic fermentation system transcriptional activator	None	K
llmg_0499	LLNZ_02580	NCDO2118_RS02595	L0149	core	Methylphosphotriester-DNA alkyltransferase	None	F
llmg_1767	LLNZ_09125	NCDO2118_RS04150	L0152	core	Transcriptional regulator, DeoR family	None	KG
llmg_1172	LLNZ_06035	NCDO2118_RS06890	L0153	core	Transcriptional regulator	None	KG
llmg_1532	LLNZ_07880	NCDO2118_RS05470	L0163	core	Riboflavin biosynthesis protein RibD	None	H
llmg_1531	LLNZ_07875	NCDO2118_RS05475	L0164	core	RibB protein	None	H
llmg_1530	LLNZ_07870	NCDO2118_RS05480	L0165	core	Riboflavin biosynthesis protein RibBA	None	H
llmg_1529	LLNZ_07865	NCDO2118_RS05485	L0166	core	6,7-dimethyl-8-ribityllumazine synthase	None	H
llmg_1828	LLNZ_09405	NCDO2118_RS03860	L0168	core	Menaquinone-specific isochorismate synthase	None	HQ
llmg_1830	LLNZ_09415	NCDO2118_RS03850	L0170	core	2-succinyl-6-hydroxy-2,4-cyclohexadiene-1-carboxylate synthase	None	Q
llmg_1337	LLNZ_06915	NCDO2118_RS06005	L0175	core	Bifunctional protein FolKE	None	H
llmg_1153	LLNZ_05950	NCDO2118_RS07050	L0178	core	PabA (p-aminobenzoate synthetase, component II)	None	EH
llmg_1154	LLNZ_05955	NCDO2118_RS07045	L0179	core	Para-aminobenzoate synthase component I	None	EH
llmg_1963	LLNZ_10120	NCDO2118_RS09780	L0191	core	Bifunctional ligase/repressor BirA	None	H
llmg_2162	LLNZ_11150	NCDO2118_RS10505	L0192	core	Acetyl-CoA carboxylase ligase / biotin operon repressor bifunctional prc	None	H
llmg_1418	LLNZ_07310	NCDO2118_RS05925	L0193	core	Coproporphyrinogen III oxidase	None	H
llmg_0934	LLNZ_04805	NCDO2118_RS08205	L0194	core	Ferrochelatase	None	H
llmg_1088	LLNZ_05630	NCDO2118_RS07335	L0198	core	Glutathione peroxidase	None	O
llmg_1217	LLNZ_06270	NCDO2118_RS06720	L0200	core	Phosphomethylpyrimidine kinase	None	H
llmg_1218	LLNZ_06275	NCDO2118_RS06715	L0201	core	Thiamine-phosphate synthase	None	H
llmg_2067	LLNZ_10640	NCDO2118_RS10365	L0215	core	Transcriptional regulator, LysR family	None	K
llmg_0393	LLNZ_02050	NCDO2118_RS02180	L0217	core	Transcriptional regulator, LysR family	None	K
llmg_0390	LLNZ_02035	NCDO2118_RS02165	L0220	core	Transcriptional regulator, LysR family	None	K
llmg_1001	LLNZ_05165	NCDO2118_RS07915	L0229	core	Xylose operon regulator	None	K
llmg_1002	LLNZ_05170	NCDO2118_RS07910	L0230	core	Xylose isomerase	None	G
llmg_0910	LLNZ_04675	NCDO2118_RS08435	L0236	core	Ammonium transporter	None	P
llmg_0858	LLNZ_04410	NCDO2118_RS08965	L0241	core	Fructuronate reductase	None	G
llmg_1983	LLNZ_10240	NCDO2118_RS09885	L0244	core	Transcriptional regulator, CodY family	None	K
llmg_2068	LLNZ_10645	NCDO2118_RS10370	L0246	core	Transcriptional regulator, MerR family	None	K
llmg_1020	LLNZ_05260	NCDO2118_RS07680	L0247	core	Transcriptional regulator, MerR family	None	K
llmg_0004	LLNZ_00020	NCDO2118_RS00020	L0251	core	ATP-dependent helicase/nuclease subunit A	None	L
llmg_1501	LLNZ_07725	NCDO2118_RS05600	L0253	core	Endonuclease III	None	L
llmg_2523	LLNZ_13025	NCDO2118_RS12375	L0262	core	DNA helicase RecG	None	LK
llmg_0013	LLNZ_00065	NCDO2118_RS00070	L0263	core	Transcription-repair-coupling factor	None	LK
llmg_1992	LLNZ_10280	NCDO2118_RS09925	L0268	core	DNA helicase RecQ	None	L
llmg_2491	LLNZ_12870	NCDO2118_RS12225	L0277	core	DNA mismatch repair protein MutS	None	L
llmg_1444	LLNZ_07445	NCDO2118_RS05840	L0288	core	ADP-ribose pyrophosphatase	None	LR
llmg_2153	LLNZ_11105	NCDO2118_RS10470	L0292	core	Primosomal protein N'	None	L
llmg_1717	LLNZ_08855	NCDO2118_RS04375	L0296	core	A/G-specific adenine glycosylase	None	L
llmg_0409	LLNZ_02130	NCDO2118_RS02255	L0299	core	Single-stranded DNA-binding protein	None	L
llmg_2305	LLNZ_11905	NCDO2118_RS11135	L0305	core	DNA polymerase IV	None	L
llmg_1515	LLNZ_07790	NCDO2118_RS05540	L0306	core	UPF0758 protein	None	L
llmg_0483	LLNZ_02500	NCDO2118_RS02550	L0307	core	DNA-directed DNA polymerase	None	L
llmg_2408	LLNZ_12445	NCDO2118_RS11635	L0313	core	Putative competence protein ComGA	None	NU
llmg_2407	LLNZ_12440	NCDO2118_RS11630	L0314	core	Putative competence protein ComGB	None	NU
llmg_2406	LLNZ_12435	NCDO2118_RS11625	L0315	core	Putative competence protein ComGC	None	U
llmg_1955	LLNZ_10075	NCDO2118_RS09740	L0316	core	Putative competence protein	None	L
llmg_1483	LLNZ_07635	NCDO2118_RS05690	L0318	core	Superfamily II DNA/RNA helicase required for DNA uptake	None	L
llmg_1132	LLNZ_05845	NCDO2118_RS07130	L0327	core	Nuclease SbcCD subunit D	None	L
llmg_2304	LLNZ_11900	NCDO2118_RS11130	L0332	core	Type 4 prepilin-like protein specific leader peptidase	None	R
llmg_2361	LLNZ_12200	NCDO2118_RS11410	L0333	core	Protein translocase subunit SecY	None	U
llmg_1525	LLNZ_07840	NCDO2118_RS05490	L0335	core	Lipoprotein signal peptidase	None	MU
llmg_1587	LLNZ_08170	NCDO2118_RS05250	L0338	core	Protein-export membrane protein secG	None	U
llmg_1297	LLNZ_06705	NCDO2118_RS06330	L0341	core	ATP phosphoribosyltransferase regulatory subunit	None	E
llmg_2370	LLNZ_12245	NCDO2118_RS11450	L0391	core	30S ribosomal protein S14 type Z	None	J
llmg_0251	LLNZ_01300	NCDO2118_RS01195	L0398	core	30S ribosomal protein S21	None	J
llmg_2030	LLNZ_10465	NCDO2118_RS10200	L0430	core	50S ribosomal protein L35	None	J
llmg_0654	LLNZ_03385	NCDO2118_RS06515	L0438	core	Transposase for insertion sequence element IS-LL6B	None	J
llmg_1666	LLNZ_08575	NCDO2118_RS04585	L100099	core	Hypothetical protein	None	S
llmg_0015	LLNZ_00070	NCDO2118_RS00080	L1001	core	Putative RNA binding protein	None	S
llmg_0427	LLNZ_02225	NCDO2118_RS02325	L10014	core	Phosphomevalonate kinase	None	J
llmg_0019	LLNZ_00090	NCDO2118_RS00100	L1002	core	tRNA(Ile)-lysidine synthase	None	I
llmg_0063	LLNZ_00315	NCDO2118_RS00190	L1004	core	Sulphate transporter	None	G
llmg_1479	LLNZ_07615	NCDO2118_RS05710	L100541	core	5'-nucleotidase	None	Q
llmg_0104	LLNZ_00520	NCDO2118_RS00360	L100575	core	Acetyltransferase, GNAT family	None	R
llmg_0067	LLNZ_00335	NCDO2118_RS00210	L1006	core	DNA repair protein RecO	None	R
llmg_0077	LLNZ_00380	NCDO2118_RS00245	L1008	core	Putative phosphohydrolase	None	S
llmg_1664	LLNZ_08565	NCDO2118_RS04590	L100822	core	Nicotinamide mononucleotide transporter	None	S
llmg_0332	LLNZ_01745	NCDO2118_RS01885	L1011	core	Biotin transporter BioY2	None	S
llmg_2039	LLNZ_10505	NCDO2118_RS10235	L101219	core	Mini-ribonuclease 3	None	S
llmg_1827	LLNZ_09400	NCDO2118_RS03865	L1014	core	Putative acetyltransferase	None	S
llmg_1766	LLNZ_09120	NCDO2118_RS04155	L1015	core	Putative acetyltransferase	None	J
llmg_0495	LLNZ_02560	NCDO2118_RS02575	L101577	core	Hypothetical protein	None	J
llmg_1029	LLNZ_05310	NCDO2118_RS07640	L101688	core	Hypothetical protein	None	M
llmg_2513	LLNZ_12980	NCDO2118_RS12330	L101912	core	Transport protein	None	U
llmg_1869	LLNZ_09630	NCDO2118_RS03705	L102412	core	Amylopullulanase	None	E
llmg_1661	LLNZ_08550	NCDO2118_RS04600	L102634	core	HU-like DNA-binding protein	None	L
llmg_0790	LLNZ_04095	NCDO2118_RS04895	L102735	core	Putative integrase	None	L
llmg_0006	LLNZ_00030	NCDO2118_RS00030	L10283	core	HTH-type transcriptional regulator	None	L
llmg_2041	LLNZ_10515	NCDO2118_RS10245	L102972	core	Hypothetical protein	None	K

llmg_1660	LLNZ_08545	NCDO2118_RS04605	L103086	core	Transcriptional regulator, TetR family	None	U
llmg_1026	LLNZ_05295	NCDO2118_RS07655	L103246	core	Putative methyltransferase	None	S
llmg_0109	LLNZ_00545	NCDO2118_RS00380	L103661	core	Hypothetical protein	None	S
llmg_1659	LLNZ_08540	NCDO2118_RS04610	L103741	core	Hypothetical protein	None	S
llmg_1865	LLNZ_09605	NCDO2118_RS03710	L104437	core	Di-/tripeptide transporter	None	H
llmg_1202	LLNZ_06185	NCDO2118_RS06770	L104789	core	ABC transporter ABC binding and permease protein	None	S
llmg_0320	LLNZ_01685	NCDO2118_RS01815	L104969	core	Multidrug-efflux transporter protein	None	U
llmg_1474	LLNZ_07595	NCDO2118_RS05730	L105113	core	Putative voltage gated chloride channel	None	V
llmg_0321	LLNZ_01690	NCDO2118_RS01820	L106489	core	Transcriptional regulator, MarR family	None	R
llmg_1021	LLNZ_05265	NCDO2118_RS07675	L106608	core	Hypothetical protein	None	K
llmg_1195	LLNZ_06150	NCDO2118_RS06775	L106755	core	Riboflavin transporter RibU	None	R
llmg_0764	LLNZ_03975	NCDO2118_RS09100	L107270	core	Hypothetical protein	None	P
llmg_1864	LLNZ_09600	NCDO2118_RS03715	L107726	core	Cytochrome bd-I ubiquinol oxidase subunit I	None	J
llmg_1019	LLNZ_05255	NCDO2118_RS07685	L108008	core	Oxidoreductase, short-chain dehydrogenase/reductase family protein	None	S
llmg_2517	LLNZ_13000	NCDO2118_RS12350	L108801	core	Hypothetical protein	None	K
llmg_1863	LLNZ_09595	NCDO2118_RS03720	L109201	core	Cytochrome d ubiquinol oxidase, subunit II	None	V
llmg_0114	LLNZ_00570	NCDO2118_RS00405	L109453	core	Glycosyl transferase, family 2	None	F
llmg_1468	LLNZ_07565	NCDO2118_RS05760	L110431	core	ABC transporter ATP binding protein	None	S
llmg_1190	LLNZ_06125	NCDO2118_RS06800	L110441	core	Hypothetical protein	None	U
llmg_1862	LLNZ_09590	NCDO2118_RS03725	L110479	core	Cytochrome D ABC transporter ATP binding and permease protein	None	T
llmg_0115	LLNZ_00575	NCDO2118_RS00410	L110564	core	D-aminoacyl-tRNA deacylase	None	CO
llmg_1650	LLNZ_08490	NCDO2118_RS04675	L110588	core	Hypothetical protein	None	J
llmg_0116	LLNZ_00580	NCDO2118_RS00415	L111003	core	Hypothetical protein	None	K
llmg_0760	LLNZ_03955	NCDO2118_RS09125	L111159	core	Putative transglycosylase	None	P
llmg_1467	LLNZ_07560	NCDO2118_RS05765	L111162	core	ABC transporter ATP binding protein	None	S
llmg_1016	LLNZ_05235	NCDO2118_RS07725	L111525	core	Cationic transporter	None	S
llmg_1861	LLNZ_09585	NCDO2118_RS03730	L112352	core	Cytochrome D ABC transporter ATP binding and permease protein	None	S
llmg_0759	LLNZ_03950	NCDO2118_RS09130	L112353	core	Putative prenyltransferase, UbiA family	None	CO
llmg_1015	LLNZ_05230	NCDO2118_RS07730	L112499	core	Hypothetical protein	None	H
llmg_2522	LLNZ_13020	NCDO2118_RS12370	L112616	core	Hypothetical protein	None	S
llmg_0118	LLNZ_00590	NCDO2118_RS00425	L112776	core	Amino-acid transporter	None	S
llmg_0218	LLNZ_01135	NCDO2118_RS01045	L11285	core	Putative glycosyl transferase	None	E
llmg_0758	LLNZ_03945	NCDO2118_RS09135	L113400	core	Chloride channel protein	None	M
llmg_2322	LLNZ_11990	NCDO2118_RS11225	L114054	core	Hypothetical protein	None	Q
llmg_1645	LLNZ_08465	NCDO2118_RS04700	L114402	core	General stress protein GSP13	None	K
llmg_2200	LLNZ_11355	NCDO2118_RS10715	L11456	core	Chitin binding protein, putative	None	V
llmg_0331	LLNZ_01740	NCDO2118_RS01880	L114632	core	Hypothetical protein	None	I
llmg_1184	LLNZ_06095	NCDO2118_RS06830	L114827	core	Glutamate synthase, small subunit 1	None	M
llmg_1644	LLNZ_08460	NCDO2118_RS04705	L114884	core	Hypothetical protein	None	ER
llmg_0902	LLNZ_04635	NCDO2118_RS08495	L11493	core	Arsenate reductase	None	S
llmg_0120	LLNZ_00605	NCDO2118_RS00435	L115025	core	Transcriptional regulator, MarR family	None	G
llmg_0757	LLNZ_03940	NCDO2118_RS09140	L115107	core	Hypothetical protein	None	K
llmg_1859	LLNZ_09570	NCDO2118_RS03740	L115265	core	Flavodoxin	None	S
llmg_0334	LLNZ_01755	NCDO2118_RS01895	L116212	core	Thiamine transporter ThiT	None	F
llmg_2326	LLNZ_12015	NCDO2118_RS11235	L116216	core	Hypothetical protein	None	S
llmg_0755	LLNZ_03930	NCDO2118_RS09150	L116299	core	Hypothetical protein	None	R
llmg_1642	LLNZ_08450	NCDO2118_RS04715	L117145	core	2,3-butanediol dehydrogenase	None	K
llmg_1951	LLNZ_10045	NCDO2118_RS09720	L11729	core	ATP synthase subunit A	None	S
llmg_1006	LLNZ_05190	NCDO2118_RS07770	L117718	core	Fructokinase	None	D
llmg_2541	LLNZ_13125	NCDO2118_RS12395	L117821	core	Cation transporting ATPase	None	S
llmg_0498	LLNZ_02575	NCDO2118_RS02590	L118481	core	6-O-methylguanine-DNA methyltransferase	None	M
llmg_1005	LLNZ_05185	NCDO2118_RS07775	L118696	core	Sugar kinase and transcriptional regulator	None	D
llmg_1854	LLNZ_09540	NCDO2118_RS03760	L119032	core	Alkaline phosphatase-like protein	None	R
llmg_0338	LLNZ_01770	NCDO2118_RS01910	L119452	core	Lipoprotein	None	S
llmg_2057	LLNZ_10590	NCDO2118_RS10315	L119456	core	Hypothetical protein	None	P
llmg_1004	LLNZ_05180	NCDO2118_RS07780	L119614	core	Beta-glucosidase	None	R
llmg_0501	LLNZ_02590	NCDO2118_RS02600	L119676	core	ABC transporter ATP binding protein	None	G
llmg_0340	LLNZ_01780	NCDO2118_RS01915	L120334	core	Lipoprotein	None	U
llmg_0502	LLNZ_02595	NCDO2118_RS02605	L120437	core	ABC transporter permease protein	None	S
llmg_2542	LLNZ_13130	NCDO2118_RS12400	L120589	core	HTH-type transcriptional regulator	None	G
llmg_1182	LLNZ_06085	NCDO2118_RS06840	L120883	core	Putative acetyltransferase	None	G
llmg_0503	LLNZ_02600	NCDO2118_RS02610	L121252	core	Transcriptional regulator, LytR family	None	S
llmg_1638	LLNZ_08430	NCDO2118_RS04730	L121483	core	Malolactic enzyme	None	G
llmg_0504	LLNZ_02605	NCDO2118_RS02615	L121731	core	Hypothetical protein	None	C
llmg_1852	LLNZ_09530	NCDO2118_RS03770	L121994	core	Hypothetical protein	None	F
llmg_0505	LLNZ_02610	NCDO2118_RS02620	L122222	core	Pyridoxal-phosphate dependent aminotransferase	None	F
llmg_1851	LLNZ_09525	NCDO2118_RS03775	L122299	core	Hypothetical protein	None	P
llmg_0342	LLNZ_01790	NCDO2118_RS01925	L122401	core	Amino acid ABC transporter permease protein	None	S
llmg_0750	LLNZ_03905	NCDO2118_RS09180	L122982	core	Putative membrane protein insertion efficiency factor	None	S
llmg_1850	LLNZ_09520	NCDO2118_RS03780	L123116	core	Quinone oxidoreductase	None	S
llmg_0343	LLNZ_01795	NCDO2118_RS01930	L123147	core	UPF0397 protein	None	CR
llmg_1637	LLNZ_08425	NCDO2118_RS04735	L123159	core	Malate transporter	None	S
llmg_2544	LLNZ_13140	NCDO2118_RS12410	L123249	core	Hypothetical protein	None	C
llmg_0008	LLNZ_00040	NCDO2118_RS00040	L12334	core	HTH-type transcriptional regulator	None	R
llmg_0506	LLNZ_02615	NCDO2118_RS02625	L123471	core	Hypothetical protein	None	I
llmg_0130	LLNZ_00670	NCDO2118_RS00480	L123536	core	Hypothetical protein	None	M
llmg_0132	LLNZ_00680	NCDO2118_RS00485	L124677	core	Small Multidrug Resistance protein	None	J
llmg_1636	LLNZ_08420	NCDO2118_RS04740	L124727	core	Putative permease protein	None	P
llmg_1178	LLNZ_06065	NCDO2118_RS06860	L125001	core	Glutamate/gamma-aminobutyrate antiporter	None	R
llmg_0133	LLNZ_00685	NCDO2118_RS00490	L125116	core	Multidrug resistance protein	None	E
llmg_1449	LLNZ_07470	NCDO2118_RS05810	L125196	core	Sortase SrtA	None	V
llmg_2064	LLNZ_10625	NCDO2118_RS10350	L125707	core	Similar to oxidoreductases	None	P
llmg_2335	LLNZ_12070	NCDO2118_RS11275	L125873	core	Hypothetical protein	None	E
llmg_1844	LLNZ_09490	NCDO2118_RS03795	L126240	core	Hypothetical protein	None	I
llmg_1177	LLNZ_06060	NCDO2118_RS06865	L126656	core	Positive regulator GadR	None	T
llmg_0138	LLNZ_00730	NCDO2118_RS00575	L126739	core	Argininosuccinate synthase	None	K
llmg_1632	LLNZ_08400	NCDO2118_RS04750	L126819	core	Putative hydrolase	None	E
llmg_2065	LLNZ_10630	NCDO2118_RS10355	L126956	core	Oxidoreductase, aldo/keto reductase family	None	S
llmg_0509	LLNZ_02630	NCDO2118_RS02640	L127411	core	N-acetylglucosaminidase	None	K
llmg_0347	LLNZ_01820	NCDO2118_RS01955	L127476	core	Ferrichrome ABC transporter permease protein	None	M
llmg_2337	LLNZ_12080	NCDO2118_RS11285	L127611	core	Hypothetical protein	None	P
llmg_2066	LLNZ_10635	NCDO2118_RS10360	L127813	core	Multidrug resistance protein B	None	S
llmg_2338	LLNZ_12085	NCDO2118_RS11290	L127921	core	Hypothetical protein	None	S
llmg_2339	LLNZ_12090	NCDO2118_RS11295	L128255	core	Transcriptional regulator	None	S
llmg_0348	LLNZ_01825	NCDO2118_RS01960	L128386	core	Ferrichrome ABC transporter permease protein	None	S
llmg_0510	LLNZ_02635	NCDO2118_RS02645	L128550	core	Putative Zn-dependent hydrolase of metallo-beta-lactamase superfamil	None	P
llmg_0744	LLNZ_03875	NCDO2118_RS09210	L128691	core	Exo-alpha-1,4-glucosidase	None	G
llmg_0743	LLNZ_03870	NCDO2118_RS09215	L128692	core	Alpha-amylase	None	G
llmg_0741	LLNZ_03860	NCDO2118_RS09225	L128693	core	Oligo-1,6-alpha-glucosidase	None	G
llmg_0740	LLNZ_03855	NCDO2118_RS09230	L128694	core	Neopullulanase	None	G
llmg_0738	LLNZ_03845	NCDO2118_RS09240	L128696	core	Maltose transport system permease protein malF	None	G
llmg_0737	LLNZ_03840	NCDO2118_RS09245	L128697	core	Maltose ABC transporter permease protein malG	None	G
llmg_1448	LLNZ_07465	NCDO2118_RS05820	L129283	core	Hypothetical protein	None	R
llmg_0140	LLNZ_00740	NCDO2118_RS00585	L129379	core	Multidrug resistance efflux pump	None	S
llmg_1174	LLNZ_06045	NCDO2118_RS06880	L130023	core	Hypothetical protein	None	S
llmg_1629	LLNZ_08385	NCDO2118_RS04765	L130150	core	Putative methyltransferase	None	S
llmg_2547	LLNZ_13160	NCDO2118_RS12475	L130944	core	Hypothetical protein	None	K
llmg_1628	LLNZ_08380	NCDO2118_RS04770	L131027	core	Hypothetical protein	None	S

llmg_0512	LLNZ_02645	NCDO2118_RS02655	L131245	core	Holliday junction resolvase RecU	None	S
llmg_1834	LLNZ_09435	NCDO2118_RS03830	L131392	core	Hypothetical protein	None	L
llmg_0351	LLNZ_01840	NCDO2118_RS01975	L131423	core	Arabinose efflux permease	None	Q
llmg_1533	LLNZ_07885	NCDO2118_RS05465	L13157	core	Magnesium and cobalt transport protein	None	V
llmg_0143	LLNZ_00755	NCDO2118_RS00600	L131778	core	Membrane protein insertase YidC	None	P
llmg_1833	LLNZ_09430	NCDO2118_RS03835	L131805	core	o-succinylbenzoate synthase	None	K
llmg_2342	LLNZ_12105	NCDO2118_RS11310	L131806	core	Hypothetical protein	None	MR
llmg_0513	LLNZ_02650	NCDO2118_RS02660	L131937	core	UPF0398 protein	None	R
llmg_1447	LLNZ_07460	NCDO2118_RS05825	L132035	core	Hypothetical protein	None	S
llmg_2548	LLNZ_13165	NCDO2118_RS12480	L132038	core	Hypothetical protein	None	S
llmg_2070	LLNZ_10655	NCDO2118_RS10380	L132126	core	Hypothetical protein	None	U
llmg_1210	LLNZ_06230	NCDO2118_RS04780	L132251	core	Multidrug resistance protein	None	H
llmg_0352	LLNZ_01845	NCDO2118_RS01980	L132617	core	Oxidoreductase, aldo/keto reductase family	None	S
llmg_1446	LLNZ_07455	NCDO2118_RS05830	L132621	core	Hypothetical protein	None	R
llmg_2074	LLNZ_10670	NCDO2118_RS10390	L133490	core	Hypothetical protein	None	G
llmg_1625	LLNZ_08360	NCDO2118_RS04785	L133825	core	Hypothetical protein	None	F
llmg_0354	LLNZ_01855	NCDO2118_RS01990	L133932	core	Putative glyoxylase	None	S
llmg_1624	LLNZ_08355	NCDO2118_RS04790	L135727	core	Exopolysaccharide biosynthesis protein	None	V
llmg_2203	LLNZ_11370	NCDO2118_RS10725	L13744	core	Cadmium resistance protein	None	S
llmg_0358	LLNZ_01875	NCDO2118_RS02010	L137682	core	Penicillin-binding protein 2B	None	C
llmg_1622	LLNZ_08345	NCDO2118_RS04800	L138756	core	Teichoic acid ABC transporter permease protein	None	T
llmg_0211	LLNZ_01100	NCDO2118_RS01005	L1389	core	Rhamnosyltransferase RgpA	None	GM
llmg_2560	LLNZ_13235	NCDO2118_RS12515	L139168	core	DacA protein	None	M
llmg_1826	LLNZ_09395	NCDO2118_RS03870	L140288	core	Hypothetical protein	None	P
llmg_2561	LLNZ_13240	NCDO2118_RS12520	L140621	core	Glucose uptake protein glcU	None	S
llmg_2350	LLNZ_12150	NCDO2118_RS11355	L141091	core	Putative glycosyl transferase	None	T
llmg_0590	LLNZ_03030	NCDO2118_RS03215	L14190	core	Hypothetical protein	None	M
llmg_0733	LLNZ_03815	NCDO2118_RS09350	L142310	core	Cell envelope-related transcriptional attenuator	None	G
llmg_0152	LLNZ_00800	NCDO2118_RS00630	L142332	core	UPF0473 protein	None	K
llmg_2563	LLNZ_13250	NCDO2118_RS12530	L142355	core	Hypothetical protein	None	S
llmg_0153	LLNZ_00805	NCDO2118_RS00635	L142704	core	Hypothetical protein	None	S
llmg_0362	LLNZ_01895	NCDO2118_RS02030	L143312	core	Dipeptide-binding protein	None	S
llmg_0154	LLNZ_00810	NCDO2118_RS00640	L143350	core	Carbonyl reductase cbr	None	E
llmg_2145	LLNZ_11065	NCDO2118_RS10440	L143459	core	Hypothetical protein	None	IQR
llmg_1822	LLNZ_09380	NCDO2118_RS03885	L143879	core	Hypothetical protein	None	F
llmg_2146	LLNZ_11070	NCDO2118_RS10445	L144047	core	Hypothetical protein	None	S
llmg_0155	LLNZ_00815	NCDO2118_RS00645	L144155	core	Hypothetical protein	None	C
llmg_0156	LLNZ_00820	NCDO2118_RS00650	L144410	core	Oxidoreductase dltE	None	M
llmg_0731	LLNZ_03805	NCDO2118_RS09360	L144535	core	Hypothetical protein	None	M
llmg_2148	LLNZ_11080	NCDO2118_RS10455	L145420	core	Hypothetical protein	None	K
llmg_1155	LLNZ_05960	NCDO2118_RS07040	L145757	core	Glutaminase (Spx-like protein)	None	S
llmg_2149	LLNZ_11085	NCDO2118_RS10460	L145850	core	Putative Nudix hydrolase	None	P
llmg_2368	LLNZ_12235	NCDO2118_RS11445	L146261	core	Hypothetical protein	None	LR
llmg_0728	LLNZ_03790	NCDO2118_RS09375	L146623	core	PTS system, mannose-specific IIC component	None	S
llmg_0364	LLNZ_01910	NCDO2118_RS02040	L147009	core	Dipeptide transport system permease protein dppB	None	G
llmg_1425	LLNZ_07345	NCDO2118_RS05895	L147291	core	Transcriptional regulator, RpiR family	None	EP
llmg_0522	LLNZ_02700	NCDO2118_RS02795	L147423	core	Putative transcription regulator	None	K
llmg_0365	LLNZ_01915	NCDO2118_RS02045	L147936	core	Dipeptide transport system permease protein dppC	None	G
llmg_0523	LLNZ_02705	NCDO2118_RS02800	L148346	core	Glycerol-3-phosphate transporter	None	S
llmg_0162	LLNZ_00850	NCDO2118_RS00670	L148778	core	Hypothetical protein	None	S
llmg_1819	LLNZ_09365	NCDO2118_RS03905	L148897	core	Hypothetical protein	None	M
llmg_0725	LLNZ_03775	NCDO2118_RS09390	L148945	core	Hypothetical protein	None	S
llmg_0366	LLNZ_01920	NCDO2118_RS02050	L148976	core	Dipeptide transport ATP-binding protein dppD	None	P
llmg_1422	LLNZ_07335	NCDO2118_RS05905	L149164	core	Penicillin acylase	None	EP
llmg_0059	LLNZ_00290	NCDO2118_RS10735	L14951	core	Hypothetical protein	None	K
llmg_2155	LLNZ_11115	NCDO2118_RS10480	L149828	core	Guanylate kinase	None	S
llmg_1137	LLNZ_05870	NCDO2118_RS07105	L149891	core	Manganese transport system membrane protein	None	F
llmg_0527	LLNZ_02725	NCDO2118_RS02805	L150017	core	Hypothetical protein	None	P
llmg_0591	LLNZ_03035	NCDO2118_RS03220	L15012	core	tRNA dimethylallyltransferase	None	E
llmg_1606	LLNZ_08260	NCDO2118_RS04855	L150584	core	Glycerol-3-phosphate cytidylyltransferase	None	J
llmg_1420	LLNZ_07320	NCDO2118_RS07545	L151001	core	Hypothetical protein	None	P
llmg_1605	LLNZ_08255	NCDO2118_RS04860	L151067	core	Hypothetical protein	None	S
llmg_1000	LLNZ_05160	NCDO2118_RS07920	L151330	core	N5-carboxyaminoimidazole ribonucleotide synthase	None	S
llmg_1135	LLNZ_05860	NCDO2118_RS07115	L151700	core	Hypothetical protein	None	F
llmg_1419	LLNZ_07315	NCDO2118_RS05920	L152305	core	Putative transcription regulator	None	S
llmg_1603	LLNZ_08245	NCDO2118_RS04870	L152603	core	Teichoic acid biosynthesis protein B	None	L
llmg_0927	LLNZ_04770	NCDO2118_RS08240	L15267	core	Putative extracellular hydrolase	None	M
llmg_1894	LLNZ_09755	NCDO2118_RS09445	L152977	core	Phosphoglycerate mutase family protein	None	S
llmg_0529	LLNZ_02735	NCDO2118_RS02815	L153086	core	Putative transcription regulator	None	F
llmg_1602	LLNZ_08240	NCDO2118_RS04875	L153822	core	Putative transcription regulator	None	K
llmg_2385	LLNZ_12320	NCDO2118_RS11525	L153973	core	Large-conductance mechanosensitive channel	None	K
llmg_1417	LLNZ_07305	NCDO2118_RS05930	L154225	core	Hypothetical protein	None	M
llmg_2386	LLNZ_12325	NCDO2118_RS11530	L154438	core	Hypothetical protein	None	M
llmg_0533	LLNZ_02750	NCDO2118_RS02830	L155602	core	Putative (Di)nucleoside polyphosphate hydrolase	None	P
llmg_1600	LLNZ_08230	NCDO2118_RS04885	L156225	core	Hypothetical protein	None	S
llmg_1898	LLNZ_09775	NCDO2118_RS09465	L156251	core	Phosphate transport system permease protein PstA	None	S
llmg_0165	LLNZ_00865	NCDO2118_RS00760	L156674	core	Hypothetical protein	None	F
llmg_1413	LLNZ_07285	NCDO2118_RS05950	L157321	core	Hypothetical protein	None	K
llmg_1809	LLNZ_09315	NCDO2118_RS03960	L157908	core	Chromosome replication initiation / membrane attachment protein	None	R
llmg_2389	LLNZ_12340	NCDO2118_RS11545	L158071	core	Preprotein translocase SecE subunit	None	S
llmg_1130	LLNZ_05835	NCDO2118_RS07140	L158193	core	Hypothetical protein	None	P
llmg_2391	LLNZ_12350	NCDO2118_RS11555	L158566	core	Hypothetical protein	None	S
llmg_0167	LLNZ_00880	NCDO2118_RS00770	L158834	core	Hypothetical protein	None	F
llmg_1127	LLNZ_05825	NCDO2118_RS07145	L159364	core	Cell wall surface anchor family protein	None	M
llmg_1901	LLNZ_09790	NCDO2118_RS09480	L159386	core	Phosphate transport substrate binding protein pstF	None	S
llmg_1346	LLNZ_06960	NCDO2118_RS05960	L159396	core	Xanthine phosphoribosyltransferase	None	P
llmg_0168	LLNZ_00885	NCDO2118_RS00775	L159400	core	Deoxyribonuclease	None	F
llmg_1345	LLNZ_06955	NCDO2118_RS05965	L160092	core	Xanthine/uracil permease	None	C
llmg_0375	LLNZ_01960	NCDO2118_RS02090	L160176	core	Amino acid permease	None	F
llmg_1806	LLNZ_09300	NCDO2118_RS03975	L160697	core	Hypothetical protein	None	J
llmg_0011	LLNZ_00055	NCDO2118_RS00060	L16101	core	Hypothetical protein	None	S
llmg_1905	LLNZ_09810	NCDO2118_RS09490	L161059	core	Putative esterase	None	F
llmg_0992	LLNZ_05110	NCDO2118_RS07960	L161121	core	Putative nucleoside-diphosphate-sugar epimerases	None	E
llmg_1594	LLNZ_08200	NCDO2118_RS05220	L161266	core	Gamma-glutamyl-diamino acid-endopeptidase	None	I
llmg_0376	LLNZ_01965	NCDO2118_RS02095	L161441	core	Amino acid permease	None	M
llmg_1344	LLNZ_06950	NCDO2118_RS05970	L161455	core	Hypothetical protein	None	E
llmg_1126	LLNZ_05820	NCDO2118_RS07150	L161888	core	Hypothetical protein	None	S
llmg_1343	LLNZ_06945	NCDO2118_RS05975	L161988	core	tRNA (cytidine(34)-2'-O)-methyltransferase	None	S
llmg_2434	LLNZ_12580	NCDO2118_RS11795	L16221	core	Hypothetical protein	None	E
llmg_0991	LLNZ_05105	NCDO2118_RS07965	L162229	core	Transcriptional regulator, AcrR family	None	S
llmg_1804	LLNZ_09290	NCDO2118_RS03985	L162840	core	Transcriptional regulator, rgg family	None	E
llmg_1342	LLNZ_06940	NCDO2118_RS05980	L162872	core	Dihydrofolate reductase	None	U
llmg_1591	LLNZ_08190	NCDO2118_RS05230	L163056	core	Amino-acid ABC transporter permease protein	None	R
llmg_0378	LLNZ_01975	NCDO2118_RS02105	L163307	core	UPF0298 protein	None	E
llmg_2394	LLNZ_12365	NCDO2118_RS11570	L163677	core	Hypothetical protein	None	O
llmg_0379	LLNZ_01980	NCDO2118_RS02110	L163845	core	Hypothetical protein	None	S
llmg_1803	LLNZ_09285	NCDO2118_RS03990	L164132	core	Hypothetical protein	None	P
llmg_0988	LLNZ_05085	NCDO2118_RS07975	L164626	core	Phosphoribosylglycinamide formyltransferase	None	O

llmg_1908	LLNZ_09825	NCDO2118_RS09505	L165684	core	Putative methyltransferase	None	H
llmg_2398	LLNZ_12395	NCDO2118_RS11585	L165709	core	Zinc ABC transporter permease protein	None	S
llmg_1749	LLNZ_09035	NCDO2118_RS04235	L16606	core	Hypothetical protein	None	P
llmg_0542	LLNZ_02790	NCDO2118_RS02885	L166479	core	Pantothenic acid transporter	None	O
llmg_2399	LLNZ_12400	NCDO2118_RS11590	L166512	core	Zinc ABC transporter ATP binding protein	None	S
llmg_2435	LLNZ_12585	NCDO2118_RS11800	L16705	core	Hypothetical protein	None	H
llmg_0384	LLNZ_02005	NCDO2118_RS02135	L167555	core	Pseudouridine synthase	None	P
llmg_1802	LLNZ_09280	NCDO2118_RS03995	L167770	core	Hypothetical protein	None	R
llmg_1121	LLNZ_05795	NCDO2118_RS07175	L167877	core	Purine/cytidine ABC transporter permease protein	None	S
llmg_1335	LLNZ_06905	NCDO2118_RS06015	L168057	core	Probable DHNTP pyrophosphohydrolase	None	R
llmg_pseudo	LLNZ_09835	NCDO2118_RS09515	L168238	core	Competence protein CoiA	None	O
llmg_1956	LLNZ_10080	NCDO2118_RS09745	L16848	core	Hypothetical protein	None	S
llmg_0547	LLNZ_02810	NCDO2118_RS02905	L168650	core	Cation-transporting ATPase, E1-E2 family	None	O
llmg_2402	LLNZ_12415	NCDO2118_RS11605	L168783	core	Hypothetical protein	None	P
llmg_2403	LLNZ_12420	NCDO2118_RS11610	L169106	core	Hypothetical protein	None	S
llmg_1911	LLNZ_09840	NCDO2118_RS09520	L169301	core	Hypothetical protein	None	R
llmg_0177	LLNZ_00930	NCDO2118_RS00825	L169390	core	Amidase	None	S
llmg_2404	LLNZ_12425	NCDO2118_RS11615	L169515	core	Hypothetical protein	None	R
llmg_1801	LLNZ_09275	NCDO2118_RS04000	L169709	core	Sortase SrtC	None	NU
llmg_2405	LLNZ_12430	NCDO2118_RS11620	L169783	core	Putative competence protein ComGD	None	M
llmg_2168	LLNZ_11195	NCDO2118_RS10590	L169795	core	Hypothetical protein	None	NU
llmg_1584	LLNZ_08155	NCDO2118_RS05265	L169897	core	Endonuclease/exonuclease/phosphatase family protein	None	S
llmg_1912	LLNZ_09845	NCDO2118_RS09525	L170548	core	Hypothetical protein	None	F
llmg_0978	LLNZ_05025	NCDO2118_RS07995	L170617	core	Hypothetical protein	None	S
llmg_0178	LLNZ_00935	NCDO2118_RS00830	L170983	core	Damage-inducible protein DinF	None	G
llmg_1800	LLNZ_09270	NCDO2118_RS04005	L171000	core	Hypothetical protein	None	V
llmg_1583	LLNZ_08150	NCDO2118_RS05270	L171002	core	Putative prenyltransferase	None	S
llmg_0387	LLNZ_02020	NCDO2118_RS02150	L171116	core	Hypothetical protein	None	H
llmg_0977	LLNZ_05020	NCDO2118_RS08000	L171350	core	Amidophosphoribosyltransferase (ATase)	None	S
llmg_0434	LLNZ_02260	NCDO2118_RS02350	L17153	core	SanA protein homolog	None	F
llmg_1799	LLNZ_09265	NCDO2118_RS04010	L171802	core	LrgB family protein	None	F
llmg_2324	LLNZ_12005	NCDO2118_RS10755	L17182	core	Hypothetical protein	None	M
llmg_0388	LLNZ_02025	NCDO2118_RS02155	L171943	core	FAD:protein FMN transferase	None	S
llmg_1798	LLNZ_09260	NCDO2118_RS04015	L172519	core	LrgA family protein	None	K
llmg_0551	LLNZ_02830	NCDO2118_RS02920	L172704	core	Hypothetical protein	None	S
llmg_0181	LLNZ_00945	NCDO2118_RS00840	L172782	core	5-formyltetrahydrofolate cyclo-ligase	None	S
llmg_1330	LLNZ_06880	NCDO2118_RS06040	L173230	core	Niacin transporter NiaX	None	J
llmg_0182	LLNZ_00950	NCDO2118_RS00845	L173329	core	Rhomboid-related protein 1	None	S
llmg_0742	LLNZ_03865	NCDO2118_RS09220	L1734467	core	Maltose O-acetyltransferase	None	S
llmg_1303	LLNZ_06740	NCDO2118_RS02925	L173469	core	Hypothetical protein	None	R
llmg_1580	LLNZ_08135	NCDO2118_RS05285	L173848	core	Hypothetical protein	None	K
llmg_0976	LLNZ_05015	NCDO2118_RS08010	L173921	core	Phosphoribosylformylglycinamidine synthase subunit PurL	None	M
llmg_1302	LLNZ_06735	NCDO2118_RS02930	L174076	core	Hypothetical protein	None	F
llmg_1579	LLNZ_08130	NCDO2118_RS05290	L174407	core	Phosphoglycerate mutase	None	R
llmg_1301	LLNZ_06730	NCDO2118_RS02935	L174523	core	Hypothetical protein	None	G
llmg_1794	LLNZ_09245	NCDO2118_RS04030	L175136	core	Hypothetical protein	None	P
llmg_1578	LLNZ_08125	NCDO2118_RS05295	L175147	core	DacB protein	None	S
llmg_1577	LLNZ_08120	NCDO2118_RS05300	L175880	core	Hypothetical protein	None	Q
llmg_0730	LLNZ_03800	NCDO2118_RS09365	L176167	core	Ferredoxin	None	S
llmg_0975	LLNZ_05010	NCDO2118_RS08015	L176360	core	Phosphoribosylformylglycinamidine synthase subunit PurQ	None	S
llmg_2177	LLNZ_11245	NCDO2118_RS10620	L176399	core	Na+/H+ antiporter	None	F
llmg_0925	LLNZ_04760	NCDO2118_RS08360	L17640	core	Putative transcription regulator	None	P
llmg_1917	LLNZ_09870	NCDO2118_RS09550	L176579	core	Hypothetical protein	None	O
llmg_1327	LLNZ_06865	NCDO2118_RS06055	L176724	core	Spermidine/putrescine ABC transport system permease protein potB	None	S
llmg_1918	LLNZ_09875	NCDO2118_RS09555	L177346	core	Hypothetical protein	None	F
llmg_0973	LLNZ_05000	NCDO2118_RS08025	L177350	core	Phosphoribosylaminoimidazole-succinocarboxamide synthase	None	S
llmg_1326	LLNZ_06860	NCDO2118_RS06060	L177493	core	Spermidine/putrescine ABC transport permease protein potC	None	F
llmg_0187	LLNZ_00975	NCDO2118_RS00865	L177520	core	Cellobiose-specific PTS system IIC component	None	E
llmg_2410	LLNZ_12455	NCDO2118_RS11655	L177685	core	Hypothetical protein	None	G
llmg_0578	LLNZ_02970	NCDO2118_RS03090	L1778	core	tRNA-processing ribonuclease BN	None	R
llmg_1748	LLNZ_09030	NCDO2118_RS04240	L17781	core	Hypothetical protein	None	S
llmg_1919	LLNZ_09880	NCDO2118_RS09560	L178172	core	Hypothetical protein	None	S
llmg_0391	LLNZ_02040	NCDO2118_RS02170	L178384	core	Hypothetical protein	None	E
llmg_1112	LLNZ_05750	NCDO2118_RS07225	L178600	core	Hypothetical protein	None	S
llmg_0971	LLNZ_04990	NCDO2118_RS08035	L178621	core	Hypothetical protein	None	S
llmg_0435	LLNZ_02265	NCDO2118_RS02355	L17893	core	HTH-type transcriptional regulator	None	S
llmg_1111	LLNZ_05745	NCDO2118_RS07230	L179010	core	Heptaprenyl diphosphate synthase component I	None	R
llmg_1920	LLNZ_09885	NCDO2118_RS09565	L179243	core	Hypothetical protein	None	S
llmg_0969	LLNZ_04980	NCDO2118_RS08045	L179277	core	Hypothetical protein	None	S
llmg_0190	LLNZ_00990	NCDO2118_RS00880	L179659	core	Beta-glucosidase A	None	R
llmg_0968	LLNZ_04975	NCDO2118_RS08050	L179660	core	Oxidoreductase	None	G
llmg_0924	LLNZ_04755	NCDO2118_RS08365	L18033	core	Hypothetical protein	None	S
llmg_0967	LLNZ_04970	NCDO2118_RS08055	L180636	core	Putative permease protein	None	J
llmg_1108	LLNZ_05730	NCDO2118_RS07245	L181439	core	Hypothetical protein	None	S
llmg_2184	LLNZ_11275	NCDO2118_RS10645	L181807	core	Hypothetical protein	None	F
llmg_0394	LLNZ_02055	NCDO2118_RS02185	L181867	core	Putative permease protein	None	F
llmg_0966	LLNZ_04965	NCDO2118_RS08060	L182006	core	Transcriptional regulator, MarR family	None	P
llmg_1571	LLNZ_08090	NCDO2118_RS05325	L182020	core	Aminoacylase/N-acyl-L-amino acid amidohydrolase/hippurate hydrolase	None	G
llmg_1957	LLNZ_10085	NCDO2118_RS09750	L18206	core	ABC transporter, ATP-binding protein	None	G
llmg_2414	LLNZ_12480	NCDO2118_RS11675	L182799	core	Phosphatidate cytidylyltransferase	None	F
llmg_0193	LLNZ_01005	NCDO2118_RS00905	L183216	core	Hypothetical protein	None	H
llmg_2186	LLNZ_11285	NCDO2118_RS10655	L184033	core	Putative CMP-binding factor	None	I
llmg_1104	LLNZ_05710	NCDO2118_RS07265	L184818	core	Drug-export protein	None	U
llmg_0555	LLNZ_02850	NCDO2118_RS02990	L184871	core	Thymidine kinase	None	V
llmg_1568	LLNZ_08075	NCDO2118_RS05340	L185031	core	PTS system, fructose specific IIBC components	None	F
llmg_2417	LLNZ_12495	NCDO2118_RS11690	L185135	core	Hypothetical protein	None	G
llmg_2418	LLNZ_12500	NCDO2118_RS11695	L185851	core	Ribosomal RNA large subunit methyltransferase H	None	C
llmg_1747	LLNZ_09025	NCDO2118_RS04245	L18622	core	Amino acid permease	None	C
llmg_0398	LLNZ_02080	NCDO2118_RS02205	L186839	core	Hypothetical protein	None	S
llmg_1558	LLNZ_08005	NCDO2118_RS05345	L187016	core	Cardiolipin synthase	None	J
llmg_0558	LLNZ_02865	NCDO2118_RS03005	L187045	core	Hypothetical protein	None	I
llmg_0399	LLNZ_02085	NCDO2118_RS02210	L187315	core	Na+/H+ antiporter	None	S
llmg_2191	LLNZ_11310	NCDO2118_RS10670	L187329	core	Hypothetical protein	None	P
llmg_0197	LLNZ_01025	NCDO2118_RS00925	L187450	core	Prenyltransferase, UbiA family	None	S
llmg_0559	LLNZ_02870	NCDO2118_RS03010	L187716	core	NADPH-flavin oxidoreductase	None	H
llmg_2193	LLNZ_11320	NCDO2118_RS10680	L187815	core	Ribosome biogenesis GTPase RsgA	None	R
llmg_1102	LLNZ_05700	NCDO2118_RS07315	L187918	core	Hypothetical protein	None	J
llmg_0198	LLNZ_01030	NCDO2118_RS00930	L188392	core	Hypothetical protein	None	S
llmg_0560	LLNZ_02875	NCDO2118_RS03015	L188472	core	Release factor glutamine methyltransferase	None	S
llmg_2194	LLNZ_11325	NCDO2118_RS10685	L188798	core	Hypothetical protein	None	G
llmg_1930	LLNZ_09935	NCDO2118_RS09615	L188881	core	Membrane protein, putative	None	S
llmg_2010	LLNZ_10360	NCDO2118_RS10095	L1889726	core	UPF0213 protein	None	S
llmg_1101	LLNZ_05695	NCDO2118_RS07320	L188985	core	Hypothetical protein	None	L
llmg_0561	LLNZ_02880	NCDO2118_RS03020	L189315	core	Putative acetyltransferase	None	G
llmg_0400	LLNZ_02090	NCDO2118_RS02215	L189448	core	Hypothetical protein	None	S
llmg_0199	LLNZ_01040	NCDO2118_RS00940	L190009	core	Ferrous iron transport protein B	None	J
llmg_1555	LLNZ_07990	NCDO2118_RS05360	L190464	core	Sporulation transcription regulator WhiA	None	O
llmg_0402	LLNZ_02100	NCDO2118_RS02225	L191431	core	Penicillin-binding protein 1B	None	S

llmg_1554	LLNZ_07985	NCDO2118_RS05365	L191500	core	Transcriptional regulator, TetR family	None	M
llmg_0953	LLNZ_04895	NCDO2118_RS08115	L191765	core	Hypothetical protein	None	S
llmg_1553	LLNZ_07980	NCDO2118_RS05370	L192192	core	ABC transporter ATP binding protein	None	U
llmg_0200	LLNZ_01045	NCDO2118_RS00945	L192240	core	Ferrous iron transport protein A	None	U
llmg_0564	LLNZ_02895	NCDO2118_RS03035	L192354	core	Putative soluble lytic murein transglycosylase	None	P
llmg_1552	LLNZ_07975	NCDO2118_RS05375	L193030	core	ABC type transport system permease protein	None	O
llmg_1933	LLNZ_09960	NCDO2118_RS06075	L193090	core	Hypothetical protein	None	S
llmg_1771	LLNZ_09145	NCDO2118_RS04130	L193718	core	Putative rhodanese-related sulfurtransferase	None	O
llmg_2197	LLNZ_11340	NCDO2118_RS10700	L193734	core	PadC protein	None	P
llmg_1770	LLNZ_09140	NCDO2118_RS04135	L194050	core	NADH oxidase	None	J
llmg_pseudo	LLNZ_09970	NCDO2118_RS09640	L194765	core	Hypothetical protein	None	R
llmg_0405	LLNZ_02110	NCDO2118_RS02235	L195067	core	Hypothetical protein	None	S
llmg_0605	LLNZ_03105	NCDO2118_RS03255	L19516	core	Oxidoreductase	None	S
llmg_1551	LLNZ_07970	NCDO2118_RS05385	L195318	core	Formate dehydrogenase	None	S
llmg_1769	LLNZ_09135	NCDO2118_RS04140	L195720	core	Hypothetical protein	None	U
llmg_1937	LLNZ_09980	NCDO2118_RS09650	L196077	core	Hypothetical protein	None	P
llmg_2426	LLNZ_12540	NCDO2118_RS11735	L197055	core	Hypothetical protein	None	G
llmg_1548	LLNZ_07955	NCDO2118_RS05395	L197075	core	Putative phosphohydrolase	None	S
llmg_0439	LLNZ_02280	NCDO2118_RS02370	L19721	core	Transcriptional regulator, LacI family	None	S
llmg_1959	LLNZ_10100	NCDO2118_RS09760	L19745	core	Phosphinothricin N-acetyltransferase	None	K
llmg_1311	LLNZ_06780	NCDO2118_RS06140	L197809	core	Hypothetical protein	None	R
llmg_0945	LLNZ_04860	NCDO2118_RS08150	L198485	core	Glycerol dehydrogenase	None	M
llmg_0208	LLNZ_01085	NCDO2118_RS00990	L198940	core	Hypothetical protein	None	O
llmg_1941	LLNZ_09995	NCDO2118_RS09665	L199206	core	Hypothetical protein	None	S
llmg_2438	LLNZ_12605	NCDO2118_RS11815	L19959	core	HTH-type transcriptional regulator	None	M
llmg_0576	LLNZ_02960	NCDO2118_RS03080	L200264	core	Transcriptional regulator, TetR family	None	D
llmg_1960	LLNZ_10105	NCDO2118_RS09765	L20481	core	Hypothetical protein	None	S
llmg_2212	LLNZ_11410	NCDO2118_RS10780	L20683	core	Hypothetical protein	None	P
llmg_2213	LLNZ_11415	NCDO2118_RS10785	L21717	core	Hypothetical protein	None	S
llmg_1087	LLNZ_05625	NCDO2118_RS07340	L2183	core	N-acetylglucosaminidase	None	S
llmg_2443	LLNZ_12630	NCDO2118_RS11835	L21952	core	Phosphopantetheine adenylyltransferase	None	M
llmg_2444	LLNZ_12635	NCDO2118_RS11840	L22498	core	Hypothetical protein	None	V
llmg_2214	LLNZ_11420	NCDO2118_RS10790	L22691	core	Hypothetical protein	None	R
llmg_2445	LLNZ_12640	NCDO2118_RS11845	L23405	core	Multidrug resistance protein	None	R
llmg_1745	LLNZ_09015	NCDO2118_RS04255	L23500	core	Hypothetical protein	None	R
llmg_0442	LLNZ_02295	NCDO2118_RS02385	L23787	core	Hypothetical protein	None	S
llmg_1964	LLNZ_10125	NCDO2118_RS09785	L24031	core	Biotin transporter BioY	None	E
llmg_0232	LLNZ_01200	NCDO2118_RS01110	L24277	core	Hypothetical protein	None	Q
llmg_0922	LLNZ_04740	NCDO2118_RS08375	L24314	core	Hypothetical protein	None	LR
llmg_2446	LLNZ_12645	NCDO2118_RS11850	L24511	core	Multidrug resistance protein	None	S
llmg_1965	LLNZ_10130	NCDO2118_RS09790	L24742	core	Putative AMP-binding enzyme	None	G
llmg_0921	LLNZ_04735	NCDO2118_RS08380	L25107	core	Hypothetical protein	None	IQ
llmg_1524	LLNZ_07835	NCDO2118_RS05495	L25787	core	Pseudouridine synthase	None	X
llmg_0920	LLNZ_04730	NCDO2118_RS08385	L25903	core	Hypothetical protein	None	J
llmg_1760	LLNZ_09090	NCDO2118_RS04180	L2611	core	Queuosine precursor transporter	None	R
llmg_0236	LLNZ_01220	NCDO2118_RS01125	L26400	core	Probable nicotinate-nucleotide adenylyltransferase	None	S
llmg_2447	LLNZ_12655	NCDO2118_RS11860	L26428	core	Hypothetical protein	None	H
llmg_1742	LLNZ_09000	NCDO2118_RS04270	L26721	core	Putative regulator	None	I
llmg_1523	LLNZ_07830	NCDO2118_RS05500	L26878	core	Hypothetical protein	None	TQ
llmg_2218	LLNZ_11440	NCDO2118_RS10810	L27433	core	Predicted transcriptional regulator	None	R
llmg_1522	LLNZ_07825	NCDO2118_RS05505	L27464	core	Hypothetical protein	None	S
llmg_0579	LLNZ_02975	NCDO2118_RS03095	L2829	core	Hypothetical protein	None	J
llmg_0917	LLNZ_04710	NCDO2118_RS08400	L28525	core	Hypothetical protein	None	S
llmg_0240	LLNZ_01240	NCDO2118_RS01145	L28696	core	Hypothetical protein	None	P
llmg_1521	LLNZ_07820	NCDO2118_RS05510	L29089	core	Glycosyl transferase	None	QR
llmg_0241	LLNZ_01245	NCDO2118_RS01150	L29477	core	UPF0348 protein	None	C
llmg_2224	LLNZ_11465	NCDO2118_RS10835	L29672	core	CDP-diacylglycerol--glycerol-3-phosphate 3-phosphatidyltransferase	None	E
llmg_1740	LLNZ_08990	NCDO2118_RS04280	L30185	core	HTH-type transcriptional regulator	None	S
llmg_1519	LLNZ_07810	NCDO2118_RS05520	L30853	core	Hypothetical protein	None	S
llmg_2090	LLNZ_10750	NCDO2118_RS08540	L30909	core	Phage tail component	None	K
llmg_1970	LLNZ_10170	NCDO2118_RS09825	L31137	core	Hypothetical protein	None	X
llmg_2454	LLNZ_12690	NCDO2118_RS11885	L31269	core	Hypothetical protein	None	C
llmg_2226	LLNZ_11475	NCDO2118_RS10845	L31393	core	Peptidase, M16 family	None	S
llmg_1971	LLNZ_10175	NCDO2118_RS09830	L31711	core	Hypothetical protein	None	X
llmg_2092	LLNZ_10760	NCDO2118_RS05135	L32097	core	Hypothetical protein	None	M
llmg_1972	LLNZ_10180	NCDO2118_RS09835	L32195	core	Cysteine desulfurase	None	X
llmg_2093	LLNZ_10765	NCDO2118_RS05130	L32489	core	Hypothetical protein	None	E
llmg_1517	LLNZ_07800	NCDO2118_RS05530	L32731	core	Putative phosphatidic acid phosphatase	None	C
llmg_0941	LLNZ_04840	NCDO2118_RS08170	L3279	core	Acetyltransferase, GNAT family	None	S
llmg_1738	LLNZ_08975	NCDO2118_RS04290	L32812	core	Beta-glucosidase	None	I
llmg_0024	LLNZ_00115	NCDO2118_RS00165	L32907	core	PTS system, mannitol-specific IIA component	None	G
llmg_2094	LLNZ_10770	NCDO2118_RS08560	L32997	core	Hypothetical protein	None	G
llmg_2095	LLNZ_10775	NCDO2118_RS08565	L33322	core	Hypothetical protein	None	S
llmg_0025	LLNZ_00120	NCDO2118_RS00170	L33416	core	Mannitol-1-phosphate 5-dehydrogenase	None	S
llmg_1293	LLNZ_06680	NCDO2118_RS06355	L33782	core	Hypothetical protein	None	X
llmg_0213	LLNZ_01110	NCDO2118_RS01015	L3425	core	Transport permease protein	None	S
llmg_0248	LLNZ_01285	NCDO2118_RS01180	L34460	core	Transcriptional regulator, TetR family	None	G
llmg_2097	LLNZ_10785	NCDO2118_RS08575	L34899	core	ATP-dependent Clp protease proteolytic subunit	None	U
llmg_1736	LLNZ_08965	NCDO2118_RS04300	L35466	core	Hypothetical protein	None	G
llmg_2098	LLNZ_10790	NCDO2118_RS05105	L35649	core	Putative phage portal protein	None	S
llmg_1976	LLNZ_10205	NCDO2118_RS09850	L35751	core	Polysaccharide biosynthesis protein	None	R
llmg_2460	LLNZ_12715	NCDO2118_RS11905	L35832	core	Hypothetical protein	None	S
llmg_2231	LLNZ_11500	NCDO2118_RS10870	L35965	core	Transcriptional regulator, MerR family	None	M
llmg_2461	LLNZ_12720	NCDO2118_RS11910	L36177	core	Hypothetical protein	None	G
llmg_2232	LLNZ_11505	NCDO2118_RS10875	L36511	core	Hypothetical protein	None	S
llmg_0250	LLNZ_01295	NCDO2118_RS01190	L36748	core	Transcriptional regulator, MerR family	None	S
llmg_2099	LLNZ_10795	NCDO2118_RS08585	L36824	core	Putative head-tail joining protein	None	R
llmg_2100	LLNZ_10800	NCDO2118_RS05095	L37002	core	Putative terminase large subunit	None	S
llmg_1288	LLNZ_06655	NCDO2118_RS06380	L37351	core	Histidinol-phosphatase	None	G
llmg_2534	LLNZ_13090	NCDO2118_RS12135	L37667	core	Hypothetical protein	None	G
llmg_0454	LLNZ_02350	NCDO2118_RS02440	L37906	core	Beta-glucosides-specific IIBC component	None	R
llmg_2102	LLNZ_10820	NCDO2118_RS08595	L38962	core	Phage terminase small subunit	None	F
llmg_1980	LLNZ_10225	NCDO2118_RS09870	L39484	core	Hypothetical protein	None	S
llmg_2103	LLNZ_10825	NCDO2118_RS05085	L39564	core	Hypothetical protein	None	R
llmg_2532	LLNZ_13075	NCDO2118_RS12125	L40104	core	Hypothetical protein	None	C
llmg_0033	LLNZ_00160	NCDO2118_RS12115	L40611	core	Hypothetical protein	None	X
llmg_0034	LLNZ_00165	NCDO2118_RS12110	L40862	core	Hypothetical protein	None	X
llmg_0905	LLNZ_04650	NCDO2118_RS08465	L40973	core	Hypothetical protein	None	X
llmg_2260	LLNZ_11660	NCDO2118_RS12100	L41670	core	Hypothetical phage protein predicted by Glimmer/Critica	None	C
llmg_0037	LLNZ_00180	NCDO2118_RS12095	L42195	core	Hypothetical protein	None	R
llmg_0903	LLNZ_04640	NCDO2118_RS08470	L42411	core	Serine/threonine phosphatase	None	X
llmg_0457	LLNZ_02365	NCDO2118_RS02455	L42784	core	Lipase/esterase (Putative)	None	S
llmg_0816	LLNZ_04210	NCDO2118_RS05015	L42912	core	Deoxyuridine 5'-triphosphate nucleotidohydrolase	None	R
llmg_2466	LLNZ_12745	NCDO2118_RS11930	L43222	core	Regulatory protein RecX	None	I
llmg_0458	LLNZ_02370	NCDO2118_RS02460	L43651	core	Glucosyltransferase-I	None	E
llmg_0255	LLNZ_01320	NCDO2118_RS01240	L44063	core	Dihydroxyacetone kinase DhaK	None	F
llmg_1732	LLNZ_08945	NCDO2118_RS04320	L44083	core	L-serine dehydratase alpha subunit	None	EF
llmg_0042	LLNZ_00205	NCDO2118_RS12075	L44085	core	Hypothetical protein	None	G

llmg_1281	LLNZ_06610	NCDO2118_RS06415	L44550	core	ABC transporter ATP binding protein	None	R
llmg_0460	LLNZ_02380	NCDO2118_RS02475	L45003	core	Hypothetical acetyltransferase	None	F
llmg_pseudo	LLNZ_01325	NCDO2118_RS01245	L45062	core	HTH-type dhaKLM operon transcriptional activator DhaS	None	S
llmg_0581	LLNZ_02980	NCDO2118_RS03110	L4536	core	Hypothetical protein	None	K
llmg_0891	LLNZ_04585	NCDO2118_RS08805	L46118	core	Uracil permease	None	P
llmg_0257	LLNZ_01340	NCDO2118_RS01255	L46694	core	Dihydroxyacetone kinase DhaM	None	P
llmg_0258	LLNZ_01345	NCDO2118_RS01260	L47257	core	Hypothetical protein	None	R
llmg_2238	LLNZ_11535	NCDO2118_RS10915	L47971	core	Hypothetical protein	None	I
llmg_0889	LLNZ_04575	NCDO2118_RS08815	L48287	core	Hypothetical protein	None	T
llmg_1727	LLNZ_08920	NCDO2118_RS04340	L48341	core	ABC transporter permease protein	None	T
llmg_1984	LLNZ_10245	NCDO2118_RS09890	L48903	core	Transcriptional regulator	None	S
llmg_0887	LLNZ_04565	NCDO2118_RS08825	L49325	core	Cation transport protein	None	G
llmg_0061	LLNZ_00305	NCDO2118_RS00180	L50174	core	Putative acetyltransferase	None	R
llmg_0062	LLNZ_00310	NCDO2118_RS00185	L50682	core	Hypothetical protein	None	S
llmg_2242	LLNZ_11560	NCDO2118_RS10930	L51032	core	Hypothetical protein	None	S
llmg_0262	LLNZ_01375	NCDO2118_RS01285	L51437	core	Transport permease protein	None	R
llmg_0624	LLNZ_03225	NCDO2118_RS03305	L52030	core	ABC transporter ATP binding protein	None	G
llmg_0884	LLNZ_04550	NCDO2118_RS08840	L52568	core	Hypothetical protein	None	R
llmg_1274	LLNZ_06575	NCDO2118_RS06450	L52644	core	Putative regulator AldR	None	S
llmg_0625	LLNZ_03230	NCDO2118_RS03310	L52704	core	ABC transporter ATP binding protein	None	Q
llmg_0803	LLNZ_04150	NCDO2118_RS08720	L52711	core	Putative transcription regulator	None	U
llmg_0626	LLNZ_03235	NCDO2118_RS03315	L53789	core	Putative transcription regulator	None	G
llmg_2006	LLNZ_10340	NCDO2118_RS08775	L53979	core	Hypothetical protein	None	M
llmg_0264	LLNZ_01385	NCDO2118_RS01295	L54021	core	Fructose-1,6-bisphosphatase class 3	None	X
llmg_0882	LLNZ_04540	NCDO2118_RS08850	L54406	core	Hypothetical protein	None	S
llmg_0881	LLNZ_04535	NCDO2118_RS08855	L54719	core	Branched-chain amino acid transport protein azlC	None	C
llmg_1720	LLNZ_08870	NCDO2118_RS04360	L54803	core	Uridine phosphorylase	None	E
llmg_1540	LLNZ_07915	NCDO2118_RS05435	L5517	core	Glycerol-3-phosphate acyltransferase	None	K
llmg_2245	LLNZ_11580	NCDO2118_RS10955	L55194	core	Hypothetical protein	None	S
llmg_2471	LLNZ_12770	NCDO2118_RS11975	L55836	core	Hypothetical protein	None	C
llmg_0879	LLNZ_04525	NCDO2118_RS08865	L56232	core	Spermidine acetyltransferase	None	R
llmg_2472	LLNZ_12775	NCDO2118_RS11980	L56236	core	Hypothetical protein	None	J
llmg_0628	LLNZ_03245	NCDO2118_RS03325	L56275	core	Divalent cation transport-related protein	None	S
llmg_2270	LLNZ_11740	NCDO2118_RS10970	L57113	core	ABC-type transporter ATP-binding protein	None	E
llmg_1069	LLNZ_05525	NCDO2118_RS07465	L57401	core	UPF0122 protein	None	U
llmg_0583	LLNZ_02990	NCDO2118_RS03120	L5776	core	Prolipoprotein diacylglyceryl transferase	None	R
llmg_1270	LLNZ_06555	NCDO2118_RS06470	L57903	core	Tyrosine recombinase XerS	None	M
llmg_2476	LLNZ_12795	NCDO2118_RS12000	L58643	core	Undecaprenyl-diphosphatase	None	S
llmg_0268	LLNZ_01405	NCDO2118_RS01330	L59135	core	ABC transporter ATP binding protein	None	S
llmg_1269	LLNZ_06550	NCDO2118_RS06475	L59266	core	Hypothetical protein	None	U
llmg_2477	LLNZ_12800	NCDO2118_RS12005	L59950	core	Lysine specific permease	None	H
llmg_0631	LLNZ_03260	NCDO2118_RS03340	L60571	core	Multidrug resistance efflux pump	None	E
llmg_2261	LLNZ_11665	NCDO2118_RS10050	L6094	core	Hypothetical phage protein predicted by Glimmer/Critica	None	E
llmg_0937	LLNZ_04820	NCDO2118_RS08190	L6128	core	tRNA (guanine-N(1)-)-methyltransferase	None	S
llmg_1705	LLNZ_08790	NCDO2118_RS04385	L61355	core	Holo-[acyl-carrier-protein] synthase	None	J
llmg_1995	LLNZ_10295	NCDO2118_RS09940	L61866	core	Hypothetical protein	None	K
llmg_1085	LLNZ_05615	NCDO2118_RS07350	L6208	core	Hypothetical protein	None	S
llmg_0633	LLNZ_03270	NCDO2118_RS03350	L62179	core	Cell division protein ftsW1	None	S
llmg_0821	LLNZ_04235	NCDO2118_RS08610	L62774	core	Hypothetical protein	None	P
llmg_0270	LLNZ_01415	NCDO2118_RS01340	L62955	core	Hypothetical protein	None	F
llmg_1061	LLNZ_05485	NCDO2118_RS07505	L63985	core	Hypothetical protein	None	S
llmg_1237	LLNZ_06370	NCDO2118_RS01450	L64332	core	N5-carboxyethyl-ornithine synthase	None	F
llmg_1700	LLNZ_08770	NCDO2118_RS04405	L64737	core	Choline ABC transporter ATP binding protein	None	S
llmg_1699	LLNZ_08765	NCDO2118_RS04410	L65675	core	Choline ABC transporter permease and substrate binding protein	None	C
llmg_1259	LLNZ_06485	NCDO2118_RS01435	L66209	core	Hypothetical protein	None	E
llmg_2000	LLNZ_10315	NCDO2118_RS09960	L66281	core	Putative permease	None	Q
llmg_1257	LLNZ_06475	NCDO2118_RS01425	L67002	core	Hypothetical protein	None	S
llmg_0635	LLNZ_03280	NCDO2118_RS03360	L67186	core	Citrate synthase	None	E
llmg_1057	LLNZ_05465	NCDO2118_RS07525	L67275	core	PsiE protein homolog	None	R
llmg_0870	LLNZ_04475	NCDO2118_RS08905	L67370	core	Transporter	None	S
llmg_0584	LLNZ_02995	NCDO2118_RS03125	L6768	core	Hypothetical protein	None	T
llmg_2281	LLNZ_11795	NCDO2118_RS11025	L67708	core	Peptide methionine sulfoxide reductase	None	S
llmg_1056	LLNZ_05460	NCDO2118_RS07530	L67760	core	Cation transporter	None	O
llmg_0278	LLNZ_01455	NCDO2118_RS01370	L67872	core	Hypothetical protein	None	P
llmg_2007	LLNZ_10345	NCDO2118_RS10075	L68066	core	Hypothetical protein	None	S
llmg_0279	LLNZ_01460	NCDO2118_RS01375	L68363	core	Hypothetical protein	None	L
llmg_0636	LLNZ_03285	NCDO2118_RS03365	L68478	core	Aconitate hydratase	None	R
llmg_0869	LLNZ_04470	NCDO2118_RS08910	L68605	core	Hypothetical protein	None	C
llmg_1055	LLNZ_05455	NCDO2118_RS07535	L69000	core	Transcriptional regulator	None	J
llmg_1084	LLNZ_05610	NCDO2118_RS07355	L6927	core	Hypothetical protein	None	G
llmg_2283	LLNZ_11805	NCDO2118_RS11035	L69304	core	Hypothetical protein	None	S
llmg_0081	LLNZ_00400	NCDO2118_RS00260	L69353	core	Amino-acid permease	None	S
llmg_1054	LLNZ_05450	NCDO2118_RS07540	L69383	core	Hypothetical protein	None	E
llmg_2427	LLNZ_12545	NCDO2118_RS11765	L6973	core	Competence regulator ComX	None	S
llmg_1694	LLNZ_08740	NCDO2118_RS04455	L70979	core	Copper-potassium transporting ATPase B	None	F
llmg_0637	LLNZ_03290	NCDO2118_RS03370	L71075	core	Isocitrate dehydrogenase	None	P
llmg_2286	LLNZ_11820	NCDO2118_RS11050	L71425	core	Hypothetical protein	None	R
llmg_1051	LLNZ_05435	NCDO2118_RS07550	L71486	core	Transcriptional regulator	None	S
llmg_1050	LLNZ_05430	NCDO2118_RS07555	L72115	core	Hypothetical protein	None	K
llmg_0282	LLNZ_01475	NCDO2118_RS01390	L72609	core	Anaerobic ribonucleoside-triphosphate reductase-activating protein	None	E
llmg_1513	LLNZ_07780	NCDO2118_RS05550	L72684	core	Hypothetical protein	None	O
llmg_0283	LLNZ_01480	NCDO2118_RS01395	L73386	core	Hypothetical protein	None	U
llmg_2011	LLNZ_10365	NCDO2118_RS10100	L73435	core	Amino acid permease	None	S
llmg_0861	LLNZ_04425	NCDO2118_RS08280	L74167	core	Hypothetical protein	None	K
llmg_0642	LLNZ_03315	NCDO2118_RS03390	L74660	core	Hypothetical protein	None	E
llmg_2390	LLNZ_12345	NCDO2118_RS11550	L74935	core	50S ribosomal protein L33 3	None	S
llmg_0643	LLNZ_03320	NCDO2118_RS03395	L76107	core	Cation-transporting ATPase, E1-E2 family	None	R
llmg_2013	LLNZ_10375	NCDO2118_RS10110	L76216	core	Thiamin pyrophosphokinase	None	S
llmg_2015	LLNZ_10385	NCDO2118_RS10120	L77220	core	Hypothetical protein	None	D
llmg_0289	LLNZ_01520	NCDO2118_RS01675	L77627	core	Energy-coupling factor transporter transmembrane protein EcfT	None	R
llmg_0055	LLNZ_00275	NCDO2118_RS10065	L7798	core	Phage integrase	None	P
llmg_2294	LLNZ_11865	NCDO2118_RS11080	L78133	core	Hypothetical protein	None	X
llmg_2295	LLNZ_11870	NCDO2118_RS11085	L78607	core	Hypothetical protein	None	S
llmg_2492	LLNZ_12875	NCDO2118_RS12230	L79329	core	Hypothetical protein	None	E
llmg_0095	LLNZ_00475	NCDO2118_RS00320	L79507	core	Putative esterase	None	S
llmg_0292	LLNZ_01535	NCDO2118_RS01690	L80177	core	N-acetyldiaminopimelate deacetylase	None	S
llmg_0096	LLNZ_00480	NCDO2118_RS00325	L80191	core	Putatve glyoxylase protein	None	R
llmg_0856	LLNZ_04400	NCDO2118_RS08975	L80679	core	Hypothetical protein	None	R
llmg_0097	LLNZ_00485	NCDO2118_RS00330	L81206	core	Flavoprotein oxygenase	None	F
llmg_2020	LLNZ_10410	NCDO2118_RS10140	L81319	core	Hypothetical protein	None	S
llmg_0849	LLNZ_04370	NCDO2118_RS07445	L81862	core	Hypothetical protein	None	I
llmg_0789	LLNZ_04090	NCDO2118_RS08980	L82310	core	Ribose ABC transporter substrate binding protein	None	K
llmg_2297	LLNZ_11875	NCDO2118_RS11105	L82956	core	Hypothetical protein	None	S
llmg_2496	LLNZ_12895	NCDO2118_RS12250	L83104	core	Hypothetical protein	None	S
llmg_0788	LLNZ_04085	NCDO2118_RS08985	L83296	core	Ribose transport system permease protein	None	S
llmg_2497	LLNZ_12900	NCDO2118_RS12255	L83481	core	UPF0721 transmembrane protein	None	G
llmg_1684	LLNZ_08680	NCDO2118_RS04510	L85237	core	Hypothetical protein	None	S
llmg_0786	LLNZ_04075	NCDO2118_RS08995	L85737	core	D-ribose pyranase	None	P

llmg_0464	LLNZ_02400	NCDO2118_RS02500	L85854	core	Thiamine precursor transporter	None	S
llmg_1683	LLNZ_08675	NCDO2118_RS04515	L85895	core	Hypothetical protein	None	S
llmg_0785	LLNZ_04070	NCDO2118_RS09000	L86157	core	Ribokinase	None	S
llmg_1703	LLNZ_08780	NCDO2118_RS04395	L862989	core	Hypothetical protein	None	R
llmg_1682	LLNZ_08670	NCDO2118_RS04520	L86448	core	Hypothetical protein	None	S
llmg_0651	LLNZ_03370	NCDO2118_RS03405	L86554	core	Putative acetyltransferase	None	S
llmg_0298	LLNZ_01565	NCDO2118_RS01720	L86677	core	SprT-like protein	None	S
llmg_1083	LLNZ_05605	NCDO2118_RS07365	L8702	core	Hypothetical protein	None	E
llmg_1496	LLNZ_07700	NCDO2118_RS05625	L87113	core	Putative phosphohydrolase	None	S
llmg_1538	LLNZ_07905	NCDO2118_RS05445	L8730	core	Putative isochorismatase	None	S
llmg_1884	LLNZ_09705	NCDO2118_RS03645	L88237	core	DNA topology modulation protein FlaR	None	F
llmg_1222	LLNZ_06295	NCDO2118_RS06690	L88729	core	D-alanine transfer protein DltD	None	D
llmg_1883	LLNZ_09700	NCDO2118_RS03650	L88730	core	Ribonuclease M5	None	M
llmg_1494	LLNZ_07690	NCDO2118_RS05635	L89001	core	ABC transporter ATP binding protein	None	L
llmg_1220	LLNZ_06285	NCDO2118_RS06700	L90287	core	Protein dltB	None	M
llmg_0700	LLNZ_03640	NCDO2118_RS10175	L90358	core	Oligopeptide transport system permease protein	None	M
llmg_1881	LLNZ_09690	NCDO2118_RS03660	L90422	core	Aminopeptidase P	None	EP
llmg_1045	LLNZ_05405	NCDO2118_RS07590	L90678	core	Similar to PTS system, beta-glucosides specific enzyme IIABC	None	S
llmg_1537	LLNZ_07900	NCDO2118_RS05450	L9094	core	DNA polymerase III, epsilon chain	None	Q
llmg_0699	LLNZ_03635	NCDO2118_RS10180	L91252	core	Peptide transport system permease protein oppB	None	S
llmg_1490	LLNZ_07670	NCDO2118_RS05655	L91569	core	Divalent metal cation transporter MntH	None	M
llmg_0307	LLNZ_01605	NCDO2118_RS01750	L92414	core	Ribosomal-protein-alanine N-acetyltransferase	None	S
llmg_1677	LLNZ_08640	NCDO2118_RS04545	L92573	core	Hypothetical protein	None	K
llmg_0479	LLNZ_02480	NCDO2118_RS02540	L92927	core	Cytokinin riboside 5'-monophosphate phosphoribohydrolase	None	T
llmg_1676	LLNZ_08635	NCDO2118_RS04550	L92987	core	ABC transporter permease protein	None	S
llmg_0308	LLNZ_01610	NCDO2118_RS01755	L93051	core	Ribosomal-protein-alanine acetyltransferase	None	U
llmg_0480	LLNZ_02485	NCDO2118_RS02545	L93481	core	Deoxyguanosine kinase	None	S
llmg_0309	LLNZ_01615	NCDO2118_RS01760	L93500	core	tRNA N6-adenosine threonylcarbamoyltransferase	None	F
llmg_0777	LLNZ_04035	NCDO2118_RS09040	L93855	core	Hypothetical protein	None	E
llmg_1488	LLNZ_07660	NCDO2118_RS05665	L93858	core	Putative hydrolase	None	S
llmg_pseudo	LLNZ_09655	NCDO2118_RS03680	L94405	core	4-alpha-glucanotransferase	None	S
llmg_1082	LLNZ_05600	NCDO2118_RS07370	L9467	core	Hypothetical protein	None	P
llmg_1675	LLNZ_08630	NCDO2118_RS04555	L94760	core	ABC transporter ATP-binding protein	None	S
llmg_1216	LLNZ_06265	NCDO2118_RS06725	L94809	core	Hydroxyethylthiazole kinase	None	R
llmg_1486	LLNZ_07650	NCDO2118_RS05675	L95012	core	DgkA protein	None	P
llmg_1485	LLNZ_07645	NCDO2118_RS05680	L95481	core	Hypothetical protein	None	J
llmg_1040	LLNZ_05365	NCDO2118_RS07610	L95697	core	Acetyltransferase, GNAT family	None	S
llmg_1874	LLNZ_09650	NCDO2118_RS03685	L95975	core	Glucose-1-phosphate adenylyltransferase	None	J
llmg_2033	LLNZ_10480	NCDO2118_RS10210	L96329	core	Hypothetical protein	None	P
llmg_0100	LLNZ_00500	NCDO2118_RS00345	L96595	core	Cation-transporting ATPase	None	S
llmg_1672	LLNZ_08610	NCDO2118_RS04565	L96605	core	Hypothetical protein	None	P
llmg_2034	LLNZ_10485	NCDO2118_RS10215	L96903	core	Hydrolase, haloacid dehalogenase-like family	None	R
llmg_1873	LLNZ_09645	NCDO2118_RS03690	L97131	core	Glucose-1-phosphate adenylyltransferase	None	S
llmg_0313	LLNZ_01635	NCDO2118_RS01785	L97415	core	Phosphonates import ATP-binding protein PhnC	None	R
llmg_0491	LLNZ_02540	NCDO2118_RS02555	L97827	core	Hemolysin like protein	None	O
llmg_1482	LLNZ_07630	NCDO2118_RS05695	L98095	core	Hypothetical protein	None	R
llmg_2508	LLNZ_12955	NCDO2118_RS12305	L98132	core	Cell shape determining protein mreD	None	S
llmg_0314	LLNZ_01640	NCDO2118_RS01790	L98204	core	Phosphonate transport system permease protein phnB	None	M
llmg_1872	LLNZ_09640	NCDO2118_RS03695	L98347	core	Glycogen synthase	None	P
llmg_1667	LLNZ_08580	NCDO2118_RS04580	L98542	core	Glycosyltransferase	None	G
llmg_1481	LLNZ_07625	NCDO2118_RS05700	L98876	core	Putative teicoplanin resistance protein	None	R
llmg_0315	LLNZ_01645	NCDO2118_RS01795	L99004	core	Phosphonate ABC transporter permease protein	None	S
llmg_0103	LLNZ_00515	NCDO2118_RS00355	L99272	core	Chromosome segregation helicase	None	P
llmg_1480	LLNZ_07620	NCDO2118_RS05705	L99421	core	Hypothetical protein	None	L
llmg_0493	LLNZ_02550	NCDO2118_RS02565	L99502	core	Putative esterase/lipase	None	S
llmg_0316	LLNZ_01650	NCDO2118_RS01800	L99798	core	2',3'-cyclic-nucleotide 2'-phosphodiesterase	None	G

COG groups are defined in the legend to Fig. 2B.
