# Supplementary material for: Comparative proteomic analysis of four biotechnological strains Lactococcus lactis through label‐free quantitative proteomics
Source: Microb Biotechnol. 2018 Oct 19;12(2):265–74. doi: 10.1111/1751-7915.13305 (PMC6389847; doi:10.1111/1751-7915.13305)
Supplement: Supplementary file 3 — Table S2. Total list of identified proteins on the protein abundance scale. [file MBT2-12-265-s003.pdf]

Supplementary File 5: Total list of identified proteins on the protein abundance scale.

Accession	Ave_Log(10)	rankByLog10	Tag	COG Function
NCDO2118_RS05460	4,67252266	1	Red	L
NCDO2118_RS11475	4,49863451	2	Red	J
NCDO2118_RS09495	4,390439133	3	Red	J
NCDO2118_RS06745	4,355746704	4	Red	J
NCDO2118_RS10290	4,335112586	5	Red	J
NCDO2118_RS02240	4,28371466	6	Orange	OC
NCDO2118_RS01995	4,255191174	7	Orange	G
NCDO2118_RS11995	4,252182123	8	Orange	J
NCDO2118_RS05315	4,244170386	9	Orange	O
NCDO2118_RS01235	4,241129363	10	Orange	G
NCDO2118_RS00460	4,236944292	11	Orange	G
NCDO2118_RS11775	4,213205438	12	Orange	J
NCDO2118_RS11515	4,206788774	13	Orange	J
NCDO2118_RS11420	4,200476581	14	Orange	J
NCDO2118_RS04165	4,199177613	15	Orange	J
NCDO2118_RS11435	4,184297805	16	Orange	J
NCDO2118_RS10530	4,161947737	17	Orange	G
NCDO2118_RS11780	4,156960956	18	Orange	J
NCDO2118_RS07985	4,151638613	19	Orange	O
NCDO2118_RS05280	4,150976583	20	Orange	S
NCDO2118_RS09875	4,148797022	21	Orange	K
NCDO2118_RS11440	4,147132126	22	Orange	J
NCDO2118_RS11375	4,13321315	23	Orange	J
NCDO2118_RS12500	4,129373927	24	Orange	J
NCDO2118_RS04355	4,12803792	25	Orange	J
NCDO2118_RS10615	4,122190909	26	Orange	F
NCDO2118_RS09695	4,12019179	27	Orange	C
NCDO2118_RS02580	4,1160699	28	Orange	L
NCDO2118_RS02690	4,092723757	29	Orange	O
NCDO2118_RS03070	4,078442136	30	Orange	C
NCDO2118_RS05900	4,075105097	31	Orange	G
NCDO2118_RS11425	4,071673017	32	Orange	J
NCDO2118_RS00835	4,066498761	33	Orange	K
NCDO2118_RS07185	4,063101787	34	Orange	G
NCDO2118_RS11510	4,059754546	35	Orange	J
NCDO2118_RS00005	4,041791765	36	Orange	L
NCDO2118_RS11170	4,039844945	37	Orange	E
NCDO2118_RS10330	4,03592445	38	Orange	D
NCDO2118_RS00455	4,024679774	39	Orange	G
NCDO2118_RS09065	4,022272267	40	Orange	D
NCDO2118_RS12505	4,005782741	41	Orange	J
NCDO2118_RS11985	4,003299913	42	Orange	J
NCDO2118_RS11500	4,001774006	43	Orange	J
NCDO2118_RS01350	3,997611964	44	dark blue	T
NCDO2118_RS11185	3,997478103	45	dark blue	J
NCDO2118_RS07190	3,986879964	46	dark blue	G
NCDO2118_RS11415	3,985134674	47	dark blue	J
NCDO2118_RS01140	3,957011356	48	dark blue	S
NCDO2118_RS04880	3,954332671	49	dark blue	G
NCDO2118_RS12385	3,952038714	50	dark blue	G
NCDO2118_RS00620	3,942180669	51	dark blue	S
NCDO2118_RS04080	3,937744242	52	dark blue	IQ
NCDO2118_RS03300	3,936460514	53	dark blue	G
NCDO2118_RS04615	3,93041169	54	dark blue	R
NCDO2118_RS11455	3,928457354	55	dark blue	J
NCDO2118_RS08915	3,928133543	56	dark blue	G
NCDO2118_RS11380	3,896226151	57	dark blue	K
NCDO2118_RS03980	3,89193744	58	dark blue	R
NCDO2118_RS01710	3,889816642	59	dark blue	J
NCDO2118_RS11460	3,887956297	60	dark blue	J
NCDO2118_RS10260	3,883279291	61	dark blue	J
NCDO2118_RS11405	3,878006186	62	dark blue	F
NCDO2118_RS10305	3,870043218	63	dark blue	D
NCDO2118_RS09710	3,866478024	64	dark blue	C
NCDO2118_RS11540	3,860071997	65	dark blue	K
NCDO2118_RS00470	3,858541067	66	dark blue	S
NCDO2118_RS11505	3,858247363	67	dark blue	J
NCDO2118_RS09705	3,852289736	68	dark blue	C
NCDO2118_RS00805	3,84476192	69	dark blue	J
NCDO2118_RS08870	3,842089375	70	dark blue	U
NCDO2118_RS05650	3,836714874	71	dark blue	J
NCDO2118_RS10150	3,835484419	72	dark blue	T

NCDO2118_RS11390	3,83408019	73 dark blue	J
NCDO2118_RS02000	3,82785228	74 dark blue	O
NCDO2118_RS11715	3,827333229	75 dark blue	S
NCDO2118_RS00230	3,826609711	76 dark blue	C
NCDO2118_RS02345	3,825457832	77 dark blue	L
NCDO2118_RS11465	3,807869783	78 dark blue	J
NCDO2118_RS04475	3,792141764	79 dark blue	L
NCDO2118_RS11060	3,790493867	80 dark blue	C
NCDO2118_RS09675	3,789804982	81 dark blue	S
NCDO2118_RS02365	3,785444667	82 dark blue	G
NCDO2118_RS02360	3,785390532	83 dark blue	G
NCDO2118_RS04275	3,784869401	84 dark blue	J
NCDO2118_RS10335	3,781575745	85 dark blue	D
NCDO2118_RS09105	3,781207856	86 dark blue	C
NCDO2118_RS00220	3,77819849	87 dark blue	C
NCDO2118_RS11790	3,775939593	88 dark blue	C
NCDO2118_RS06750	3,773371536	89 dark blue	J
NCDO2118_RS00035	3,773161377	90 dark blue	J
NCDO2118_RS09670	3,772612256	91 dark blue	ET
NCDO2118_RS11770	3,771558671	92 dark blue	D
NCDO2118_RS09880	3,767470179	93 dark blue	K
NCDO2118_RS10795	3,764602801	94 dark blue	J
NCDO2118_RS04040	3,763557635	95 dark blue	J
NCDO2118_RS07330	3,749002561	96 dark blue	EF
NCDO2118_RS02835	3,748886418	97 dark blue	L
NCDO2118_RS02790	3,748333443	98 dark blue	K
NCDO2118_RS09050	3,742995267	99 dark blue	K
NCDO2118_RS03330	3,738234809	100 dark blue	C
NCDO2118_RS02335	3,735992987	101 dark blue	P
NCDO2118_RS11490	3,735304854	102 dark blue	J
NCDO2118_RS01100	3,733145211	103 dark blue	D
NCDO2118_RS02280	3,73126719	104 dark blue	TK
NCDO2118_RS07765	3,72939183	105 dark blue	F
NCDO2118_RS10485	3,727817348	106 dark blue	R
NCDO2118_RS04085	3,726969783	107 dark blue	I
NCDO2118_RS04260	3,725653081	108 dark blue	D
NCDO2118_RS12170	3,723237721	109 dark blue	E
NCDO2118_RS00105	3,72186563	110 dark blue	IQ
NCDO2118_RS06445	3,721116128	111 dark blue	Q
NCDO2118_RS11430	3,715358061	112 dark blue	J
NCDO2118_RS06115	3,711650731	113 dark blue	M
NCDO2118_RS09945	3,697453551	114 dark blue	C
NCDO2118_RS09715	3,695193447	115 dark blue	C
NCDO2118_RS09035	3,69458389	116 dark blue	L
NCDO2118_RS02160	3,693289088	117 dark blue	J
NCDO2118_RS00820	3,686990794	118 dark blue	J
NCDO2118_RS03000	3,684106363	119 dark blue	J
NCDO2118_RS02020	3,682221305	120 dark blue	M
NCDO2118_RS01890	3,680425178	121 dark blue	S
NCDO2118_RS09400	3,673849163	122 dark blue	J
NCDO2118_RS10410	3,673023267	123 dark blue	J
NCDO2118_RS04050	3,672314414	124 dark blue	G
NCDO2118_RS02515	3,670330857	125 dark blue	F
NCDO2118_RS12275	3,668515009	126 dark blue	O
NCDO2118_RS05310	3,668468123	127 dark blue	O
NCDO2118_RS09635	3,653637472	128 dark blue	E
NCDO2118_RS02265	3,653167733	129 dark blue	O
NCDO2118_RS09545	3,651075974	130 dark blue	C
NCDO2118_RS11535	3,650695635	131 dark blue	E
NCDO2118_RS12165	3,646815793	132 dark blue	S
NCDO2118_RS07565	3,633112639	133 dark blue	E
NCDO2118_RS04025	3,631103788	134 dark blue	K
NCDO2118_RS00800	3,625340457	135 dark blue	K
NCDO2118_RS01805	3,620722323	136 dark blue	O
NCDO2118_RS04065	3,618320602	137 dark blue	IQ
NCDO2118_RS11400	3,617677286	138 dark blue	J
NCDO2118_RS11155	3,613848534	139 dark blue	E
NCDO2118_RS00970	3,612281347	140 dark blue	S
NCDO2118_RS05355	3,611864409	141 dark blue	S
NCDO2118_RS09460	3,610768337	142 dark blue	P
NCDO2118_RS01385	3,605600587	143 dark blue	F
NCDO2118_RS05615	3,602737341	144 dark blue	S
NCDO2118_RS10900	3,602592868	145 dark blue	G
NCDO2118_RS11125	3,60083814	146 dark blue	P

NCDO2118_RS01105	3,590195577	147 dark blue	S
NCDO2118_RS11520	3,583859929	148 dark blue	J
NCDO2118_RS00370	3,582656454	149 dark blue	R
NCDO2118_RS01735	3,580373308	150 dark blue	R
NCDO2118_RS11865	3,570010166	151 dark blue	G
NCDO2118_RS11900	3,567581242	152 dark blue	S
NCDO2118_RS09935	3,565348042	153 dark blue	E
NCDO2118_RS11040	3,558812645	154 dark blue	J
NCDO2118_RS10720	3,558700255	155 dark blue	F
NCDO2118_RS00995	3,553122827	156 dark blue	M
NCDO2118_RS01725	3,551624922	157 dark blue	F
NCDO2118_RS11890	3,55115758	158 dark blue	J
NCDO2118_RS04310	3,540337311	159 dark blue	C
NCDO2118_RS00985	3,538942961	160 dark blue	M
NCDO2118_RS02055	3,536774296	161 dark blue	E
NCDO2118_RS12295	3,524777306	162 dark blue	E
NCDO2118_RS10595	3,521614746	163 dark blue	J
NCDO2118_RS11255	3,520590984	164 dark blue	ET
NCDO2118_RS00250	3,514701279	165 dark blue	J
NCDO2118_RS05535	3,505712408	166 dark blue	M
NCDO2118_RS07500	3,503632714	167 dark blue	F
NCDO2118_RS11385	3,50220697	168 dark blue	J
NCDO2118_RS08160	3,501740915	169 dark blue	E
NCDO2118_RS10125	3,490660461	170 dark blue	J
NCDO2118_RS10300	3,489091895	171 dark blue	J
NCDO2118_RS05725	3,484792	172 dark blue	S
NCDO2118_RS05785	3,481615999	173 dark blue	J
NCDO2118_RS05390	3,477609817	174 dark blue	J
NCDO2118_RS03355	3,477412767	175 dark blue	C
NCDO2118_RS09500	3,477247926	176 dark blue	O
NCDO2118_RS00445	3,476863545	177 dark blue	U
NCDO2118_RS02065	3,466137058	178 dark blue	LKJ
NCDO2118_RS07510	3,465084718	179 dark blue	F
NCDO2118_RS04110	3,461281855	180 dark blue	E
NCDO2118_RS04265	3,457761947	181 dark blue	F
NCDO2118_RS11115	3,456422365	182 dark blue	KG
NCDO2118_RS08445	3,452524338	183 dark blue	TK
NCDO2118_RS03135	3,447102227	184 dark blue	G
NCDO2118_RS10195	3,442333953	185 dark blue	J
NCDO2118_RS08185	3,431728916	186 dark blue	S
NCDO2118_RS03320	3,430260515	187 dark blue	I
NCDO2118_RS11175	3,427873775	188 dark blue	E
NCDO2118_RS09820	3,424120525	189 dark blue	R
NCDO2118_RS11260	3,419455518	190 dark blue	F
NCDO2118_RS07220	3,419360749	191 dark blue	M
NCDO2118_RS11055	3,419317733	192 dark blue	C
NCDO2118_RS12290	3,416252815	193 dark blue	R
NCDO2118_RS12215	3,40432866	194 dark blue	L
NCDO2118_RS03280	3,403690919	195 dark blue	K
NCDO2118_RS05720	3,388610143	196 dark blue	J
NCDO2118_RS12470	3,386033514	197 dark blue	J
NCDO2118_RS08410	3,384319529	198 dark blue	R
NCDO2118_RS00275	3,383478016	199 dark blue	R
NCDO2118_RS05415	3,378015869	200 dark blue	F
NCDO2118_RS02220	3,375951724	201 dark blue	J
NCDO2118_RS10495	3,364619865	202 dark blue	H
NCDO2118_RS11730	3,363293002	203 dark blue	L
NCDO2118_RS11665	3,360891131	204 dark blue	J
NCDO2118_RS08810	3,360489167	205 dark blue	F
NCDO2118_RS01380	3,358631157	206 dark blue	M
NCDO2118_RS05620	3,358603792	207 dark blue	S
NCDO2118_RS03115	3,355217681	208 dark blue	T
NCDO2118_RS04380	3,352000829	209 dark blue	E
NCDO2118_RS11200	3,351524496	210 dark blue	S
NCDO2118_RS00810	3,347189471	211 dark blue	J
NCDO2118_RS11075	3,346908128	212 dark blue	T
NCDO2118_RS04890	3,345093267	213 dark blue	F
NCDO2118_RS02060	3,343441194	214 dark blue	J
NCDO2118_RS05855	3,339931129	215 dark blue	C
NCDO2118_RS00335	3,339470519	216 dark blue	J
NCDO2118_RS06870	3,338371374	217 dark blue	L
NCDO2118_RS03845	3,338209189	218 dark blue	H
NCDO2118_RS06050	3,336893146	219 dark blue	E
NCDO2118_RS00365	3,329180426	220 dark blue	S

NCDO2118_RS04570	3,321405446	221 dark blue	J
NCDO2118_RS10080	3,319655245	222 dark blue	L
NCDO2118_RS01810	3,318678306	223 dark blue	E
NCDO2118_RS08455	3,316780045	224 dark blue	J
NCDO2118_RS01850	3,313823096	225 dark blue	V
NCDO2118_RS10650	3,312910523	226 dark blue	G
NCDO2118_RS09010	3,30750815	227 dark blue	F
NCDO2118_RS09055	3,306586796	228 dark blue	E
NCDO2118_RS02560	3,306280198	229 dark blue	S
NCDO2118_RS04370	3,305057254	230 dark blue	L
NCDO2118_RS04495	3,299969705	231 dark blue	K
NCDO2118_RS10400	3,296897305	232 dark blue	M
NCDO2118_RS02230	3,294312158	233 dark blue	G
NCDO2118_RS11000	3,291332731	234 dark blue	J
NCDO2118_RS12265	3,287777172	235 dark blue	G
NCDO2118_RS11245	3,287372365	236 dark blue	R
NCDO2118_RS00795	3,286937648	237 dark blue	E
NCDO2118_RS02825	3,284700862	238 dark blue	J
NCDO2118_RS02820	3,283538999	239 dark blue	G
NCDO2118_RS02855	3,280950517	240 dark blue	I
NCDO2118_RS00955	3,280359714	241 dark blue	R
NCDO2118_RS06465	3,279516459	242 dark blue	J
NCDO2118_RS01130	3,278690327	243 dark blue	TQ
NCDO2118_RS09030	3,27695931	244 dark blue	OC
NCDO2118_RS10805	3,276642751	245 dark blue	J
NCDO2118_RS03875	3,26767066	246 dark blue	S
NCDO2118_RS04185	3,258341585	247 dark blue	R
NCDO2118_RS01095	3,246244211	248 dark blue	S
NCDO2118_RS07135	3,244988353	249 dark blue	H
NCDO2118_RS08225	3,244614435	250 dark blue	I
NCDO2118_RS04755	3,242384907	251 dark blue	L
NCDO2118_RS12010	3,242284941	252 dark blue	L
NCDO2118_RS00430	3,237620544	253 dark blue	I
NCDO2118_RS11485	3,235762302	254 dark blue	J
NCDO2118_RS09185	3,233533862	255 dark blue	HI
NCDO2118_RS10950	3,231606396	256 dark blue	O
NCDO2118_RS07215	3,229907229	257 dark blue	C
NCDO2118_RS03230	3,229890394	258 dark blue	O
NCDO2118_RS11045	3,229674649	259 dark blue	F
NCDO2118_RS05420	3,22691982	260 dark blue	F
NCDO2118_RS01740	3,223203103	261 dark blue	S
NCDO2118_RS05215	3,220925258	262 dark blue	F
NCDO2118_RS05755	3,213730728	263 dark blue	H
NCDO2118_RS11205	3,21278877	264 dark blue	KL
NCDO2118_RS01115	3,212292686	265 dark blue	R
NCDO2118_RS03790	3,209150665	266 dark blue	R
NCDO2118_RS02810	3,208531729	267 dark blue	O
NCDO2118_RS10270	3,208507209	268 dark blue	FE
NCDO2118_RS12510	3,197321785	269 dark blue	J
NCDO2118_RS08425	3,193706831	270 dark blue	M
NCDO2118_RS07235	3,190976825	271 dark blue	H
NCDO2118_RS01715	3,188558979	272 dark blue	K
NCDO2118_RS02395	3,185895641	273 dark blue	L
NCDO2118_RS06810	3,185422921	274 dark blue	F
NCDO2118_RS05995	3,185137125	275 dark blue	R
NCDO2118_RS11315	3,184809133	276 dark blue	O
NCDO2118_RS10880	3,180847942	277 dark blue	G
NCDO2118_RS08020	3,178895147	278 dark blue	F
NCDO2118_RS01020	3,178178758	279 dark blue	G
NCDO2118_RS02190	3,178039063	280 dark blue	H
NCDO2118_RS03085	3,177904927	281 dark blue	J
NCDO2118_RS11065	3,175726585	282 dark blue	L
NCDO2118_RS04535	3,174758718	283 dark blue	M
NCDO2118_RS05405	3,174495087	284 dark blue	D
NCDO2118_RS05545	3,171434905	285 dark blue	R
NCDO2118_RS06815	3,163836058	286 dark blue	M
NCDO2118_RS07200	3,159671442	287 dark blue	M
NCDO2118_RS04075	3,157166927	288 dark blue	IQR
NCDO2118_RS04105	3,155634546	289 dark blue	I
NCDO2118_RS02245	3,154820218	290 dark blue	R
NCDO2118_RS01855	3,148847181	291 dark blue	M
NCDO2118_RS09095	3,146641756	292 dark blue	R
NCDO2118_RS08830	3,146125723	293 dark blue	E
NCDO2118_RS11230	3,140781057	294 dark blue	S

NCDO2118_RS07490	3,140096143	295	dark blue	M
NCDO2118_RS11120	3,139969272	296	dark blue	P
NCDO2118_RS11195	3,134581269	297	dark blue	M
NCDO2118_RS03375	3,131617893	298	dark blue	OU
NCDO2118_RS10205	3,127145445	299	dark blue	J
NCDO2118_RS00225	3,124787592	300	dark blue	C
NCDO2118_RS10375	3,118483166	301	dark blue	E
NCDO2118_RS09895	3,111191807	302	dark blue	R
NCDO2118_RS09060	3,108068949	303	dark blue	S
NCDO2118_RS01000	3,106206865	304	dark blue	M
NCDO2118_RS01120	3,10425884	305	dark blue	K
NCDO2118_RS08990	3,103992115	306	dark blue	G
NCDO2118_RS00215	3,102406916	307	dark blue	C
NCDO2118_RS04020	3,101386413	308	dark blue	S
NCDO2118_RS10240	3,099441112	309	dark blue	J
NCDO2118_RS05245	3,09825572	310	dark blue	O
NCDO2118_RS01160	3,098113599	311	dark blue	S
NCDO2118_RS07205	3,097866368	312	dark blue	P
NCDO2118_RS09075	3,091054676	313	dark blue	D
NCDO2118_RS04070	3,088892522	314	dark blue	I
NCDO2118_RS04690	3,088395927	315	dark blue	R
NCDO2118_RS05800	3,084398507	316	dark blue	L
NCDO2118_RS05590	3,08116936	317	dark blue	F
NCDO2118_RS05585	3,080461677	318	dark blue	F
NCDO2118_RS00235	3,079708226	319	dark blue	H
NCDO2118_RS07165	3,077664074	320	dark blue	F
NCDO2118_RS01900	3,07332321	321	dark blue	P
NCDO2118_RS09115	3,073015299	322	dark blue	R
NCDO2118_RS05865	3,071755025	323	dark blue	J
NCDO2118_RS01365	3,071240394	324	dark blue	Q
NCDO2118_RS04160	3,070954164	325	dark blue	L
NCDO2118_RS10690	3,070857069	326	dark blue	J
NCDO2118_RS00110	3,069093165	327	dark blue	O
NCDO2118_RS11480	3,067575448	328	dark blue	J
NCDO2118_RS02525	3,065443106	329	dark blue	L
NCDO2118_RS12235	3,062508374	330	dark blue	I
NCDO2118_RS03270	3,059157114	331	dark blue	K
NCDO2118_RS12320	3,057899017	332	dark blue	G
NCDO2118_RS08450	3,053000689	333	dark blue	R
NCDO2118_RS05400	3,052559988	334	dark blue	J
NCDO2118_RS04250	3,04675723	335	dark blue	S
NCDO2118_RS03275	3,04312219	336	dark blue	S
NCDO2118_RS03130	3,03855044	337	dark blue	S
NCDO2118_RS02025	3,03810733	338	dark blue	M
NCDO2118_RS05640	3,03500491	339	dark blue	J
NCDO2118_RS09025	3,033516723	340	dark blue	S
NCDO2118_RS12465	3,03105759	341	dark blue	J
NCDO2118_RS09910	3,030201144	342	dark blue	M
NCDO2118_RS07170	3,029510414	343	dark blue	R
NCDO2118_RS00385	3,027694892	344	dark blue	J
NCDO2118_RS03030	3,025294604	345	dark blue	E
NCDO2118_RS11920	3,022549154	346	dark blue	J
NCDO2118_RS12020	3,021142001	347	dark blue	T
NCDO2118_RS09380	3,020334558	348	dark blue	G
NCDO2118_RS00605	3,020164831	349	dark blue	R
NCDO2118_RS10960	3,017002169	350	dark blue	J
NCDO2118_RS08880	3,016942133	351	dark blue	K
NCDO2118_RS06460	3,015992925	352	dark blue	L
NCDO2118_RS01845	3,014601933	353	dark blue	V
NCDO2118_RS11785	3,012346489	354	dark blue	G
NCDO2118_RS10425	3,010703577	355	dark blue	J
NCDO2118_RS11280	3,009237186	356	dark blue	KR
NCDO2118_RS11495	3,007827521	357	dark blue	J
NCDO2118_RS06150	3,007175361	358	dark blue	D
NCDO2118_RS01920	3,004436897	359	dark blue	P
NCDO2118_RS06820	2,99996942	360	light blue	S
NCDO2118_RS12490	2,997757029	361	light blue	U
NCDO2118_RS02260	2,995705219	362	light blue	O
NCDO2118_RS08210	2,995104461	363	light blue	R
NCDO2118_RS06780	2,991912551	364	light blue	J
NCDO2118_RS08230	2,990911939	365	light blue	I
NCDO2118_RS02675	2,990854572	366	light blue	G
NCDO2118_RS06125	2,988523936	367	light blue	J
NCDO2118_RS04095	2,985274599	368	light blue	I

NCDO2118_RS02425	2,984884825	369	light blue	G
NCDO2118_RS11880	2,982323779	370	light blue	K
NCDO2118_RS07660	2,981518283	371	light blue	S
NCDO2118_RS10280	2,973393688	372	light blue	E
NCDO2118_RS09840	2,972689896	373	light blue	O
NCDO2118_RS08065	2,972556481	374	light blue	F
NCDO2118_RS02085	2,972261371	375	light blue	L
NCDO2118_RS05990	2,971698905	376	light blue	O
NCDO2118_RS00450	2,970322119	377	light blue	E
NCDO2118_RS02635	2,969484126	378	light blue	E
NCDO2118_RS03935	2,968483961	379	light blue	S
NCDO2118_RS04035	2,967639284	380	light blue	J
NCDO2118_RS02070	2,960052838	381	light blue	R
NCDO2118_RS11030	2,956172667	382	light blue	S
NCDO2118_RS09355	2,95478375	383	light blue	F
NCDO2118_RS08140	2,953010152	384	light blue	V
NCDO2118_RS10965	2,949012729	385	light blue	P
NCDO2118_RS03950	2,937605898	386	light blue	K
NCDO2118_RS04145	2,93442014	387	light blue	P
NCDO2118_RS00625	2,930680424	388	light blue	L
NCDO2118_RS10660	2,929618973	389	light blue	S
NCDO2118_RS12285	2,929001864	390	light blue	M
NCDO2118_RS12180	2,924537478	391	light blue	T
NCDO2118_RS07180	2,923719702	392	light blue	C
NCDO2118_RS05770	2,920535674	393	light blue	R
NCDO2118_RS10395	2,919861186	394	light blue	LR
NCDO2118_RS09385	2,915031332	395	light blue	S
NCDO2118_RS10285	2,91483237	396	light blue	S
NCDO2118_RS09475	2,914416164	397	light blue	P
NCDO2118_RS12325	2,913012664	398	light blue	T
NCDO2118_RS12205	2,912579183	399	light blue	L
NCDO2118_RS07380	2,908072997	400	light blue	T
NCDO2118_RS06065	2,906432861	401	light blue	E
NCDO2118_RS03250	2,900938279	402	light blue	J
NCDO2118_RS07345	2,894102527	403	light blue	S
NCDO2118_RS04120	2,890051703	404	light blue	S
NCDO2118_RS04350	2,887990391	405	light blue	J
NCDO2118_RS11560	2,886959533	406	light blue	M
NCDO2118_RS08420	2,878847872	407	light blue	M
NCDO2118_RS04525	2,875769334	408	light blue	M
NCDO2118_RS10345	2,87273345	409	light blue	J
NCDO2118_RS10935	2,869197427	410	light blue	O
NCDO2118_RS00885	2,867871972	411	light blue	F
NCDO2118_RS01360	2,867439253	412	light blue	R
NCDO2118_RS04305	2,863623998	413	light blue	C
NCDO2118_RS10925	2,861894309	414	light blue	H
NCDO2118_RS00755	2,860372218	415	light blue	J
NCDO2118_RS00965	2,85730065	416	light blue	J
NCDO2118_RS06845	2,849649159	417	light blue	E
NCDO2118_RS06665	2,848559499	418	light blue	E
NCDO2118_RS12210	2,847127533	419	light blue	L
NCDO2118_RS10145	2,841439905	420	light blue	L
NCDO2118_RS12030	2,8344281	421	light blue	S
NCDO2118_RS10170	2,833362355	422	light blue	E
NCDO2118_RS10710	2,832422675	423	light blue	G
NCDO2118_RS05835	2,832251801	424	light blue	L
NCDO2118_RS10130	2,830983491	425	light blue	S
NCDO2118_RS12495	2,826156674	426	light blue	F
NCDO2118_RS07560	2,825807085	427	light blue	E
NCDO2118_RS00010	2,820946053	428	light blue	L
NCDO2118_RS03385	2,820628164	429	light blue	P
NCDO2118_RS10990	2,820467374	430	light blue	S
NCDO2118_RS09395	2,816721655	431	light blue	S
NCDO2118_RS01680	2,81653396	432	light blue	OC
NCDO2118_RS03025	2,81617094	433	light blue	J
NCDO2118_RS11110	2,81170289	434	light blue	S
NCDO2118_RS03675	2,81134806	435	light blue	K
NCDO2118_RS08155	2,803882363	436	light blue	S
NCDO2118_RS10520	2,799989489	437	light blue	S
NCDO2118_RS10420	2,799715951	438	light blue	T
NCDO2118_RS11595	2,797812341	439	light blue	P
NCDO2118_RS04090	2,790961075	440	light blue	I
NCDO2118_RS04595	2,789159458	441	light blue	T
NCDO2118_RS00205	2,788294894	442	light blue	E

NCDO2118_RS02955	2,785411558	443	light blue	S
NCDO2118_RS06020	2,780723708	444	light blue	Q
NCDO2118_RS04045	2,779776825	445	light blue	J
NCDO2118_RS05745	2,773331594	446	light blue	H
NCDO2118_RS05335	2,7592456	447	light blue	G
NCDO2118_RS10265	2,756587931	448	light blue	S
NCDO2118_RS11680	2,755994195	449	light blue	I
NCDO2118_RS11705	2,755016048	450	light blue	M
NCDO2118_RS03290	2,754165071	451	light blue	O
NCDO2118_RS11020	2,752469395	452	light blue	S
NCDO2118_RS04170	2,746828675	453	light blue	S
NCDO2118_RS10405	2,745522528	454	light blue	E
NCDO2118_RS02315	2,742238151	455	light blue	I
NCDO2118_RS09450	2,736107514	456	light blue	P
NCDO2118_RS01860	2,732153629	457	light blue	R
NCDO2118_RS07310	2,731174505	458	light blue	S
NCDO2118_RS10450	2,725283807	459	light blue	J
NCDO2118_RS03655	2,725013226	460	light blue	J
NCDO2118_RS10840	2,724681315	461	light blue	S
NCDO2118_RS04175	2,724121018	462	light blue	IQR
NCDO2118_RS08355	2,720933575	463	light blue	J
NCDO2118_RS05430	2,717951555	464	light blue	O
NCDO2118_RS05775	2,717576863	465	light blue	M
NCDO2118_RS02250	2,715595893	466	light blue	R
NCDO2118_RS04100	2,715361634	467	light blue	I
NCDO2118_RS05940	2,710424432	468	light blue	S
NCDO2118_RS08845	2,702851295	469	light blue	S
NCDO2118_RS03045	2,696985352	470	light blue	HE
NCDO2118_RS04775	2,695344557	471	light blue	K
NCDO2118_RS09570	2,692405967	472	light blue	J
NCDO2118_RS11700	2,690494322	473	light blue	O
NCDO2118_RS07470	2,69029821	474	light blue	TK
NCDO2118_RS05660	2,683316097	475	light blue	T
NCDO2118_RS09700	2,682543016	476	light blue	C
NCDO2118_RS04230	2,67387256	477	light blue	S
NCDO2118_RS00255	2,670328009	478	light blue	O
NCDO2118_RS01345	2,668455861	479	light blue	R
NCDO2118_RS03930	2,668162406	480	light blue	L
NCDO2118_RS11600	2,664953942	481	light blue	S
NCDO2118_RS12240	2,664623566	482	light blue	S
NCDO2118_RS02875	2,661704872	483	light blue	U
NCDO2118_RS00280	2,656998532	484	light blue	U
NCDO2118_RS09595	2,656229573	485	light blue	E
NCDO2118_RS11005	2,650268398	486	light blue	J
NCDO2118_RS08415	2,645357324	487	light blue	D
NCDO2118_RS05750	2,629930201	488	light blue	KR
NCDO2118_RS09935	2,628587377	489	light blue	O
NCDO2118_RS10610	2,620302305	490	light blue	C
NCDO2118_RS10705	2,610054537	491	light blue	K
NCDO2118_RS11875	2,602146004	492	light blue	MG
NCDO2118_RS00960	2,601745531	493	light blue	S
NCDO2118_RS10275	2,599165581	494	light blue	T
NCDO2118_RS08795	2,599042611	495	light blue	EF
NCDO2118_RS00975	2,592727366	496	light blue	M
NCDO2118_RS00900	2,589223434	497	light blue	F
NCDO2118_RS09175	2,588573339	498	light blue	G
NCDO2118_RS00790	2,585870685	499	light blue	S
NCDO2118_RS07255	2,580430102	500	light blue	F
NCDO2118_RS08175	2,578295137	501	light blue	E
NCDO2118_RS09845	2,576338375	502	light blue	O
NCDO2118_RS05350	2,574123663	503	light blue	R
NCDO2118_RS06875	2,569365969	504	light blue	J
NCDO2118_RS12365	2,553614442	505	light blue	O
NCDO2118_RS11190	2,553278408	506	light blue	K
NCDO2118_RS00910	2,550545055	507	light blue	R
NCDO2118_RS08900	2,545365585	508	light blue	E
NCDO2118_RS01830	2,537293986	509	light blue	K
NCDO2118_RS05945	2,517592077	510	light blue	M
NCDO2118_RS05955	2,517415169	511	light blue	F
NCDO2118_RS08215	2,508937048	512	light blue	J
NCDO2118_RS02015	2,507761998	513	light blue	L
NCDO2118_RS12310	2,505797828	514	light blue	D
NCDO2118_RS10695	2,500943851	515	light blue	J
NCDO2118_RS08200	2,500374481	516	light blue	I

NCDO2118_RS10975	2,490259581	517	light blue	U
NCDO2118_RS09195	2,478920263	518	light blue	L
NCDO2118_RS03910	2,478413943	519	light blue	K
NCDO2118_RS11725	2,467190603	520	light blue	R
NCDO2118_RS04465	2,461958762	521	light blue	H
NCDO2118_RS00310	2,45796449	522	light blue	T
NCDO2118_RS03945	2,449546666	523	light blue	R
NCDO2118_RS10855	2,448238076	524	light blue	S
NCDO2118_RS00400	2,447006494	525	light blue	TK
NCDO2118_RS02405	2,429794145	526	light blue	S
NCDO2118_RS06785	2,422917252	527	light blue	K
NCDO2118_RS04685	2,414608711	528	light blue	TK
NCDO2118_RS07155	2,41163729	529	light blue	K
NCDO2118_RS05235	2,4046763	530	light blue	E
NCDO2118_RS06790	2,401616185	531	light blue	L
NCDO2118_RS10340	2,398915156	532	light blue	S
NCDO2118_RS08890	2,395143725	533	light blue	EM
NCDO2118_RS09535	2,385563309	534	light blue	R
NCDO2118_RS02115	2,385009499	535	light blue	S
NCDO2118_RS11830	2,369495442	536	light blue	D
NCDO2118_RS07485	2,368990815	537	light blue	K
NCDO2118_RS10640	2,363971167	538	light blue	LR
NCDO2118_RS11470	2,361672389	539	light blue	J
NCDO2118_RS08120	2,352741889	540	light blue	F
NCDO2118_RS12040	2,341712101	541	light blue	S
NCDO2118_RS01170	2,338191957	542	light blue	L
NCDO2118_RS01965	2,335586358	543	light blue	P
NCDO2118_RS03925	2,31377089	544	light blue	J
NCDO2118_RS09455	2,310623255	545	light blue	P
NCDO2118_RS11710	2,31059516	546	light blue	M
NCDO2118_RS11150	2,30015023	547	light blue	E
NCDO2118_RS07515	2,29403177	548	light blue	J
NCDO2118_RS03265	2,282327668	549	light blue	L
NCDO2118_RS01745	2,281689389	550	light blue	O
NCDO2118_RS03765	2,28029006	551	light blue	V
NCDO2118_RS11670	2,266756908	552	light blue	M
NCDO2118_RS02200	2,25556152	553	light blue	C
NCDO2118_RS05555	2,252505802	554	light blue	V
NCDO2118_RS10600	2,24925083	555	light blue	S
NCDO2118_RS08800	2,249143021	556	light blue	F
NCDO2118_RS00350	2,245108234	557	light blue	K
NCDO2118_RS04225	2,23731209	558	light blue	M
NCDO2118_RS01685	2,20853212	559	light blue	E
NCDO2118_RS00920	2,178681194	560	light blue	H
NCDO2118_RS05715	2,168452984	561	light blue	J
NCDO2118_RS12390	2,165741912	562	light blue	S
NCDO2118_RS06760	2,160520267	563	light blue	K
NCDO2118_RS10385	2,124574047	564	light blue	F
NCDO2118_RS03640	2,093280844	565	light blue	L
NCDO2118_RS02680	2,064879655	566	light blue	M
NCDO2118_RS05205	2,064771637	567	light blue	S
NCDO2118_RS09655	2,04071531	568	light blue	E
NCDO2118_RS07110	2,038380593	569	light blue	P
NCDO2118_RS11320	2,035707015	570	light blue	P
NCDO2118_RS04115	2,024865243	571	light blue	E
NCDO2118_RS09110	2,021180265	572	light blue	F
NCDO2118_RS10325	2,017151463	573	light blue	R
NCDO2118_RS00915	2,012729258	574	light blue	C
NCDO2118_RS12360	1,988944839	575	Green	S
NCDO2118_RS02890	1,951723334	576	Green	H
NCDO2118_RS09575	1,938114972	577	Green	L
NCDO2118_RS00855	1,91798435	578	Green	R
NCDO2118_RS09045	1,917158453	579	Green	O
NCDO2118_RS02850	1,893246923	580	Green	I
NCDO2118_RS07570	1,870829311	581	Green	P
NCDO2118_RS05275	1,861379086	582	Green	T
NCDO2118_RS05735	1,724962352	583	Green	H
NCDO2118_RS05885	1,711100489	584	Green	M
NCDO2118_RS04390	1,613936268	585	Green	M
NCDO2118_RS05210	1,546671613	586	Green	S

COG groups are defined in the legend to Fig. 2B.

Tag = colors of the proteins presents in the Dynamic Range
