# Supplementary material for: Comparative proteomic analysis of four biotechnological strains Lactococcus lactis through label‐free quantitative proteomics
Source: Microb Biotechnol. 2018 Oct 19;12(2):265–74. doi: 10.1111/1751-7915.13305 (PMC6389847; doi:10.1111/1751-7915.13305)
Supplement: Supplementary file 4 — Table S3. Total list of differentially expressed proteins. [file MBT2-12-265-s004.pdf]

Supplementary File 6: Total list of differentially expressed proteins

MG1363	NZ9000	NCDO2118	IL1403	Description	COG Function	Log2_MG:NC	Log2_IL:NC	Log2_NZ:NC	Anova (p)
llmg_2069	LLNZ_10650	NCDO2118_RS10375	L130687	peptidase PepC protein	E	4,245018118	4,298323638	5,009281113	0,002616763
llmg_1994	LLNZ_10290	NCDO2118_RS09935	L60596	Peptidase T	E	0,058946723	-0,773517029	0,507633639	0,047967391
llmg_1328	LLNZ_06870	NCDO2118_RS06050	L175357	Spermidine/putrescine import ATP-binding protein PotA	E	-4,120542351	-1,264169161	-2,597535764	0,04145831
llmg_1969	LLNZ_10165	NCDO2118_RS09820	L29491	Fe-S cluster assembly protein SufB	R	-2,852135996	-1,401468054	-2,651561497	0,021312553
llmg_2048	LLNZ_10550	NCDO2118_RS10280	L111126	Cysteine desulfurase	E	-0,586943909	-3,548972575	0,922251142	0,038625469
llmg_2235	LLNZ_11520	NCDO2118_RS10900	L0028	Galactokinase	G	-0,50191831	1,791075935	-0,309917605	0,015183043
llmg_0446	LLNZ_02310	NCDO2118_RS02405	L27865	Sugar ABC transporter ATP binding protein	S	-2,264194154	1,076708469	-1,312010084	0,009909341
llmg_2539	LLNZ_13115	NCDO2118_RS12385	L0005	Glyceraldehyde-3-phosphate dehydrogenase	G	1,592188236	-2,129932416	1,499888395	0,002783779
llmg_0214	LLNZ_01115	NCDO2118_RS01020	L4342	ABC transporter ATP-binding protein	G	2,248058561	-3,515273511	3,295331006	0,039129552
llmg_2055	LLNZ_10580	NCDO2118_RS10305	L117685	Cell division protein DivIVA	D	-0,498898309	0,737793687	1,116805916	0,005786312
llmg_2061	LLNZ_10610	NCDO2118_RS10335	L0207	Cell division protein FtsA	D	-2,902242808	-0,280668382	-1,736651262	0,006312253
llmg_0517	LLNZ_02670	NCDO2118_RS02680	L134243	UDP-N-acetylglucosamine 1-carboxyvinyltransferase	M	-1,597796766	1,381150559	-0,859497542	0,014014267
llmg_0912	LLNZ_04685	NCDO2118_RS08425	L0237	UDP-N-acetylmuramoylalanine--D-glutamate ligase	M	-3,522371384	-2,135635522	-2,826368914	0,048342688
llmg_1831	LLNZ_09420	NCDO2118_RS03845	L0171	1,4-dihydroxy-2-naphthoyl-CoA synthase	H	7,89119687	3,528382739	6,693372648	0,037823562
llmg_1735	LLNZ_08960	NCDO2118_RS04305	L39857	NADH dehydrogenase	C	7,494248851	2,786953776	7,180845593	0,010293924
llmg_0408	LLNZ_02125	NCDO2118_RS02250	L196579	NADH oxidase	R	0,658850464	1,445905247	1,98312957	0,006992493
llmg_1597	LLNZ_08215	NCDO2118_RS05205	L158463	Hypothetical protein	S	-0,579372154	2,216887878	-0,226522709	0,026001538
llmg_1973	LLNZ_10185	NCDO2118_RS09840	L33412	Fe-S cluster assembly protein SufD	O	0,403802438	0,768188316	1,285478191	0,020908927
llmg_2225	LLNZ_11470	NCDO2118_RS10840	L30285	XRE family transcriptional regulator	S	1,585943021	-2,880176387	0,456661339	0,038126339
llmg_0196	LLNZ_01020	NCDO2118_RS00920	L186258	Prenyl transferase	H	-0,855093391	5,497413274	0,186466095	0,020859474
llmg_0765	LLNZ_03980	NCDO2118_RS09095	L106425	Hypothetical protein	R	1,663462364	2,344202041	1,67978858	0,029436486
llmg_1497	LLNZ_07705	NCDO2118_RS05620	L86251	Sugar phosphate phosphatase	S	3,896671736	1,54042727	3,03442378	0,029152666
llmg_1110	LLNZ_05740	NCDO2118_RS07235	L179531	heptaprenyl diphosphate synthase subunit II	H	2,145304976	-0,024382659	1,490839601	0,018798097
llmg_1116	LLNZ_05770	NCDO2118_RS07205	L174389	Tellurite resistance protein	P	-1,918564765	-0,833362314	-0,057180312	0,037798086
llmg_0335	LLNZ_01760	NCDO2118_RS01900	L117444	Lipoprotein	P	7,649379278	4,265961453	6,269278254	0,027265641
llmg_1896	LLNZ_09765	NCDO2118_RS09455	L154481	Phosphate import ATP-binding protein PstB 1	P	2,169472355	4,013873274	3,219996997	0,047063539
llmg_2302	LLNZ_11895	NCDO2118_RS11125	L85575	Ferritin	P	2,436646258	2,498943679	3,618335514	0,036570261
llmg_0341	LLNZ_01785	NCDO2118_RS01920	L121289	Methionine import ATP-binding protein	P	2,253559357	1,380664464	2,291880501	0,017495556
llmg_2488	LLNZ_12855	NCDO2118_RS12210	L0266	Holliday junction DNA helicase RuvA	L	-5,057039797	-0,85360375	-3,61614219	0,020559156
llmg_0496	LLNZ_02565	NCDO2118_RS02580	L102317	DNA-binding protein HU	L	-2,975725775	-2,754662724	-1,411287532	0,0378037
llmg_1275	LLNZ_06580	NCDO2118_RS06445	L0321	Alpha-acetolactate decarboxylase	Q	3,654622162	-0,016222057	1,444690597	0,045977006
llmg_2080	LLNZ_10700	NCDO2118_RS10420	L140754	Protein phosphatase	T	-1,367188503	1,805798699	1,599166247	0,0266092
llmg_2047	LLNZ_10545	NCDO2118_RS10275	L110467	Hypothetical protein	T	-2,443876664	-4,786563526	-1,918441525	0,035452487
llmg_1796	LLNZ_09250	NCDO2118_RS04025	L173813	Transcription termination factor NusA	K	3,432662785	1,692853896	2,732867763	0,045928212
llmg_2401	LLNZ_12410	NCDO2118_RS11600	L168265	MarR family transcriptional regulator	S	5,873817775	1,429209022	2,207363175	0,004461648
llmg_1878	LLNZ_09675	NCDO2118_RS03675	L92686	nitrogen utilization protein B	K	1,649375794	-1,789419392	1,957926379	0,000541433
llmg_2455	LLNZ_12695	NCDO2118_RS11890	L0351	Valyl-tRNA synthetase	J	2,490301327	1,70891453	0,611153618	0,030213194
llmg_2362	LLNZ_12205	NCDO2118_RS11415	L0411	50S ribosomal protein L15	J	-0,728481884	0,568038791	-0,258239759	0,044085396
llmg_2378	LLNZ_12285	NCDO2118_RS11490	L0418	50S ribosomal protein L22	J	2,722852002	0,406945611	2,414045526	0,002514715
llmg_2366	LLNZ_12225	NCDO2118_RS11435	L0404	50S ribosomal protein L6	J	-0,678230801	0,098834855	0,407963714	0,000994217

COG groups are defined in the legend to Fig. 2B.
