# Supplementary material for: Comparative proteomic analysis of four biotechnological strains Lactococcus lactis through label‐free quantitative proteomics
Source: Microb Biotechnol. 2018 Oct 19;12(2):265–74. doi: 10.1111/1751-7915.13305 (PMC6389847; doi:10.1111/1751-7915.13305)
Supplement: Supplementary file 5 — Table S4. KEGG pathway enrichment analysis. [file MBT2-12-265-s005.pdf]

Supplementary File 7: KEGG pathway enrichment analysis.

Kegg Pathway	P-Value	Corrected P-Value
Ribosome	6,66E-07	5,53E-05
Metabolic pathways	1,02E-04	4,23E-03
Pyruvate metabolism	2,63E-04	1,99E-03
Microbial metabolism in diverse environments	6,93E-04	4,79E-03
Pentose phosphate pathway	8,48E-04	5,40E-03
Nucleotide excision repair	9,11E-04	5,40E-03
Glycolysis / Gluconeogenesis	1,09E-03	6,03E-03
Peptidoglycan biosynthesis	1,74E-03	9,04E-03
Amino sugar and nucleotide sugar metabolism	2,16E-03	1,06E-02
Alanine, aspartate and glutamate metabolism	3,15E-03	1,45E-02
RNA degradation	3,47E-03	1,52E-02
Aminoacyl-tRNA biosynthesis	4,30E-03	1,79E-02
Terpenoid backbone biosynthesis	5,53E-03	2,19E-02
Streptomycin biosynthesis	6,31E-03	2,38E-02
Polyketide sugar unit biosynthesis	9,36E-03	3,27E-02
Lysine biosynthesis	9,45E-03	3,27E-02
Propanoate metabolism	1,03E-02	3,41E-02
Quorum sensing	1,13E-02	3,56E-02
Homologous recombination	1,20E-02	3,56E-02
Methane metabolism	1,20E-02	3,56E-02
Mismatch repair	1,37E-02	3,91E-02
DNA replication	1,50E-02	4,16E-02
