# Supplementary material for: Comparative proteomic analysis of four biotechnological strains Lactococcus lactis through label‐free quantitative proteomics
Source: Microb Biotechnol. 2018 Oct 19;12(2):265–74. doi: 10.1111/1751-7915.13305 (PMC6389847; doi:10.1111/1751-7915.13305)
Supplement: Supplementary file 6 — Table S5. Total list of proteins detected in the Shared genome/proteome. [file MBT2-12-265-s006.pdf]

Supplementary File 8: Total list of proteins detected in the Shared genome/proteome

	Genome			Ortology	Description	Expression	COG function	Proteins identified by LC/MSE			
**MG1363**	**NZ9000**	**NCDO2118**	**IL1403**		**Produto**			**MG1363**	**NZ9000**	**NCDO2118**	**IL1403**
llmg_pseudo_08		NCDO2118_RS01400	L0008	Shared	Enolase	Shared	G			NCDO2118_RS01400	L0008
llmg_pseudo_42		NCDO2118_RS06395	L0074	Shared	3-isopropylmalate dehydrogenase	Exclusive	C			NCDO2118_RS06395	
llmg_1820	LLNZ_09370		L0097	Shared	aspartate kinase	Shared	E	llmg_1820	LLNZ_09370		
llmg_0658	LLNZ_03405		L0308	Shared	type I restriction-modification system restriction subunit	Shared	L	llmg_0658	LLNZ_03405		
	LLNZ_01810	NCDO2118_RS01945	L126409	Shared	Transcriptional regulator	Shared	K	LLNZ_01810	LLNZ_01810	NCDO2118_RS01945	L126409
llmg_1070	LLNZ_05535		L13958	Shared	Hypothetical protein	Exclusive	S		LLNZ_05535		
llmg_pseudo_09		NCDO2118_RS02035	L145267	Shared	ABC transporter substrate-binding protein	Exclusive	U			NCDO2118_RS02035	
llmg_pseudo_80		NCDO2118_RS10625	L177593	Shared	Cysteine desulfhydrase	Exclusive	R			NCDO2118_RS10625	
llmg_1096	LLNZ_05670		L191051	Shared	Hypothetical protein	Exclusive	S	llmg_1096	LLNZ_05670		
llmg_1093	LLNZ_05655		L193176	Shared	Hypothetical protein	Shared	S	llmg_1093	LLNZ_05655		
llmg_0793	LLNZ_04110		L38687	Shared	Hypothetical protein	Shared	S	llmg_0793	LLNZ_04110		
llmg_0048	LLNZ_00245		L45702	Shared	Ps108 protein	Shared	S	llmg_0048	LLNZ_00245		
llmg_0646	LLNZ_03335		L80399	Shared	intercellular adhesion protein A	Shared	M	llmg_0646	LLNZ_03335		
llmg_0753	LLNZ_03920	NCDO2118_RS09165	MENG_L/	Shared	Demethylmenaquinone methyltransferase	Shared	H	llmg_0753	LLNZ_03920	NCDO2118_RS09165	
llmg_0094	LLNZ_00470	NCDO2118_RS00315	x	Shared	Hypothetical protein	Exclusive	S			NCDO2118_RS00315	NCDO2118_RS00315
llmg_0146	LLNZ_00770	NCDO2118_RS00615	x	Shared	Aldo/keto reductase	Exclusive	Q			NCDO2118_RS00615	NCDO2118_RS00615
llmg_0188	LLNZ_00980	NCDO2118_RS00870	x	Shared	Hypothetical protein	Shared	S	llmg_0188	LLNZ_00980	NCDO2118_RS00870	NCDO2118_RS00870
llmg_1255	LLNZ_06465	NCDO2118_RS01415	x	Shared	Cold-shock protein	Exclusive	K			NCDO2118_RS01415	NCDO2118_RS01415
llmg_0346	LLNZ_01815	NCDO2118_RS01950	x	Shared	Iron ABC transporter ATP-binding protein	Exclusive	P			NCDO2118_RS01950	NCDO2118_RS01950
llmg_0573	LLNZ_02940	NCDO2118_RS03065		Shared	Inorganic pyrophosphatase	Exclusive	P			NCDO2118_RS03065	
llmg_1630	LLNZ_08390	NCDO2118_RS04760	x	Shared	Hypothetical protein	Exclusive	S			NCDO2118_RS04760	NCDO2118_RS04760
	LLNZ_07585	NCDO2118_RS05740	x	Shared	Hypothetical protein	Exclusive	S			NCDO2118_RS05740	NCDO2118_RS05740
		NCDO2118_RS07020	L141530	Shared	Esterase	Shared	E			NCDO2118_RS07020	L141530
		NCDO2118_RS07790	L122924	Shared	Endo-beta-N-acetylglucosaminidase	Exclusive	G			NCDO2118_RS07790	
		NCDO2118_RS07800	L126168	Shared	Lacto-N-biosidase	Shared	G			NCDO2118_RS07800	L126168
		NCDO2118_RS06535		Shared	Sugar ABC transporter ATP binding protein	Exclusive	G			NCDO2118_RS06535	
		NCDO2118_RS01090	L19816	Shared	Glycerol-3-phosphate cytidiltransferase	Shared	I			NCDO2118_RS01090	L19816
		NCDO2118_RS04665	L109379	Shared	LysR family transcriptional regulator	Shared	K			NCDO2118_RS04665	L109379
		NCDO2118_RS01440	L65637	Shared	Transcriptional regulator	Shared	K			NCDO2118_RS01440	L65637
		NCDO2118_RS04920	L106117	Shared	Antirepressor	Shared	R			NCDO2118_RS04920	L106117
		NCDO2118_RS01055	L12335	Shared	UDP-glucose 4-epimerase	Shared	R			NCDO2118_RS01055	L12335
		NCDO2118_RS03895	L145739	Shared	Flotillin-like protein	Shared	R			NCDO2118_RS03895	L145739
		NCDO2118_RS01080	L17695	Shared	Sugar transferase	Shared	R			NCDO2118_RS01080	L17695
		NCDO2118_RS11810	L19128	Shared	Radical SAM protein	Shared	R			NCDO2118_RS11810	L19128
		NCDO2118_RS06660	L78730	Shared	Lactate oxidase	Exclusive	R			NCDO2118_RS06660	
		NCDO2118_RS04915	L105494	Shared	Hypothetical protein	Exclusive	S			NCDO2118_RS04915	
		NCDO2118_RS07025	L142410	Shared	Hypothetical protein	Shared	S			NCDO2118_RS07025	L142410
		NCDO2118_RS03890	L145357	Shared	Hypothetical protein	Shared	S			NCDO2118_RS03890	L145357
		NCDO2118_RS10740	L15484	Shared	Hypothetical protein	Exclusive	S			NCDO2118_RS10740	
		NCDO2118_RS02960	L178908	Shared	Hypothetical protein	Shared	S			NCDO2118_RS02960	L178908
		NCDO2118_RS09975	L197116	Shared	Hypothetical protein	Shared	S			NCDO2118_RS09975	L197116
		NCDO2118_RS09995	L199277	Shared	Hypothetical protein	Shared	S			NCDO2118_RS09995	L199277
		NCDO2118_RS09680	L3255	Shared	Hypothetical protein	Shared	S			NCDO2118_RS09680	L3255
		NCDO2118_RS03410	L86826	Shared	Hypothetical protein	Shared	S			NCDO2118_RS03410	L86826
		NCDO2118_RS12300	L96658	Shared	Hypothetical protein	Shared	S			NCDO2118_RS12300	L96658
		NCDO2118_RS09970	x	Shared	Hypothetical protein	Exclusive	S			NCDO2118_RS09970	NCDO2118_RS09970
llmg_1163	LLNZ_05995	NCDO2118_RS06940	x	Shared	Sugar ABC transporter substrate-binding protein	Exclusive	G			NCDO2118_RS06940	NCDO2118_RS06940
llmg_1162	LLNZ_05990	NCDO2118_RS06945	x	Shared	9-O-acetylesterase	Shared	G	llmg_1162	LLNZ_05990	NCDO2118_RS06945	NCDO2118_RS06945
llmg_0851	LLNZ_04380	NCDO2118_RS07435	x	Shared	1,4-beta-N-acetylmuramidase	Exclusive	M			NCDO2118_RS07435	NCDO2118_RS07435
	LLNZ_04875	NCDO2118_RS08135	x	Shared	Hypothetical protein	Shared	S	LLNZ_04875	LLNZ_04875	NCDO2118_RS08135	NCDO2118_RS08135
llmg_0865	LLNZ_04450	NCDO2118_RS08930	x	Shared	Transcriptional antiterminator	Exclusive	K			NCDO2118_RS08930	NCDO2118_RS08930
llmg_0706	LLNZ_03670	NCDO2118_RS09265		Shared	Hypothetical protein	Exclusive	S			NCDO2118_RS09265	
llmg_1990	LLNZ_10270	NCDO2118_RS09915	x	Shared	Hypothetical protein	Exclusive	S			NCDO2118_RS09915	NCDO2118_RS09915
llmg_2161	LLNZ_11145	NCDO2118_RS10500	x	Shared	Cyclopropane-fatty-acyl-phospholipid synthase	Shared	I	llmg_2161	LLNZ_11145	NCDO2118_RS10500	NCDO2118_RS10500
llmg_2211	LLNZ_11405	NCDO2118_RS10775	x	Shared	Hypothetical protein#	Shared	S	llmg_2211	LLNZ_11405	NCDO2118_RS10775	NCDO2118_RS10775
llmg_2279	LLNZ_11785	NCDO2118_RS11015	x	Shared	ABC transporter ATP-binding protein	Shared	V	llmg_2279	LLNZ_11785	NCDO2118_RS11015	NCDO2118_RS11015
llmg_2352	LLNZ_12160	NCDO2118_RS11365	x	Shared	UDP-N-acetylmuramoyl-tripeptide--D-alanyl-D- alanine ligase	Shared	M		LLNZ_12160	NCDO2118_RS11365	NCDO2118_RS11365
llmg_0053	LLNZ_00265	NCDO2118_RS12045	x	Shared	Bacteriocin	Exclusive	V			NCDO2118_RS12045	NCDO2118_RS12045
llmg_0711	LLNZ_03700	NCDO2118_RS03555		Shared	DNA-invertase/resolvase	Shared	L	llmg_0711	LLNZ_03700		
llmg_0224	LLNZ_01165	NCDO2118_RS04835		Shared	UDP-galactopyranose mutase	Shared	M	llmg_0224	LLNZ_01165		
	LLNZ_01675	NCDO2118_RS06590		Shared	Nisin biosynthesis two-component system, response regulator NisR	Exclusive	T	LLNZ_01675	LLNZ_01675		
llmg_1139	LLNZ_05880	NCDO2118_RS07095		Shared	Hypothetical protein	Shared	S	llmg_1139	LLNZ_05880		
llmg_0846	LLNZ_04355	NCDO2118_RS07460		Shared	Hypothetical protein	Shared	S	llmg_0846	LLNZ_04355		
llmg_1011	LLNZ_05210	NCDO2118_RS07750		Shared	ABC-type sugar transport system, periplasmic component	Shared	U	llmg_1011	LLNZ_05210		
llmg_1244	LLNZ_06405			Shared	PTS system, lactose/cellobiose family IIC component	Shared	G	llmg_1244	LLNZ_06405		
llmg_1616	LLNZ_08315			Shared	UDP-glucose 6-dehydrogenase	Shared	G	llmg_1616	LLNZ_08315		
llmg_1456	LLNZ_07505			Shared	beta-glucosidase	Shared	G	llmg_1456	LLNZ_07505		
llmg_0221	LLNZ_01150			Shared	glycosyl transferase	Shared	G	llmg_0221	LLNZ_01150		
llmg_2248	LLNZ_11595			Shared	putative abortive phage resistance	Shared	V	llmg_2248	LLNZ_11595		
llmg_0834	LLNZ_04295			Shared	Hypothetical protein	Shared	S	llmg_0834	LLNZ_04295		
llmg_1358	LLNZ_07020			Shared	Hypothetical protein	Shared	S	llmg_1358	LLNZ_07020		
llmg_0482	LLNZ_02495			Shared	Hypothetical protein	Shared	S	llmg_0482	LLNZ_02495		
llmg_0057	LLNZ_00285			Shared	Hypothetical protein	Shared	S	llmg_0057	LLNZ_00285		
llmg_0131	LLNZ_00675			Shared	Hypothetical protein	Shared	S	llmg_0131	LLNZ_00675		
llmg_0602	LLNZ_03090			Shared	Hypothetical protein	Shared	S	llmg_0602	LLNZ_03090		
llmg_1399	LLNZ_07215			Shared	Hypothetical protein	Shared	S	llmg_1399	LLNZ_07215		
llmg_1253	LLNZ_06455			Shared	Hypothetical protein	Shared	S	llmg_1253	LLNZ_06455		
llmg_1386	LLNZ_07160			Shared	Hypothetical protein	Shared	S	llmg_1386	LLNZ_07160		
llmg_0791	LLNZ_04100			Shared	Hypothetical protein	Shared	S	llmg_0791	LLNZ_04100		
llmg_1357	LLNZ_07015			Shared	Hypothetical protein	Shared	S	llmg_1357	LLNZ_07015		
llmg_0223	LLNZ_01160			Shared	Hypothetical protein	Shared	S	llmg_0223	LLNZ_01160		
llmg_0222	LLNZ_01155			Shared	Hypothetical protein	Shared	S	llmg_0222	LLNZ_01155		
llmg_1723	LLNZ_08900			Shared	putative secreted protein	Shared	S	llmg_1723	LLNZ_08900		
llmg_2262	LLNZ_11675			Shared	Hypothetical protein	Shared	S	llmg_2262	LLNZ_11675		
llmg_2002	LLNZ_10320			Shared	Hypothetical protein	Shared	S	llmg_2002	LLNZ_10320		
llmg_0813	LLNZ_04195			Shared	Hypothetical protein	Shared	S	llmg_0813	LLNZ_04195		
llmg_1173	LLNZ_06040			Shared	Hypothetical protein	Shared	S	llmg_1173	LLNZ_06040		
llmg_0284	LLNZ_01485			Shared	Hypothetical protein	Shared	S	llmg_0284	LLNZ_01485		
llmg_0898	LLNZ_04615			Shared	Hypothetical protein	Shared	S	llmg_0898	LLNZ_04615		
llmg_1506	LLNZ_07750			Shared	Hypothetical protein	Shared	S	llmg_1506	LLNZ_07750		
llmg_2554	LLNZ_13200			Shared	Hypothetical protein	Shared	S	llmg_2554	LLNZ_13200		
llmg_0009	LLNZ_00045			Shared	putative secreted protein	Shared	S	llmg_0009	LLNZ_00045		
llmg_1366	LLNZ_07065			Shared	Hypothetical protein	Shared	S	llmg_1366	LLNZ_07065		
llmg_2247	LLNZ_11590			Shared	Hypothetical protein	Shared	S	llmg_2247	LLNZ_11590		
llmg_1356	LLNZ_07010			Shared	Hypothetical protein	Shared	S	llmg_1356	LLNZ_07010		
llmg_1402	LLNZ_07230			Shared	Hypothetical protein	Shared	S	llmg_1402	LLNZ_07230		
llmg_1148	LLNZ_05925			Shared	Hypothetical protein	Shared	S	llmg_1148	LLNZ_05925		
llmg_0226	LLNZ_01175			Shared	Surface protein (strain NZ9000) GN=LLNZ_01175 SV=1	Shared	S	llmg_0226	LLNZ_01175		
llmg_1354	LLNZ_07000			Shared	Hypothetical protein	Shared	S	llmg_1354	LLNZ_07000		
llmg_0734	LLNZ_03820			Shared	putative amidase (strain NZ9000) GN=LLNZ_03820 SV=1	Shared	R	llmg_0734	LLNZ_03820		
llmg_1350	LLNZ_06980			Shared	putative tellurium resistance protein	Shared	P	llmg_1350	LLNZ_06980		
llmg_1353	LLNZ_06995			Shared	putative tellurite resistance protein	Shared	P	llmg_1353	LLNZ_06995		
llmg_1352	LLNZ_06990			Shared	putative tellurium resistance protein	Shared	P	llmg_1352	LLNZ_06990		
llmg_1351	LLNZ_06985			Shared	putative tellurium resistance protein	Shared	P	llmg_1351	LLNZ_06985		
llmg_0655	LLNZ_03390			Shared	transposase	Shared	X	llmg_0655	LLNZ_03200		
llmg_1615	LLNZ_08310			Shared	putative acetyltransferase	Shared	Q	llmg_1615	LLNZ_08310		
llmg_2139	LLNZ_11035			Shared	cI-like repressor	Shared	K	llmg_2139	LLNZ_11035		
llmg_0089	LLNZ_00445			Shared	HTH-type transcriptional regulator	Shared	K	llmg_0089	LLNZ_00445		
llmg_1846	LLNZ_09500			Shared	cold shock-like protein cspB	Shared	K	llmg_1846	LLNZ_09500		
llmg_0088	LLNZ_00435			Shared	hypothetical protein	Shared	S	llmg_0088	LLNZ_00435		
llmg_0441	LLNZ_02290			Shared	6-phospho-beta-glucosidase	Shared	G	llmg_0441	LLNZ_02290		
llmg_0701	LLNZ_03645			Shared	oligopeptide-binding protein oppA precursor	Shared	E	llmg_0701	LLNZ_03645		
llmg_1238	LLNZ_06375			Shared	cold shock protein cspD	Shared	K	llmg_1238	LLNZ_06375		
llmg_1347	LLNZ_06965			Shared	hypothetical protein	Shared	S	llmg_1347	LLNZ_06965		
llmg_1347	LLNZ_06970			Shared	hypothetical protein	Shared	S	llmg_1347	LLNZ_06970		
llmg_2332	LLNZ_12045			Shared	glutamyl-tRNA synthetase	Shared	J	llmg_2332	LLNZ_12045		
llmg_0802	LLNZ_04145			Shared	Hypothetical protein	Exclusive	S		LLNZ_04145		

COG groups are defined in the legend to Fig. 2B.
