# Supplementary material for: Comparative proteomic analysis of four biotechnological strains Lactococcus lactis through label‐free quantitative proteomics
Source: Microb Biotechnol. 2018 Oct 19;12(2):265–74. doi: 10.1111/1751-7915.13305 (PMC6389847; doi:10.1111/1751-7915.13305)
Supplement: Supplementary file 7 — Table S6. Proteins unique identified in NCDO2118. [file MBT2-12-265-s007.pdf]

Supplementary File 9: Proteins unique identified in NCDO2118.

Locus tag	Produto	COG Function
NCDO2118_RS00555	Hypothetical protein	S
NCDO2118_RS04905	Endonuclease	L
NCDO2118_RS06235	Hypothetical protein	S
NCDO2118_RS03435	Sucrose-6-phosphate hydrolase	G
NCDO2118_RS06260	Hypothetical protein	S
NCDO2118_RS06300	Potassium-transporting ATPase subunit B	P
NCDO2118_RS07360	Hypothetical protein	S
NCDO2118_RS12050	Phage protein	X
NCDO2118_RS07300	Hypothetical protein	S
NCDO2118_RS01520	Transcriptional regulator	K
NCDO2118_RS06285	Histidine kinase	Q
NCDO2118_RS08740	Hypothetical protein	S
NCDO2118_RS08715	Hypothetical protein	S
NCDO2118_RS01545	Hypothetical protein	S
NCDO2118_RS01840	XRE family transcriptional regulator	K
NCDO2118_RS06615	Nisin biosynthesis protein NisB	R
NCDO2118_RS06965	Cro/Cl family transcriptional regulator	K
NCDO2118_RS06290	XRE family transcriptional regulator	K
NCDO2118_RS03540	Hypothetical protein	S
NCDO2118_RS00740	LysR family transcriptional regulator	K
NCDO2118_RS10045	Hypothetical protein	S
NCDO2118_RS12150	Phage protein	X
NCDO2118_RS08245	Transcription antiterminator BglG	K
NCDO2118_RS00545	Thymidylate kinase	F
NCDO2118_RS04930	Hypothetical protein	S
NCDO2118_RS10020	Hypothetical protein	S
NCDO2118_RS01550	Transposase	X
NCDO2118_RS01510	GntR family transcriptional regulator	K
NCDO2118_RS04955	Hypothetical protein	S
NCDO2118_RS04900	Hypothetical protein	S
NCDO2118_RS03155	ATP-dependent helicase	L
NCDO2118_RS04925	Hypothetical protein	S
NCDO2118_RS00690	Tyrosine protein kinase	E
NCDO2118_RS07715	Putrescine--2-oxoglutarate aminotransferase	E
NCDO2118_RS01460	XRE family transcriptional regulator	K
NCDO2118_RS03440	PTS sucrose transporter subunit IIABC	G
NCDO2118_RS04830	Polysaccharide biosynthesis protein	D
NCDO2118_RS00735	Exopolysaccharide biosynthesis protein	M
NCDO2118_RS00695	Tyrosine protein phosphatase	T

COG groups are defined in the legend to Fig. 2B.
